# Evidence-based umbrella review of 162 peripheral biomarkers for major mental disorders

**DOI:** 10.1038/s41398-020-0835-5

**Published:** 2020-05-18

**Authors:** André F. Carvalho, Marco Solmi, Marcos Sanches, Myrela O. Machado, Brendon Stubbs, Olesya Ajnakina, Chelsea Sherman, Yue Ran Sun, Celina S. Liu, Andre R. Brunoni, Giorgio Pigato, Brisa S. Fernandes, Beatrice Bortolato, Muhammad I. Husain, Elena Dragioti, Joseph Firth, Theodore D. Cosco, Michael Maes, Michael Berk, Krista L. Lanctôt, Eduard Vieta, Diego A. Pizzagalli, Lee Smith, Paolo Fusar-Poli, Paul A. Kurdyak, Michele Fornaro, Jürgen Rehm, Nathan Herrmann

**Affiliations:** 1 Department of Psychiatry, University of Toronto, Toronto, ON Canada; 2 Centre for Addiction & Mental Health (CAMH), Toronto, ON Canada; 3 Neuroscience Department, University of Padova, Padova, Italy; 4 Neuroscience Center, University of Padova, Padova, Italy; 5 Early Psychosis: Interventions and Clinical-detection (EPIC) lab, Department of Psychosis Studies, Institute of Psychiatry, Psychology & Neuroscience, King’s College London, London, UK; 6 Centre for Addiction & Mental Health (CAMH), Toronto, ON Canada; 7 Krembil Centre for NeuroInformatics, Toronto, ON Canada; 8 Division of Dermatology, Women’s College Hospital, Toronto, ON Canada; 9 Physiotherapy Department, South London and Maudsley NHS Foundation Trust, London, UK; 10 Health Service and Population Research Department, Institute of Psychiatry, Psychology and Neuroscience, King’s College London, De Crespigny Park, London, UK; 11 Department of Biostatistics & Health Informatics, Institute of Psychiatry, Psychology and Neuroscience, King’s College London, London, UK; 12 Neuropsychopharmacology Research Group, Hurvitz Brain Sciences Program, Sunnybrook Research Institute, Toronto, ON Canada; 13 Service of Interdisciplinary Neuromodulation, Laboratory of Neurosciences (LIM-27) and National Institute of Biomarkers in Psychiatry (INBioN), Department and Institute of Psychiatry, University of São Paulo, São Paulo, SP Brazil; 14 Department of Internal Medicine, Faculdade de Medicina da Universidade de São Paulo, São Paulo, Brazil; 15 Neuroscience Department, University of Padova, Padova, Italy; 16 Neuroscience Center, University of Padova, Padova, Italy; 17 Department of Psychiatry and Behavioral Sciences, The University of Texas Health Science Center, Houston, TX USA; 18 Department of Mental Health ULSS 8 “Berica”, Vicenza, Italy; 19 Department of Psychiatry, University of Toronto, Toronto, ON Canada; 20 Centre for Addiction & Mental Health (CAMH), Toronto, ON Canada; 21 Pain and Rehabilitation Centre, and Department of Medical and Health Sciences, Linköping University, SE-581 85 Linköping, Sweden; 22 NICM Health Research Institute, Western Sydney University, Westmead, Australia; 23 Division of Psychology and Mental Health, Faculty of Biology, Medicine and Health, University of Manchester, Manchester, UK; 24 Gerontology Research Center, Simon Fraser University, Vancouver, Canada; 25 Oxford Institute of Population Ageing, University of Oxford, Oxford, UK; 26 Department of Psychiatry, Faculty of Medicine, Chulalongkorn University, Bangkok, Thailand; 27 IMPACT Strategic Research Center, Deakin University, Geelong, Australia; 28 Orygen, the National Centre of Excellence in Youth Mental Health, Melbourne, VIC Australia; 29 Centre for Youth Mental Health, University of Melbourne, Melbourne, VIC Australia; 30 Florey Institute for Neuroscience and Mental Health, University of Melbourne, Melbourne, VIC Australia; 31 Department of Psychiatry, University of Toronto, Toronto, ON Canada; 32 Centre for Addiction & Mental Health (CAMH), Toronto, ON Canada; 33 Neuropsychopharmacology Research Group, Hurvitz Brain Sciences Program, Sunnybrook Research Institute, Toronto, ON Canada; 34 Sunnybrook Research Institute, Toronto, ON Canada; 35 Department of Pharmacology and Toxicology, University of Toronto, Toronto, ON Canada; 36 Psychiatry and Psychology Department of the Hospital Clinic, Institute of Neuroscience, University of Barcelona, IDIBAPS, CIBERSAM, Barcelona, Catalonia Spain; 37 Department of Psychiatry & McLean Hospital, Harvard Medical School, Belmont, MA 02478 USA; 38 The Cambridge Centre for Sport and Exercise Sciences, Anglia Ruskin University, Cambridge, UK; 39 Early Psychosis: Interventions and Clinical-detection (EPIC) lab, Department of Psychosis Studies, Institute of Psychiatry, Psychology & Neuroscience, King’s College London, London, UK; 40 OASIS Service, South London and Maudsley National Health Service Foundation Trust, London, UK; 41 Department of Brain and Behavioral Sciences, University of Pavia, Pavia, Italy; 42 Department of Psychiatry, University of Toronto, Toronto, ON Canada; 43 Canada Institute for Clinical Evaluative Sciences (ICES), Toronto, ON Canada; 44 Institute for Mental Health Policy Research, Centre for Addiction and Mental Health (CAMH), Toronto, Canada; 45 Department of Neuroscience, Reproductive Science and Dentistry, Section of Psychiatr, University School of Medicine Federico II, Naples, Italy; 46 Department of Psychiatry, University of Toronto, Toronto, ON Canada; 47 Institute for Mental Health Policy Research, Centre for Addiction and Mental Health (CAMH), Toronto, Canada; 48 Campbell Family Mental Health Research Institute, CAMH, Toronto, Canada; 49 Addiction Policy, Dalla Lana School of Public Health, University of Toronto, Toronto, ON Canada; 50 Institute of Clinical Psychology and Psychotherapy & Center for Clinical Epidemiology and Longitudinal Studies, Technische Universität Dresden, Dresden, Germany; 51 Institute of Medical Science, University of Toronto, Toronto, Canada; 52 Department of International Health Projects, Institute for Leadership and Health Management, I.M. Sechenov First Moscow State Medical University, Moscow, Russian Federation; 53 Department of Psychiatry, University of Toronto, Toronto, ON Canada; 54 Neuropsychopharmacology Research Group, Hurvitz Brain Sciences Program, Sunnybrook Research Institute, Toronto, ON Canada; 55 Sunnybrook Research Institute, Toronto, ON Canada

**Keywords:** Diagnostic markers, Molecular neuroscience

## Abstract

The literature on non-genetic peripheral biomarkers for major mental disorders is broad, with conflicting results. An umbrella review of meta-analyses of non-genetic peripheral biomarkers for Alzheimer’s disease, autism spectrum disorder, bipolar disorder (BD), major depressive disorder, and schizophrenia, including first-episode psychosis. We included meta-analyses that compared alterations in peripheral biomarkers between participants with mental disorders to controls (i.e., between-group meta-analyses) and that assessed biomarkers after treatment (i.e., within-group meta-analyses). Evidence for association was hierarchically graded using a priori defined criteria against several biases. The Assessment of Multiple Systematic Reviews (AMSTAR) instrument was used to investigate study quality. 1161 references were screened. 110 met inclusion criteria, relating to 359 meta-analytic estimates and 733,316 measurements, on 162 different biomarkers. Only two estimates met a priori defined criteria for convincing evidence (elevated awakening cortisol levels in euthymic BD participants relative to controls and decreased pyridoxal levels in participants with schizophrenia relative to controls). Of 42 estimates which met criteria for highly suggestive evidence only five biomarker aberrations occurred in more than one disorder. Only 15 meta-analyses had a power >0.8 to detect a small effect size, and most (81.9%) meta-analyses had high heterogeneity. Although some associations met criteria for either convincing or highly suggestive evidence, overall the vast literature of peripheral biomarkers for major mental disorders is affected by bias and is underpowered. No convincing evidence supported the existence of a trans-diagnostic biomarker. Adequately powered and methodologically sound future large collaborative studies are warranted.

## Introduction

One of the overarching goals of the emerging field of precision psychiatry is to incorporate advanced technologies to provide an objective data-driven personalized approach to the diagnosis and treatment of mental disorders^1,2^. However, unlike other medical fields, there is an acknowledged ‘translational gap’ in psychiatry^1,3^. In parallel, the field of biological psychiatry aiming to provide a neurobiological basis for current mental disorders, has provided contrasting results, even in pivotal biomarkers^4^. Hence, the diagnosis and clinical management of major mental disorders is still entirely based on psychopathological knowledge, while the treatment of mental disorders remains predominantly based on ‘trial and error’, albeit within the confines of fitting evidence-based prescription to a clinical profile^5^.

Over the past two decades the field has witnessed a remarkable increase in interest on biomarkers for mental disorders^6^. In particular, the literature on non-genetic peripheral biomarkers has grown exponentially, with the publication of several systematic reviews and meta-analyses^7–12^. The identification and validation of biomarkers for mental disorders are thought to be crucial steps in the development of precision and biological psychiatry, and its ultimate incorporation in the current landscape of psychiatric care is expected to follow^1^. However, this change is not translating into meaningful modifications in clinical practice.

Several reasons may contribute to the contrast between the overall volume of this literature and the limited applicability of peripheral biomarkers in current psychiatric practice. For instance, it has been proposed that conventional psychiatric diagnoses based, for example, on the Diagnostic and Statistical Manual for Mental Disorders (DSM) may lack biological validity^2,13^. In this respect, it has been proposed that similarly to genetic^14^ and neuroimaging^15,16^ biomarkers, alterations in peripheral biomarkers for major mental disorders may be shared across distinct diagnostic categories, and thus may have a transdiagnostic nature^6^. However, what is a trans-diagnostic construct in psychiatry remains debated, and no study has properly assessed the trans-diagnostic nature of any biomarker with a methodologically sound approach^17^.

In addition to the lack of consensus on how to define a trans-diagnostic construct, a core reason for this translational gap even in a single disorder may be due to the presence of several biases including large heterogeneity, an excess significance bias, as well as a selective reporting of statistically significant (i.e., ‘positive’) findings without proper adjustment to multiple confounders. An Umbrella review systematically evaluates and collects information from multiple systematic reviews and meta-analyses on all outcomes of a given topic for which these have been performed^18^. Umbrella reviews are particularly suited to uncover these biases^19^, as previously demonstrated with respect to peripheral biomarkers for depression^20^, bipolar disorder (BD)^20^, and schizophrenia^21^. However, those previous umbrella reviews have only addressed studies that have differentiated participants with a specific mental disorder and healthy controls, and not changes in peripheral biomarkers following treatment for these disorders. Moreover, those umbrella reviews focused on only one mental disorder each.

Thus, the current work provides a comprehensive umbrella review of meta-analyses of peripheral biomarkers for major mental disorders related to high prevalence and burden, namely Alzheimer’s disease (AD), autism spectrum disorder (ASD), BD, major depressive disorder (MDD), and schizophrenia, including also first-episode psychosis (FEP) stage. We aimed to re-assess the presence of bias in this literature and identify biomarkers that would be supported by most convincing evidence. In addition, we aimed to identify shared and unique alterations in biomarkers for those major mental disorders among those supported by either convincing or highly suggestive evidence. In the current analysis, we considered both studies that investigated abnormalities in peripheral biomarkers of mental disorders compared to controls (i.e., between-group meta-analyses) and ones that assessed alterations in the levels of peripheral biomarkers after treatment (i.e., within-group meta-analyses).

## Methods

### Literature search

We conducted an umbrella review, which is a systematic collection of multiple systematic reviews and meta-analyses done in a specific research topic^22^. The PubMed/MEDLINE database was searched from inception to February 17, 2019 for all available meta-analyses non-genetic peripheral biomarkers for major mental disorders. This search strategy was augmented through (1) handsearching the reference lists of included articles and (2) tracking citations of included articles through the Google Scholar database. The search string used in the current umbrella review was developed by a professional librarian and is available in the Supplementary Online material. The searches, screening, data extraction, and methodological quality appraisal were independently conducted by at least two investigators. Disagreements were resolved through consensus. When a consensus could not be reached a third investigator (AFC) made the final decision. An a priori defined protocol was followed (available upon reasonable request to the corresponding author of the current manuscript).

### Eligibility criteria

We included meta-analyses published in peer-reviewed journals that assessed and synthesized studies on peripheral biomarkers for adults with AD, ASD, BD, MDD, Schizophrenia, including FEP. We included studies in which biomarkers were assayed in participants with a specific mental disorder compared to controls (i.e., between-group meta-analyses), as well as ones which assessed changes in peripheral biomarkers in any of those disorders after treatment (i.e., within-group meta-analyses). Studies published in English were considered for inclusion. This decision was made because most well-designed systematic reviews and meta-analyses are published in English. We included studies in which diagnoses of mental disorders were conducted by means of a validated structured interview based on standard diagnostic criteria such as the *International Classification of Disease* (ICD) or the *Diagnostic and Statistical Manual of Mental Disorders* (DSM). We also considered studies in which a probable diagnosis of a major depressive episode was established through a validated screening questionnaire as well as studies in which a diagnosis of FEP was based on clinical assessment by a mental health care provider. We excluded the following types of studies: (1) systematic reviews without a meta-analytic synthesis of the evidence; (2) animal studies; (3) studies of other types of biomarkers (for example, genetic biomarkers); (4) studies that included participants with two or more diagnoses; (5) studies that included participants with other primary psychiatric diagnoses (e.g. anxiety disorders); (6) studies that investigated biomarkers for other purposes (for example, biomarkers of risk, stage or prognosis)^23^; (7) studies conducted in pediatric samples (except from ASD and FEP); and (8) if there was more than one meta-analysis for the same biomarker in the same population, we considered only the largest MA (i.e., the one with the largest number of included individual studies).

### Data extraction

For each eligible reference, we extracted the first author, year of publication, specific diagnoses assessed, as well as the number of included studies. We also extracted the summary effect size (ES) measure of each meta-analysis considering the ES used in each study. When available, the following variables were extracted at a study-level: number of cases, number of controls, sample size, ES, and study design. In each eligible reference, we only included the primary analyses due to the expected large amount of evidence. However, when included references provided details on the mood state of participants (e.g. mania or bipolar depression), we also extracted this information at an individual-study level.

### Statistical analysis and methodological quality appraisal

Data were analyzed from March 1, 2019 to October 10, 2019. We estimated ESs and 95% confidence intervals (CIs) using both fixed and random-effects modeling^24^. Due to the anticipated high heterogeneity observed in meta-analyses of peripheral biomarkers for major mental disorders, random-effects calculations were considered in this review. When ESs were not provided as standardized mean difference (SMD) metrics (e.g., odds ratio), we converted the primary ESs to SMD^25^. We also estimated the 95% prediction interval, which accounts for between-study heterogeneity and assesses the uncertainty of the effect that would be expected in a new study addressing the same association^26^. For the largest study included in each meta-analytic estimate, we calculated the standard error (SE) of the ES. If the SE of the ES is <0.1, then the 95% CI will be <0.20 (i.e., less than the magnitude of a small ES). We calculated the *I*^2^ metric to quantify between-study heterogeneity. Values ≥50% and ≥75% are indicative of large and very large heterogeneity, respectively^27^. To assess evidence of small-study effects, we used the asymmetry test developed by Egger et al. ^28^. A *P*-value <0.10 in the Egger’s test and the ES of the largest study being more conservative than the summary random-effects ES of the meta-analysis were considered indicative of small-study effects^20^. We also annotated whether the association reported in each meta-analytic estimate was nominally significant at a *P* < 0.05 level as well as at a *P* < 0.005 level. The level of *P* < 0.005 has been proposed as a more stringent level of significance that could increase the reproducibility of many fields^29^.

We also determined whether the meta-analysis had a statistical power ≥ 80% to detect either a small (i.e., ES ≥ 0.2) or a medium (i.e., ES ≥ 0.5). We used the method described in detail elsewhere^30^. Finally, we also assessed evidence of excess of significance bias with the Ioannidis test^31^. Briefly, this test estimates whether the number of studies with nominally significant results (i.e., *P* < 0.05) among those included in a meta-analysis is too large considering their power to detect significant effects at an alpha level of 0.05. First, the power of each study is estimated with a non-central *t* distribution. The sum of all power estimates provides the expected (E) number of datasets with nominal statistical significance. The actual observed (O) number of statistically significant datasets is then compared to the E number using a *χ*^2^-based test^31^. Since the true ES of a meta-analysis cannot be precisely determined, we considered the ES of the largest dataset as the plausible true ES. This decision was based on the fact that simulations indicate that the most appropriate assumption is the ES of the largest dataset included in the meta-analysis^32^. Excess significance for a single meta-analysis was considered if *P* < 0.10 in Ioannidis’s test and O > E^20^. We graded the credibility of each association according to the following categories: convincing (class I), highly suggestive (class II), suggestive (class III), weak evidence (class IV), and non-significant associations (Table S1).

For evidence supported by either class I or class II evidence, we used credibility ceilings, which is which is a method of sensitivity analyses to account for potential methodological limitations of observational studies that might lead to spurious precision of combined effect estimates. In brief, this method assumes that every observational study has a probability *c* (credibility ceiling) that the true ES is in a different direction from the one suggested by the point estimate^33^. The pooled ESs were estimated considering a wide range of credibility ceilings. All analyses were conducted in STATA/MP 14.0 (StataCorp, USA) with the metan package.

The methodological quality of included systematic reviews and meta-analyses was also appraised using the *Assessment of Multiple Systematic Reviews* (AMSTAR) instrument, which has been validated for this purpose^34,35^. Scores range from 0 to 11 with higher scores indicating greater quality. The AMSTAR tool involves dichotomous scoring (i.e. 0 or 1) of 11 items related to assess methodological rigor of systematic reviews and meta-analyses (e.g., comprehensive search strategy, publication bias assessment). AMSTAR scores are graded as high (8–11), medium (4–7) and low quality (0–3)^34^.

## Results

**Fig. 1:**
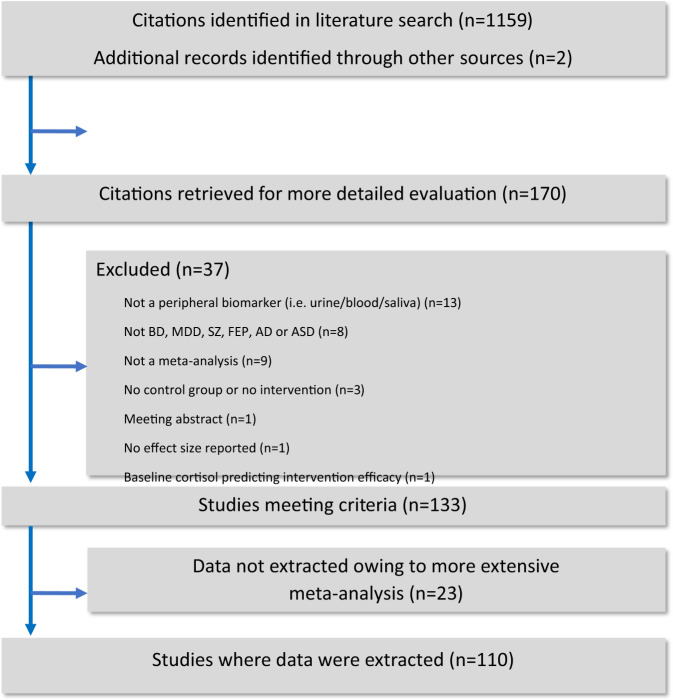
Study flowchart.

Our search strategy identified 1161 unique references of which 991 were excluded after title/abstract screening and 170 underwent full-text review (Fig. 1). Therefore, 110 references met inclusion criteria^7–11,36–139^, and 60 references were excluded with reasons (Table S2). In the 110 included references, there were 81 between-group meta-analytic estimates for MDD, 79 for AD, 62 for schizophrenia, 45 for ASD, 37 for BD, and 15 for FEP. In addition, there were 25 within-group meta-analytic estimates for MDD, 13 for Schizophrenia, and 2 for BD (Mania) (Table S3). In total, there were 247,678 biomarker measurements estimates in cases and 476,340 assays in controls across between-group meta-analyses, while there were 9298 biomarker measurements across within-group meta-analytic estimates (Table S3). One hundred and ninety meta-analytic estimates were statistically significant at a *P*-value < 0.05, whilst 109 were significant at a *P*-value < 0.005 (Table S3).

### Power of meta-analyses

Fifteen between-group meta-analytic estimates had an estimated power >0.8 to detect a small ES, and 145 meta-analyses (126 between-group meta-analyses) had an estimated power >0.8 to detect a medium ES (Table S3).

### Heterogeneity and prediction intervals

No evidence of large heterogeneity (i.e., *I*^2^ < 50%) was found in 65 meta-analyses (18.1%), whilst 294 (81.9%) meta-analytic estimates had evidence of large heterogeneity (i.e., *I*^2^ > 50%). The prediction interval crossed the null value in 341 (94.9%) meta-analytic associations, while prediction intervals of 20 (5.0%) meta-analyses did not cross the null value (Table S3).

### Small-study effects and excess significance bias

Evidence of small-study effects, which is an indication of publication bias, was observed in 38 (10.6%) meta-analyses, whilst evidence of excess of significance bias was verified in 74 (20.6%) meta-analytic estimates (Tables S3).

### Grading of the evidence

Only 2 (0.5%) meta-analytic estimates exhibited class I evidence (83, 119). In euthymic BD participants there was an increase in basal cortisol awakening levels (Hedges’g = 0.25; 95% CI: 0.15–0.35, *P* < 0.005) compared to controls^87^. Participants with schizophrenia presented decreased Vitamin B6 (pyridoxal) levels relative to controls^123^. In addition, 42 (11.7%) meta-analytic estimates were supported by class II evidence, of which 3 were derived from within-group meta-analyses (Table 1). Among those estimates, C-reactive protein levels were increased in euthymic BD, bipolar mania, and in MDD relative to controls^80,102^. In addition, soluble interleukin-(IL)-2 receptor (sIL-2R) levels were increased in MDD and in schizophrenia relative to controls^7,8^. Moreover, levels of antibodies against the N-methyl-d-aspartate receptor (NMDA-R) were elevated in BD and in schizophrenia relative to controls^85^. Brain-derived neurotrophic factor (BDNF) levels were decreased in AD and in MDD^44,110^. Furthermore, levels of insulin-like growth factor-1 (IGF-1) were elevated in bipolar mania and in MDD relative to controls^84^. The remaining findings supported by type II evidence were unique to a single disorder (Table 1).

**Table 1:** Peripheral biomarkers supported by convincing and highly suggestive evidence across major mental disorders.

Biomarker (ref. no.)	Alzheimer’s disease	Autism spectrum disorder	Bipolar disorder	Major depressive disorder	First-episode psychosis	Schizophrenia
*Between-group meta-analyses*						
Adiponectin^166^						**↓**
Anti-Gliadin IgA^118^						**↑**
Apolipoprotein E^167^	**↓**					
Arachidonic acid^a^ ^101^						**↑**
BDNF^44,110^	**↓**			**↓**		
Cortisol^168^						**↑**
Cortisol awakening response^119^					**↓**	
Basal cortisol awakening^b^ ^87^			**↑**			
CRP^80,102^			**↑**^**c**^	**↑**		
Fibroblast growth factor-2^111^				**↑**		
Glutamate^91^				**↑**		
IGF-1^84^			**↑**^**d**^	**↑**		
IL-6^8^				**↑**		
TGF-Beta 1^11^		**↑**				
sIL-2 receptor^7,8^				**↑**		**↑**
TNF-Alpha^8^				**↑**		
Folate^105^						**↓**
Folic acid^59^	**↓**					
Malondialdehyde^109^						**↑**
Nerve growth Factor^122^						**↓**
NMDAR^85^			**↑**			**↑**
Total cholesterol^94^				**↓**		
Copper^46^	**↑**					
Vitamin E^36^	**↓**					
Vitamin B6^b^ ^123^						**↓**
KYNA/3HK^75^				**↓**		
KYNA/QUIN^75^				**↓**		
KYN-ACID^75^				**↓**		
Neurotrophin-3^82^			**↑**			
Uric acid^81^			**↑**			
5-hydroxytryptamine^64^		**↑**				
Glutathione (fasting)^62^		**↓**				
GSSG^69^		**↑**				
GSSG (fasting)^62^		**↑**				
Homocysteine^59^	**↑**					
Within-group Meta-analyses						
Adiponectin^166^						**↓**
IL-6^9^				**↓**		
Lipid peroxidation Markers^138^				**↑**		

*BDNF* brain-derived neurotrophic factor, *IGF* insulin-like growth factor, *IL* interleukin, *INF* interferon, *GSH* glutathione, *GSSG* glutathione disulfide, *KYN acid* kynurenic acid, *Quin* quinolinic acid, *MDA* malondialdehyde, *NMDAR* N-methyl-d-aspartate receptor antibody seropositivity, *NGF* nerve growth factor, *NT* neurotrophin, *QUIN* quinolinic acid, *sIL-2 Receptor* soluble interleukin 2 receptor, *TGF* transforming growth factor, *TNF* tumor necrosis factor, *3HK* 3-hydroxykynurenine.

^a^Source: Red blood cells.

^b^Convincing evidence criteria. Others biomarkers are supported by highly suggestive evidence.

^c^Euthymia and Mania.

^d^Mania.

Of the 44 biomarkers supported by either type I or type II evidence, 37 (84.1%) survived 10% credibility ceilings (Table 2).

**Table 2:** Sensitivity analysis using credibility ceilings for the meta-analyses investigating the associations between biomarkers and Alzheimer disease, autism, bipolar disorder, depression, first episode psychosis, schizophrenia.

Biomarker	Credibility ceiling 10%	Credibility ceiling 20%	Credibility ceiling 30%
Convincing evidence criteria			
*Bipolar disorder*			
Basal cortisol awakening^87^	0.23 (0.07–0.38)	0.19 (−0.01 to 0.40)	0.14 (−0.12 to 0.41)
*Schizophrenia*			
Vitamin B6^123^	−0.46 (−0.78 to −0.15)	−0.46 (−0.95 to 0.02)	−0.46 (−1.24 to 0.31)
Highly suggestive evidence criteria			
*Alzheimer disease*			
Apolipoprotein E^42^	−0.20 (−0.35 to −0.04)	−0.13 (−0.33 to 0.07)	−0.06 (−0.29 to 0.17)
BDNF^44^	−0.09 (−0.23 to 0.05)	−0.03 (−0.14 to 0.08)	−0.01 (−0.14 to 0.12)
Copper^46^	0.17 (0.04–0.30)	0.09 (−0.05 to 0.24)	0.05 (−0.14 to 0.25)
Folic acid^59^	−0.18 (−0.28 to −0.08)	−0.12 (−0.23 to −0.01)	−0.08 (−0.23 to 0.07)
Homocysteine^59^	0.41 (0.28–0.53)	0.40 (0.21–0.59)	0.40 (0.10–0.70)
Vitamin E^36^	−0.20 (−0.31 to −0.08)	−0.13 (−0.26 to −0.01)	−0.09 (−0.23 to 0.06)
*Autism*			
5HT^64^	0.48 (0.26–0.69)	0.35 (0.08–0.62)	0.22 (−0.14 to 0.57)
GSH (fasting)^62^	−1.42 (−2.51 to −0.32)	−1.42 (−3.08 to 0.25)	−1.42 (−4.09 to 1.25)
GSSG^69^	1.07 (0.37–1.78)	1.07 (0.00–2.15)	1.07 (−0.65 to 2.80)
GSSG (fasting)^62^	1.02 (0.31–1.73)	1.02 (−0.07–2.10)	1.02 (−0.72 to 2.75)
Lipid peroxidation markers^138^	0.44 (0.09–0.79)	0.34 (−0.07 to 0.75)	0.32 (−0.29 to 0.93)
TGF-Beta 1^11^	0.35 (0.10–0.59)	0.33 (−0.01 to 0.66)	0.31 (−0.18 to 0.80)
*Bipolar disorder*			
IGF1^84^	0.39 (0.03–0.75)	0.39 (−0.16 to 0.94)	0.39 (−0.49 to 1.27)
NMDAR^85^	0.47 (0.13–0.80)	0.47 (−0.04 to 0.98)	0.47 (−0.35 to 1.29)
NT-3^82^	0.08 (−0.11 to 0.27)	−0.01 (−0.18 to 0.16)	0.00 (−0.21 to 0.20)
Uric acid^81^	0.23 (−0.02 to 0.49)	0.08 (−0.14 to 0.31)	0.03 (−0.20 to 0.27)
CRP* ^80^	0.20 (0.06–0.34)	0.13 (−0.04 to 0.31)	0.12 (−0.14 to 0.39)
CRP** ^80^	0.46 (0.23–0.68)	0.44 (0.11–0.78)	0.43 (−0.08 to 0.93)
*Depression*			
BDNF^110^	−0.18 (−0.30 to −0.05)	−0.07 (−0.19 to 0.05)	−0.03 (−0.18 to 0.12)
CRP^80^	0.43 (0.26–0.61)	0.42 (0.16–0.67)	0.42 (0.02–0.82)
Fibroblast growth factor-2^111^	0.33 (−0.02–0.68)	0.27 (−0.18 to 0.71)	0.19 (−0.36 to 0.74)
Glutamate^91^	0.29 (0.11–0.46)	0.21 (0.00–0.43)	0.15 (−0.12 to 0.42)
IGF1^84^	0.51 (0.10–0.92)	0.39 (−0.16 to 0.93)	0.23 (−0.45 to 0.91)
IL-6^#^^9^	−0.15 (−0.26 to −0.03)	−0.10 (−0.23 to 0.02)	−0.08 (−0.23 to 0.07)
IL-6^8^	0.35 (0.23–0.48)	0.26 (0.11–0.41)	0.16 (−0.03 to 0.35)
KYNA/3HK^75^	−0.44 (−0.75 to −0.13)	−0.44 (−0.91 to 0.03)	−0.44 (−1.20 to 0.32)
KYNA/QUIN^75^	−0.33 (−0.58 to −0.08)	−0.33 (−0.70 to 0.05)	−0.33 (−0.93 to 0.28)
KYN-ACID^75^	−0.21 (−0.33 to −0.09)	−0.18 (−0.33 to −0.03)	−0.16 (−0.36 to 0.04)
Lipid peroxidation markers^#^^138^	0.44 (0.09–0.79)	0.34 (−0.07 to 0.75)	0.32 (−0.29 to 0.93)
sIL-2 receptor^8^	0.35 (0.09–0.61)	0.25 (−0.08 to 0.59)	0.19 (−0.28 to 0.66)
TNF-alpha^8^	0.15 (0.02–0.28)	0.09 (−0.04 to 0.22)	0.07 (−0.08 to 0.21)
Total cholesterol^94^	−0.11 (−0.17 to −0.05)	−0.09 (−0.16 to −0.02)	−0.05 (−0.14 to 0.04)
*First episode psychosis*			
Cortisol awakening response^119^	−0.43 (−0.72 to −0.14)	−0.40 (−0.81 to 0.01)	−0.40 (−1.06 to 0.26)
*Schizophrenia*			
Adiponectin^#^^166^	−0.20 (−0.32 to −0.08)	−0.17 (−0.32 to −0.01)	−0.14 (−0.34 to 0.07)
Anti-Gliadin IgA^118^	0.20 (0.00–0.40)	0.15 (−0.13 to 0.42)	0.15 (−0.30 to 0.59)
Arachidonic acid^$101^	0.13 (−0.03 to 0.29)	0.06 (−0.11 to 0.23)	0.02 (−0.17 to 0.21)
Cortisol^168^	0.11 (−0.02 to 0.25)	0.03 (−0.10 to 0.17)	0.00 (−0.17 to 0.17)
Folate^105^	−0.18 (−0.29 to −0.07)	−0.16 (−0.29 to −0.02)	−0.13 (−0.32 to 0.07)
MDA^109^	0.50 (0.09–0.91)	0.43 (−0.02 to 0.88)	0.40 (−0.23 to 1.03)
NGF^122^	−0.21 (−0.39 to −0.02)	−0.11 (−0.31 to 0.08)	−0.05 (−0.30 to 0.21)
NMDAR^85^	0.34 (0.07–0.61)	0.34 (−0.06 to 0.74)	0.34 (−0.30 to 0.98)
sIL-2 receptor^7^	0.64 (0.06–1.22)	0.64 (−0.24 to 1.52)	0.64 (−0.78 to 2.05)

Symbols: *Euthymia, **Mania, ^#^Prospective study, ^$^Source: Red blood cell.

*BDNF* brain-derived neurotrophic factor, *IGF* insulin-like growth factor, *IL* interleukine, *INF* interferon, *KynA* kynurenic acid, *Quin* quinolinic acid, *LDL* low-density lipoproteins, *MDA* malondialdehyde, *NMDAR* N-methyl-d-aspartate receptor antibody seropositivity, *NGF* nerve growth factor, *NT* neurotrophin, *QUIN* quinolinic acid, *sIL-2 Receptor* soluble interleukin 2 receptor, *TGF* transforming growth factor, *TNF* tumor necrosis factor, *3HK* 3-hydroxykynurenine.

### Qualitative methodological appraisal of eligible meta-analyses

Qualitative methodological appraisal of eligible meta-analyses through the AMSTAR tool revealed that 49 references were classified as high, 58 as medium, and 3 as low methodological quality, respectively (Table S4). The overall methodological quality of included references was high according to the AMSTAR [(median: 8; IQR = 2 (7–9)] (Table S4).

## Discussion

Our umbrella review provided an up-dated synthesis of the literature of non-genetic peripheral biomarkers for major mental disorders. We included data from 733,316 biomarker measurements. However, in this vast literature only two associations met a priori defined criteria for convincing evidence, whilst 42 meta-analytic estimates met criteria for highly suggestive evidence. This collaborative effort found compelling evidence that overall the literature on non-genetic peripheral biomarkers has a high prevalence of different types of bias. In addition, this umbrella review provides relevant insights for the conduct of further studies to investigate the associations supported by most convincing evidence. It should also be noted that overall the methodological quality of eligible meta-analyses as assessed with the AMSTAR tool was high, which provides further credibility to our quantitative grading of findings.

Associations supported by convincing evidence merit discussion. First, euthymic participants with BD exhibited a high cortisol awakening response relative to controls^87^. This finding indicates that the hypothalamic–pituitary–adrenal (HPA) axis is disrupted in BD on a trait-like basis. This suggests that the HPA axis could be targeted in BD^140^ to improve cognitive function, which may be compromised even during euthymic states^141,142^. In addition, participants with schizophrenia exhibited decreased vitamin B6 (pyridoxal) levels compared to controls^123^. This suggests that individuals with schizophrenia may present aberrations in the one-carbon cycle where pyridoxal is a main metabolic component. An alternative explanation might be the poor nutrition which frequently affects people with schizophrenia^98^. This finding is consistent with a recent systematic review and meta-analysis which provided preliminary evidence that adjunctive pharmacological interventions targeting the one-carbon cycle may improve negative symptoms in schizophrenia (although the clinical significance of this improvement may remain questionable^143^ and aligns with recent evidence showing that adjunctive treatment with B-vitamins may improve symptomatic outcomes in treatment of psychotic disorders^144,145^).

Importantly, only five biomarkers were found to be significantly associated with more than one mental disorder. Also, the highest class of evidence for these biomarkers was II. Moreover, no study applied a methodologically solid approach to assess the trans-diagnostic nature of any biomarker^17^. We found peripheral elevation on the acute phase reactant, CRP, in BD (both during euthymia and mania) as well as in MDD providing evidence that these disorders are at least partly associated with peripheral inflammation. In addition, the s-IL-2R was increased in both MDD and schizophrenia relative to controls. It is noteworthy that IL-2 is a key cytokine involved in the development, survival and function of regulatory T cells (TRegs)^146,147^, and it has been recently proposed that aberrations in “fine tuning” immune-regulatory mechanisms may contribute to the pathophysiology of both MDD and schizophrenia^148,149^. Antibodies against the NMDA-R were increased in BD and schizophrenia. This finding is consistent with the existence of autoantibodies against the GluN1 subunit of this receptor in patients with psychotic manifestations^150,151^. Furthermore, lower serum BDNF levels were observed in participants with MDD and AD relative to controls. This finding is consistent with the “neurotrophic hypothesis” of depression^152^, while parallel lines of evidence suggest that aberrations in BDNF signaling may contribute to neurodegeneration in AD^153^. Finally, lower levels of IGF-1 were observed in bipolar mania and MDD compared to controls. This finding is consistent with the modulatory role of glucose-related signaling including the trophic molecule IGF-1 in hippocampal plasticity^154^. In addition, preclinical evidence suggests that IGF-1 may be involved in the pathophysiology of affective disorders^155,156^.

There is an emerging body of literature investigating the putative role of non-genetic peripheral biomarkers for the prediction of treatment response in major mental disorders. Surprisingly, no such biomarkers met criteria for convincing evidence, while only three biomarkers met criteria for type II evidence. Adiponectin levels in schizophrenia decreased after treatment with second-generation antipsychotics. This is an interesting finding since hypoadiponectinemia has been associated with a wide range metabolic diseases which are common untoward effects of these drugs^157,158^. In addition, IL-6 levels decreased after treatment with antidepressants. These data are consistent with preclinical findings which show that antidepressants have anti-inflammatory properties and may also inhibit M1 microglia polarization^159^. Finally, lipid peroxidation markers increased after antidepressant drug treatment for MDD.

It is worth noting that only 15 meta-analytic estimates had a power >0.80 to detect a small ES. In addition, previous umbrella reviews indicate that the vast majority of peripheral biomarker studies are substantially underpowered^20^. This may undermine the progress and reliability of this particular field and of neuroscience in general through the generation of spurious findings^160^. The “true” ESs of most non-genetic peripheral biomarkers may be expected to be small, similarly to those reported in the genetic literature. Therefore, the design of large, multicenter studies with an open pre-registered protocol, or the creation of Consortia, may be a crucial step to assess the role of peripheral biomarkers in the diagnosis and treatment of major mental disorders within the framework of precision psychiatry^1^, as the model adopted by the Enigma neuroimaging group^161^, or similarly to other large collaborative initiatives^162^. Likewise the creation of biomarker scores using a similar rationale as for the generation of polygenic risk scores may ultimately be a next step in this field.

## Strengths and limitations

It should also be noted that large statistical heterogeneity was verified in most included meta-analytic estimates (81.9%). Although this is considered a relevant indicator of bias in this literature, it may also reflect genuine heterogeneity, which may occur both within and between major diagnostic categories^163^. In addition, methodological differences of individual studies included in the assessed meta-analyses may also contribute to heterogeneity. Those include, for example, the time of sample selection as well as measurement properties of the assays (e.g. intra-assay and inter-assay coefficients of variation). Guidelines to standardize the collection and measurement of peripheral biomarkers in psychiatry have been recently proposed^164^. Furthermore, differences in sample selection across individual studies might have contributed to the observed heterogeneity in some meta-analytic estimates. For example, illness stage and disorders in which mixed presentations are common (e.g., bipolar disorder) might have contributed to heterogeneity across some included meta-analyses. In addition, approaches to subtype major mental disorders according to frameworks such as the NIMH Research Domain Criteria may help to decrease the heterogeneity of this literature in the future through the study of biologically valid and more homogenous phenotypes^13,163,165^.

### Conclusion

This umbrella review of non-genetic peripheral biomarkers for major mental disorders revealed that this literature is fraught with several biases and is underpowered. Nevertheless, two associations supported by convincing evidence and 42 associations supported by highly suggestive evidence were verified. Most associations supported by either convincing or highly suggestive evidence pertained to a single disorder. Future multi-centric studies with a priori publicly available protocols, with an ad-hoc methodology to assess the trans-diagnostic nature of biomarkers^17^, as well as the subtyping of these disorders into more biologically valid phenotypes, and enough statistical power may improve the reliability and reproducibility of this field, which is of relevance for the translation of biological and precision psychiatry into practice.

## Supplementary information

Supplementary Online material

## Data Availability

Computer codes used in the analyses of the data are available after reasonable request to the corresponding author of the current study.
