# Supplementary material for: Evidence-based umbrella review of 162 peripheral biomarkers for major mental disorders
Source: Transl Psychiatry. 2020 May 18;10:152. doi: 10.1038/s41398-020-0835-5 (PMC7235270; doi:10.1038/s41398-020-0835-5)
Supplement: Supplementary file 1 — Supplementary Online material [file 41398_2020_835_MOESM1_ESM.docx]

**Supplementary Online Material**

**PubMed/MEDLINE search string (from inception to February 17^th^, 2019)**

(((((((meta-analysis[Title/Abstract]) OR meta-analytic[Title/Abstract]) OR meta-analysis[Publication Type]) OR metaanaly*[Title/Abstract]) OR meta-analy*[Title/Abstract])) AND ((((((((((((depression[Title/Abstract]) OR unipolar depression[Title/Abstract]) OR bipolar depression[Title/Abstract]) OR mania[Title/Abstract]) OR major depression[Title/Abstract]) OR major depressive disorder[Title/Abstract]) OR schizophrenia[Title/Abstract]) OR psychosis[Title/Abstract]) OR autism[Title/Abstract]) OR autis*[Title/Abstract]) OR Alzheimer's[Title/Abstract]) OR Alzheimer[Title/Abstract])) AND (biomarker* OR "Biomarkers"[Mesh] OR adrenocorticotropic hormone OR cortisol OR ACTH OR dexamethasone suppression test OR CRH suppression test OR brain-derived neurotrophic factor OR BDNF OR vascular endothelial growth factor OR VEGF OR insulin-like growth factor OR glial cell-derived neurotrophic factor OR GDNF OR IGF-1 OR IGF-2 OR oxidative stress OR insulin OR leptin OR insulin resistance OR neuron specific enolase OR cytokine OR chemokine OR adiponectin OR resistin OR ghrelin OR adipokine* OR tumor necrosis factor* OR TNF OR interleukin* OR complement OR complement C3 OR complement C4 OR C-reactive protein OR CRP OR haptoglobin OR Immunoglobulin A OR immunoglobulin* OR IFN OR interferon OR interferon gamma OR interleukin-1 beta OR interleukin-1 OR interleukin-6 OR interleukin-4 OR interleukin-5 OR interleukin-8 OR interleukin-12 OR soluble interleukin-2 receptor OR interleukin-1 receptor antagonist OR superoxide dismutase OR glutathione peroxidase OR malondialdehyde OR angiotensin-converting enzyme OR neopterin OR 8-hydroxy-2-guanosine OR reduced glutathione OR glutathione reductase OR erythrocyte* AND sedimentation rate OR plasma level* OR peripheral level* OR blood level* OR serum level* OR 2,3-dinor-5,6-dihydro-15-F2t-isoprostane OR NGF OR nerve growth factor OR 8-iso-PGF-2alpha OR F2-isoprostanes OR kynurenine OR Kynurenine/tryptophan ratio OR tryptophan catabolite* OR TRYCAT* OR kynurenic acid OR kynurenic acid/kynurenine ratio OR kynurenine OR L-tryptophan OR serine OR amino acid OR monocyte Chemotactic Protein OR CCL2 OR CXCL8 OR IL-* OR NAP OR XCL* OR CX3C* OR CCL* OR scya OR scyb OR GCP OR glial fibrillary acidic protein OR GFAP OR S100 beta OR S-100 beta OR S100-beta OR HPA OR neurotrophic OR NO OR nitric oxide OR nitrosative stress OR reactive nitrogen species OR reactive oxygen species OR GSK-3beta OR Glycogen synthase kinase 3 OR total antioxidant capacity OR paraoxonase OR adenosine OR purine* OR total oxidant status OR total antioxidant status OR enzyme* OR Na+/K+ atpase OR platelet* OR blood mononuclear cell* OR leukocyte* OR leucocyte* OR peripheral OR plasma* OR serum)

**Table S1 Criteria for grading the credibility of evidence**

| **Class of evidence** | **Criteria** |
| --- | --- |
| **Convincing (Class I)** | **Meta-analysis has an estimated power > 0.8 to detect a small effect size (i.e., ES=0.20); not large heterogeneity (i.e., I^2^ < 50.0%); 95% prediction interval not crossing the null; no evidence of excess of significance bias; no evidence of small-study effects; significant associations at *P* < 0.005 per random-effects calculations** |
| **Highly Suggestive (Class II)** | **Meta-analysis has an estimated power > 0.8 to detect a medium effect size (i.e., ES=0.50); significant associations with *P* < 0.005 (random-effects); effect size of the largest study and its 95%CI not including the null** |
| **Suggestive (Class III)** | **Meta-analysis has an estimated power of > 0.80 to detect a medium effect size (i.e., ≥ 0.5); significant associations with *P* < 0.005 (random-effects)** |
| **Suggestive (Class IV)** | **All other significant associations (P < 0.05) per random-effects calculations** |
| **Non-significant** | **All meta-analyses with P ≥ 0.05** |

**Table S2.** Excluded Papers with reasons.

| **Reference** | **Reason for exclusion** |
| --- | --- |
| Ahmed *et al.*, 2015 **[1]** | No control group or no intervention |
| Annweiler *et al.*, 2013 **[2]** | Not BD, MDD, SZ, FEP, AD or ASD |
| Anstey *et al.*, 2017 **[3]** | Not a peripheral biomarker (i.e. urine/blood/saliva) |
| Boggero *et al.*, 2017 **[4]** | Not BD, MDD, SZ, FEP, AD or ASD |
| Capuzzi *et al.*, 2017 **[5]** | Meta-analysis with a largest number of datasets available |
| Carvalho *et al.*, 2015 **[6]** | Meta-analysis with a largest number of datasets available |
| Chan *et al.*, 2015 **[7]** | Not a meta-analysis |
| Chan *et al.*, 2016 **[8]** | No effect size reported |
| Chaumette *et al.*, 2016 **[9]** | Not BD, MDD, SZ, FEP, AD or ASD |
| Cooper *et al.*, 2015 **[10]** | Not a peripheral biomarker (i.e. urine/blood/saliva) |
| Czapski *et al.*, 2012 **[11]** | Not a meta-analysis |
| Dargel *et al.*, 2015 **[12]** | Meta-analysis with a largest number of datasets available |
| Deng *et al.*, 2016 **[13]** | Not a meta-analysis |
| Dong *et al.*, 2018 **[14]** | Meta-analysis with a largest number of datasets available |
| Dowlati *et al.*, 2010 **[15]** | Meta-analysis with a largest number of datasets available |
| Ellis *et al.*, 1994 **[16]** | Not a peripheral biomarker (i.e. urine/blood/saliva) |
| Ezeoke *et al.*, 2013 **[17]** | Not a meta-analysis |
| Fernandes *et al.*, 2011 **[18]** | Meta-analysis with a largest number of datasets available |
| Fischer *et al.*, 2015 **[19]** | Meeting abstract |
| Fischer *et al.*, 2016 **[20]** | Baseline cortisol predicting intervention efficacy |
| Fraguas *et al.*, 2017**[21]** | Meta-analysis with a largest number of datasets available |
| Gowda *et al.*, 2015 **[22]** | Not a peripheral biomarker (i.e. urine/blood/saliva) |
| Gsell *et al.*, 1997 **[23]** | Not a peripheral biomarker (i.e. urine/blood/saliva) |
| Gutierrez-Fernandez *et al.*, 2015 **[24]** | Not a peripheral biomarker (i.e. urine/blood/saliva) |
| Ju *et al.*, 2013 **[25]** | Not BD, MDD, SZ, FEP, AD or ASD |
| Kloiber *et al.*, 2013 **[26]** | Not a meta-analysis |
| Lin *et al.*, 2009 **[27]** | Meta-analysis with a largest number of datasets available |
| Martinez-Cengotitabengoa *et al.*, 2016 **[28]** | Not a meta-analysis |
| McGuinness *et al.*, 2010 **[29]** | Not a peripheral biomarker (i.e. urine/blood/saliva) |
| Miller *et al.*, 2011 **[30]** | Not BD, MDD, SZ, FEP, AD or ASD |
| Miller *et al.*, 2014 **[31]** | Not BD, MDD, SZ, FEP, AD or ASD |
| Noonan *et al.*, 2013 **[32]** | Comparing stroke patients with and without depression |
| Pascoe *et al.*, 2017**[33]** | Not BD, MDD, SZ, FEP, AD or ASD |
| Perry *et al.*, 2016 **[34]** | Not BD, MDD, SZ, FEP, AD or ASD |
| Ritchie *et al.*, 2014 **[35]** | Not a meta-analysis |
| Rocha *et al.* **[36]** | Meta-analysis with a largest number of datasets available |
| Rosenblat *et al.*, 2016 **[37]** | Not a meta-analysis |
| Ruhe *et al.*, 2007 **[38]** | Not a peripheral biomarker (i.e. urine/blood/saliva) |
| Sarris *et al.*, 2016 **[39]** | Not a peripheral biomarker (i.e. urine/blood/saliva) |
| Schroeter *et al.*, 2009 **[40]** | Meta-analysis with a largest number of datasets available |
| Schumberg *et al.*, 2016 **[41]** | Meta-analysis with a largest number of datasets available |
| Squitti *et al.*, 2014 **[42]** | Not a peripheral biomarker (i.e. urine/blood/saliva) |
| Tuckwell *et al.*, 1996 **[43]** | Not a peripheral biomarker (i.e. urine/blood/saliva) |
| Upthegrove *et al.*, 2014 **[44]** | Not BD, MDD, SZ, FEP, AD or ASD |
| Ventriglia *et al.*, 2012 **[45]** | Meta-analysis with a largest number of datasets available |
| Wang *et al.*, 2014 **[46]** | Meta-analysis with a largest number of datasets available |
| Wang *et al.*, 2016 **[47]** | No control group or no intervention |
| Wiedlocha *et al.*, 2017 **[48]** | Meta-analysis with a largest number of datasets available |
| Williams *et al.*, 2009 **[49]** | No control group or no intervention |
| Yoshida *et al.*, 2012 **[50]** | Not a meta-analysis |
| Yuan *et al.*, 2017 **[51]** | Not a peripheral biomarker (i.e. urine/blood/saliva) |
| Zhang *et al.*, 2017 **[52]** | Not a peripheral biomarker (i.e. urine/blood/saliva) |
| Zhou *et al.,*2017 **[53]** | Meta-analysis with a largest number of datasets available |
| Ellul *et al.*, 2016 **[54]** | Meta-analysis with a largest number of datasets available |
| Eyre *et al.*, 2016 **[55]** | Meta-analysis with a largest number of datasets available |
| Jiang *et al.*, 2014 **[56]** | Meta-analysis with a largest number of datasets available |
| Hiles *et al.*, 2012 **[57]** | Meta-analysis with a largest number of datasets available |
| Liu *et al.*, 2012 **[58]** | Meta-analysis with a largest number of datasets available |
| Hannestad *et al.*, 2011 [59] | Meta-analysis with a largest number of datasets available |
| Munkholm *et al.*, 2013 **[60]** | Meta-analysis with a largest number of datasets available |
| Modabbernia *et al.*, 2013 **[61]** | Meta-analysis with a largest number of datasets available |

**Abbreviations:** AD, Alzheimer Disease; ASD, autism spectrum disorder; FEP, First Episode Psychiatry; BD, Bipolar Disorder; SZ, Schizophrenia; MDD, Major Depressive Disorder.

| Table S3. Peripheral biomarkers derived from eligible between-group meta-analyses. | | | | | | | | | | | | | |  | | |  | | |  | |  |  |
| --- | --- | --- | --- | --- | --- | --- | --- | --- | --- | --- | --- | --- | --- | --- | --- | --- | --- | --- | --- | --- | --- | --- | --- |
| Reference | **Biomarker** | **N cases/ controls** | **N** | **Effect size metric** | **Random effects summary effect size (95% CI)** | **P (Random effects)** | | | **95% PI** | | **I^2^** | | **Small-study effects/ excess significance bias** | | | **Power criteria**  **Small/ Medium ES** | | | **Level of evidence^*^** | **AMSTAR** | | | |
| Acute Depression |  |  |  |  |  | |  |  | |  | |  | | |  | | |  | | |  | |  |
| Goldsmith *et al*., 2016[62] | IL-10 | 112/103 | 4 | Hedges’ g | 1.08 (-0.01 – 2.17) | | 0.053 | -2.72 – 4.88 | | 92.0 | | No/No | | | No/No | | | NS | | | 10 | |  |
| Goldsmith *et al*., 2016[62] | IL-12 | 117/172 | 4 | Hedges’ g | 1.35 (0.28 – 2.41) | | 0.013 | -2.39 – 5.08 | | 93.3 | | No/No | | | No/No | | | NS | | | 10 | |  |
| Goldsmith *et al*., 2016[62] | IL-1β | 116/112 | 4 | Hedges’ g | -0.47 (-1.66 – 0.72) | | 0.436 | -4.67 – 3.72 | | 94.1 | | No/No | | | No/No | | | NS | | | 10 | |  |
| Goldsmith *et al*., 2016[62] | IL-2 | 84/79 | 3 | Hedges’ g | 0.39 (-2.62 – 3.41) | | 0.798 | -12.74 – 13.53 | | 98.2 | | No/No | | | No/No | | | NS | | | 10 | |  |
| Goldsmith *et al*., 2016[62] | IL-6 | 306/216 | 10 | Hedges’ g | 0.97 (0.31 – 1.62) | | 0.004 | -1.39 – 3.32 | | 90.6 | | No/No | | | No/No | | | IV | | | 10 | |  |
| Goldsmith *et al*., 2016[62] | sIL-2R | 247/170 | 6 | Hedges’ g | 0.51 (0.19 – 0.84) | | 0.002 | -0.34 – 1.37 | | 54.7 | | No/No | | | No/Yes | | | III | | | 10 | |  |
| Goldsmith *et al*., 2016[62] | sIL-6R | 145/68 | 3 | Hedges’ g | 0.67 (0.32 – 1.03) | | 0.000 | -0.36 – 1.71 | | 25.3 | | No/No | | | No/No | | | IV | | | 10 | |  |
| Goldsmith *et al*., 2016[62] | TNF-Alpha | 296/281 | 9 | Hedges’ g | 0.80 (0.04 – 1.56) | | 0.039 | -1.92 – 3.52 | | 94.1 | | Yes/No | | | No/No | | | NS | | | 10 | |  |
| Chronic Depression |  |  |  |  |  | |  |  | |  | |  | | |  | | |  | | |  | |  |
| Goldsmith *et al*., 2016[62] | IFN-Gamma | 195/226 | 4 | Hedges’ g | -0.64 (-1.93 – 0.65) | | 0.330 | -5.25 – 3.97 | | 97.2 | | No/No | | | No/No | | | NS | | | 10 | |  |
| Goldsmith *et al*., 2016[62] | IL-10 | 138/172 | 5 | Hedges’ g | -0.54 (-1.28 – 0.20) | | 0.151 | -2.92 – 1.84 | | 88.5 | | No/No | | | No/No | | | NS | | | 10 | |  |
| Goldsmith *et al*., 2016[62] | IL-1β | 138/190 | 4 | Hedges’ g | -0.38 (-1.57 – 0.80) | | 0.528 | -4.61 – 3.84 | | 95.9 | | No/Yes | | | No/No | | | NS | | | 10 | |  |
| Goldsmith *et al*., 2016[62] | IL-6 | 205/235 | 8 | Hedges’ g | 0.53 (0.10 – 0.96) | | 0.016 | -0.84 – 1.89 | | 77.7 | | Yes/Yes | | | No/No | | | NS | | | 10 | |  |
| Goldsmith *et al*., 2016[62] | IL-8 | 126/161 | 4 | Hedges’ g | 0.12 (-0.43 – 0.68) | | 0.659 | -1.70 – 1.95 | | 80.0 | | No/Yes | | | No/No | | | NS | | | 10 | |  |
| Goldsmith *et al*., 2016[62] | sIL-2R | 117/190 | 4 | Hedges’ g | -0.03 (-0.52 – 0.46) | | 0.895 | -1.59 – 1.53 | | 72.3 | | No/Yes | | | No/No | | | NS | | | 10 | |  |
| Goldsmith *et al*., 2016[62] | sIL-6R | 66/66 | 3 | Hedges’ g | -0.09 (-0.43 – 0.26) | | 0.625 | -0.84 – 0.67 | | 0.0 | | No/No | | | No/Yes | | | NS | | | 10 | |  |
| Goldsmith *et al*., 2016[62] | TNF-Alpha | 395/518 | 12 | Hedges’ g | 0.19 (-0.19 – 0.57) | | 0.316 | -1.22 – 1.61 | | 86.1 | | No/Yes | | | No/No | | | NS | | | 10 | |  |
| Depression |  |  |  |  |  | |  |  | |  | |  | | |  | | |  | | |  | |  |
| Ogyu *et al.*, 2018 [63] | 3HK | 411/439 | 10 | SMD | 0.05 (-0.08 – 0.19) | | 0.420 | -0.10 – 0.21 | | 0.0 | | No/No | | | Yes/Yes | | | NS | | | 9 | |  |
| Nascimento *et al.*, 2015 [64] | A BETA 42 | 212/1022 | 5 | SMD | -0.44 (-1.00 – 0.11) | | 0.117 | -2.28 – 1.40 | | 90.7 | | No/No | | | No/No | | | NS | | | 7 | |  |
| Cao *et al.*, 2018 [65] | Adiponectin | 1909/6153 | 16 | SMD | -0.24 (-0.47 – -0.02) | | 0.033 | -1.15 – 0.66 | | 91.8 | | No/No | | | No/Yes | | | NS | | | 9 | |  |
| Lin *et al.*, 2010 [66] | Arachidonic Acid | 638/2656 | 13 | Hedges’g | 0.01 (-0.16 – 0.17) | | 0.934 | -0.46 – 0.48 | | 54.0 | | No/Yes | | | No/Yes | | | NS | | | 5 | |  |
| Mokhtari *et al.*, 2013[67] | Area Under Curve Scores for Cortisol Response To DEX/CRH | 208/204 | 8 | Hedges’g | 1.34 (0.70 – 1.97) | | 0.000 | -0.79 – 3.47 | | 87.1 | | No/No | | | No/No | | | IV | | | 2 | |  |
| Nascimento *et al.*, 2015 [64] | AΒ40:AΒ42 (PLASMA) | 496/918 | 4 | SMD | 1.10 (0.24 – 1.96) | | 0.012 | -1.97 – 4.17 | | 96.8 | | No/No | | | No/No | | | NS | | | 7 | |  |
| Petridou *et al.*, 2016 [68] | B12 Vitamin | 3153/3155 | 9 | SMD | 0.06 (-0.01 – 0.12) | | 0.095 | -0.10 – 0.21 | | 37.9 | | No/No | | | Yes/Yes | | | NS | | | 7 | |  |
| Molendijk *et al.*, 2014 [69] | BDNF | 2578/3961 | 35 | Hedges’g | -0.61 (-0.80 – -0.42) | | 0.000 | -1.67 – 0.45 | | 87.7 | | No/No | | | No/Yes | | | II | | | 10 | |  |
| Kohler et al., 2017 [70] | CCL-2 | 285/287 | 8 | Hedges’g | 1.72 (0.64 – 2.79) | | 0.002 | -2.04 – 5.47 | | 96.3 | | Yes/No | | | No/No | | | IV | | | 11 | |  |
| Kohler et al., 2017 [70] | CCL-3 | 110/98 | 3 | Hedges’g | 1.97 (-0.23 – 4.18) | | 0.079 | -7.59 – 11.54 | | 97.5 | | No/No | | | No/No | | | NS | | | 11 | |  |
| Ni *et al*., [71] | Copper | 1167/765 | 16 | SMD | 0.91 (0.34 – 1.48) | | 0.002 | -1.59 – 3.42 | | 96.2 | | Yes/No | | | No/No | | | IV | | | 8 | |  |
| Ciufolini *et al.*, 2014 [72] | Cortisol Delta to Social Stress Test | 298/503 | 9 | SMD | 0.20 (-0.33 – 0.72) | | 0.461 | -1.61 – 2.01 | | 89.7 | | No/Yes | | | No/No | | | NS | | | 4 | |  |
| Ciufolini *et al.*, 2014 [72] | Cortisol Level in Antecipation To Social Stress Test | 298/503 | 9 | SMD | 0.31 (-0.23 – 0.84) | | 0.263 | -1.54 – 2.16 | | 90.1 | | No/No | | | No/No | | | NS | | | 4 | |  |
| Ciufolini *et al.*, 2014 [72] | Cortisol Peak Response to Social Stress Test | 298/503 | 9 | SMD | 0.10 (-0.27 – 0.48) | | 0.585 | -1.11 – 1.32 | | 80.1 | | No/No | | | No/No | | | NS | | | 4 | |  |
| Zorn *et al.*, 2016[73] | Cortisol Reactivity to a Laboratory Psychosocial Stressor | 1041/1465 | 55 | SMD | -0.12 (-0.23 – 0.00) | | 0.052 | -0.68 – 0.45 | | 41.3 | | No/Yes | | | Yes/Yes | | | NS | | | 4 | |  |
| Haapakoski *et al.*, 2015 [74] | CRP | 7525/6209 | 20 | SMD | 0.46 (0.39 – 0.53) | | 0.000 | 0.39 – 0.54 | | 0.0 | | No/No | | | Yes/Yes | | | II | | | 8 | |  |
| Zhu *et al.*, 2015 [75] | DHEAS | 469/3916 | 10 | SMD | 0.78 (0.16 – 1.40) | | 0.014 | -1.51 – 3.07 | | 95.3 | | No/No | | | No/No | | | NS | | | 7 | |  |
| Lin *et al.*, 2010 [66] | DHA | 648/2670 | 14 | Hedges’ g | -0.35 (-0.55 – -0.16) | | 0.000 | -0.98 – 0.28 | | 67.8 | | No/Yes | | | No/Yes | | | III | | | 5 | |  |
| Lin *et al.*, 2010 [66] | EPA | 648/2670 | 14 | Hedges’ g | -0.20 (-0.34 – -0.05) | | 0.008 | -0.57 – 0.18 | | 41.5 | | No/No | | | No/Yes | | | NS | | | 5 | |  |
| Wu *et al.*, 2016 [76] | Fibroblast Growth Factor-2 | 99/80 | 4 | SMD | 0.43 (0.13 – 0.73) | | 0.005 | -0.05 – 0.91 | | 0.0 | | No/No | | | No/Yes | | | II | | | 5 | |  |
| Bender *et al.*, 2017 [77] | Folate | 2766/10134 | 29 | SMD | -0.35 (-0.49 – -0.20) | | 0.000 | -1.07 – 0.38 | | 88.2 | | No/No | | | No/Yes | | | III | | | 7 | |  |
| Romeo *et al.*, 2018 [78] | Gaba | 241/199 | 6 | SMD | -1.40 (-2.04 – -0.76) | | 0.000 | -3.42 – 0.62 | | 87.1 | | No/No | | | No/No | | | IV | | | 6 | |  |
| Lin *et al.*, 2015 [79] | Glial Cell Line-Derived Neurotrophic Factor | 556/491 | 10 | Hedges’ g | -0.73 (-1.08 – -0.37) | | 0.000 | -1.98 – 0.53 | | 84.5 | | No/No | | | No/No | | | IV | | | 7 | |  |
| Inoshita *et al.*, 2018 [80] | Glutamate | 529/590 | 12 | SMD | 0.54 (0.29 – 0.79) | | 0.000 | -0.27 – 1.35 | | 69.8 | | No/No | | | No/Yes | | | II | | | 5 | |  |
| Kohler et al., 2017 [70] | IFN-Gamma(4) | 700/770 | 17 | Hedges’g | -0.48 (-0.94 – -0.02) | | 0.043 | -2.51 – 1.56 | | 94.0 | | No/No | | | No/No | | | NS | | | 11 | |  |
| Tu *et al.*, 2016 [81] | IGF1 | 180/197 | 6 | Hedges’g | 0.64 (0.41 – 0.87) | | 0.000 | 0.24 – 1.04 | | 12.1 | | No/No | | | No/Yes | | | II | | | 6 | |  |
| Kohler et al., 2017 [70] | IL-10 | 608/675 | 17 | Hedges’g | 0.38 (0.01 – 0.74) | | 0.045 | -1.20 – 1.95 | | 89.2 | | No/No | | | No/No | | | NS | | | 11 | |  |
| Kohler et al., 2017 [70] | IL-12 | 135/301 | 4 | Hedges’g | 1.23 (0.28 – 2.18) | | 0.012 | -2.10 – 4.56 | | 92.9 | | No/No | | | No/No | | | NS | | | 11 | |  |
| Kohler et al., 2017 [70] | IL-13 | 243/373 | 6 | Hedges’g | 1.84 (0.81 – 2.86) | | 0.000 | -1.62 – 5.29 | | 96.0 | | Yes/No | | | No/No | | | IV | | | 11 | |  |
| Kohler et al., 2017 [70] | IL-17 | 85/106 | 3 | Hedges’g | -0.12 (-0.54 – 0.29) | | 0.569 | -1.58 – 1.34 | | 51.6 | | No/Yes | | | No/No | | | NS | | | 11 | |  |
| Kohler et al., 2017 [70] | IL-18 | 135/143 | 5 | Hedges’g | 1.72 (0.38 – 3.06) | | 0.012 | -2.83 – 6.27 | | 95.3 | | No/No | | | No/No | | | NS | | | 11 | |  |
| Kohler et al., 2017 [70] | IL-1RA | 148/110 | 4 | Hedges’g | 0.45 (0.08 – 0.81) | | 0.016 | -0.59 – 1.49 | | 51.6 | | No/No | | | No/No | | | NS | | | 11 | |  |
| Kohler et al., 2017 [70] | IL-1Β | 779/727 | 22 | Hedges’g | 0.03 (-0.29 – 0.35) | | 0.847 | -1.52 – 1.58 | | 89.3 | | No/Yes | | | No/Yes | | | NS | | | 11 | |  |
| Kohler et al., 2017 [70] | IL-2 | 357/476 | 10 | Hedges’g | -0.11 (-0.90 – 0.68) | | 0.789 | -3.05 – 2.84 | | 95.8 | | No/Yes | | | No/No | | | NS | | | 11 | |  |
| Kohler et al., 2017 [70] | IL-4 | 350/450 | 10 | Hedges’g | -0.53 (-1.07 – 0.01) | | 0.053 | -2.47 – 1.41 | | 91.0 | | No/No | | | No/No | | | NS | | | 11 | |  |
| Kohler et al., 2017 [70] | IL-5 | 198/322 | 4 | Hedges’g | 0.40 (-0.07 – 0.86) | | 0.097 | -1.17 – 1.96 | | 82.1 | | No/No | | | No/No | | | NS | | | 11 | |  |
| Kohler et al., 2017 [70] | IL-6 | 1526/1124 | 40 | Hedges’g | 0.63 (0.48 – 0.77) | | 0.000 | -0.13 – 1.38 | | 66.5 | | No/No | | | No/Yes | | | II | | | 11 | |  |
| Kohler et al., 2017 [70] | IL-8 | 306/217 | 7 | Hedges’g | 0.03 (-0.35 – 0.41) | | 0.869 | -1.15 – 1.22 | | 76.9 | | No/Yes | | | No/No | | | NS | | | 11 | |  |
| Ogyu *et al.*, 2018 [63] | KYN | 1780/1216 | 18 | SMD | -0.17 (-0.29 – -0.05) | | 0.005 | -0.53 – 0.19 | | 42.5 | | No/Yes | | | Yes/Yes | | | NS | | | 9 | |  |
| Ogyu *et al.*, 2018 [63] | KYNA / 3HK | 278/287 | 5 | SMD | -0.47 (-0.64 – -0.30) | | 0.000 | -0.71 – -0.23 | | 0.0 | | No/No | | | No/Yes | | | II | | | 9 | |  |
| Ogyu *et al.*, 2018 [63] | KYNA / QUIN | 278/287 | 5 | SMD | -0.50 (-0.77 – -0.22) | | 0.000 | -1.28 – 0.28 | | 60.6 | | No/No | | | No/Yes | | | II | | | 9 | |  |
| Ogyu *et al.*, 2018 [63] | KYN-ACID | 691/801 | 17 | SMD | -0.36 (-0.52 – -0.19) | | 0.000 | -0.93 – 0.21 | | 56.0 | | No/No | | | No/Yes | | | II | | | 9 | |  |
| Persons et al., 2016 [82] | LDL Continuous | 4987/11931 | 42 | SMD | -0.09 (-0.25 – 0.08) | | 0.297 | -1.12 – 0.95 | | 94.0 | | No/No | | | No/Yes | | | NS | | | 4 | |  |
| Cao *et al.*, 2018 [65] | Leptin | 3780/6331 | 23 | SMD | 0.13 (-0.06 – 0.32) | | 0.173 | -0.71 – 0.97 | | 91.9 | | No/Yes | | | No/Yes | | | NS | | | 9 | |  |
| Mazereeuw *et al.*, 2015 [83] | Lipid Peroxidation Markers | 857/782 | 18 | SMD | 0.83 (0.56 – 1.09) | | 0.000 | -0.29 – 1.95 | | 84.0 | | Yes/No | | | No/Yes | | | III | | | 8 | |  |
| Ogawa *et al*., 2014 [84] | L-Tryptophan | 744/877 | 25 | Hedges’ g | -0.63 (-0.82 – -0.44) | | 0.000 | -1.41 – 0.15 | | 63.8 | | No/No | | | No/Yes | | | III | | | 7 | |  |
| You *et al.*, 2018 [85] | Magnesium | 1026/977 | 15 | SMD | -0.51 (-1.60 – 0.58) | | 0.355 | -5.24 – 4.21 | | 98.7 | | No/No | | | No/No | | | NS | | | 8 | |  |
| Chen *et al.*, 2015 [86] | NGF | 506/555 | 11 | Hedges’ g | -0.28 (-0.55 – -0.01) | | 0.046 | -1.26 – 0.70 | | 82.8 | | No/Yes | | | No/Yes | | | NS | | | 5 | |  |
| Pearlman, 2014 [87] | N-Methyl-D-Aspartate Receptor Antibody Seropositivity | 105/690 | 3 | SMD | 0.27 (0.06 – 0.47) | | 0.011 | -0.19 – 0.72 | | 0.0 | | No/No | | | No/Yes | | | NS | | | 7 | |  |
| Lin *et al.*, 2010 [66] | Omega-3 | 602/2620 | 12 | Hedges’ g | -0.53 (-0.78 – -0.29) | | 0.000 | -1.34 – 0.28 | | 78.2 | | No/No | | | No/Yes | | | III | | | 5 | |  |
| Lin *et al.*, 2010 [66] | Omega-6 | 516/2570 | 10 | Hedges’ g | 0.01 (-0.17 – 0.19) | | 0.932 | -0.48 – 0.50 | | 55.9 | | No/No | | | No/Yes | | | NS | | | 5 | |  |
| Mazza *et al.*, 2018 [88] | Platelet/Lymphocyte Ratio | 239/314 | 4 | SMD | 0.67 (0.07 – 1.27) | | 0.028 | -1.40 – 2.74 | | 89.9 | | No/No | | | No/No | | | NS | | | 7 | |  |
| Ogyu *et al.*, 2018 [63] | Quin | 477/465 | 10 | SMD | 0.22 (-0.11 – 0.56) | | 0.187 | -0.94 – 1.39 | | 83.9 | | No/Yes | | | No/Yes | | | NS | | | 9 | |  |
| Carvalho *et al.*, 2014 [89] | Resistin | 148/150 | 3 | Hedges’ g | -0.24 (-0.49 – 0.00) | | 0.051 | -0.88 – 0.39 | | 12.7 | | No/No | | | No/Yes | | | NS | | | 8 | |  |
| Kohler et al., 2017 [70] | sIL-2 Receptor | 489/391 | 10 | Hedges’g | 0.74 (0.42 – 1.05) | | 0.000 | -0.32 – 1.79 | | 77.5 | | No/No | | | No/Yes | | | II | | | 11 | |  |
| Kohler et al., 2017 [70] | sIL-6 Receptor | 344/256 | 7 | Hedges’g | 0.33 (-0.01 – 0.67) | | 0.055 | -0.68 – 1.34 | | 71.3 | | No/No | | | No/Yes | | | NS | | | 11 | |  |
| Kohler et al., 2017 [70] | sTNF Receptor 2 | 94/101 | 3 | Hedges’g | 1.17 (0.41 – 1.94) | | 0.003 | -1.96 – 4.30 | | 83.2 | | No/No | | | No/No | | | IV | | | 11 | |  |
| Kohler et al., 2017 [70] | TGF-Beta 1 | 110/68 | 3 | Hedges’g | -1.48 (-4.76 – 1.80) | | 0.376 | -15.71 – 12.75 | | 97.7 | | No/No | | | No/No | | | NS | | | 11 | |  |
| Kohler et al., 2017 [70] | TNF-Alpha | 1620/1457 | 42 | Hedges’g | 0.68 (0.43 – 0.92) | | 0.000 | -0.88 – 2.23 | | 90.0 | | Yes/No | | | No/Yes | | | II | | | 11 | |  |
| Shin *et al.*, 2008[90] | Total Cholesterol | 46010/46265 | 36 | Cohen’s d | -0.25 (-0.37 – -0.13) | | 0.000 | -0.87 – 0.37 | | 92.8 | | No/No | | | Yes/Yes | | | II | | | 5 | |  |
| Bartoli *et al.*, 2018 [91] | Uric Acid | 842/3964 | 13 | SMD | -0.30 (-0.50 – -0.10) | | 0.003 | -1.02 – 0.41 | | 76.5 | | No/No | | | No/Yes | | | III | | | 9 | |  |
| Rutigliano *et al.*, 2016 [92] | Vasopressin | 265/158 | 6 | Hedges’g | 0.34 (-0.08 – 0.76) | | 0.111 | -0.93 – 1.61 | | 73.8 | | No/No | | | No/No | | | NS | | | 11 | |  |
| Tseng *et al.*, 2015 [93] | VEGF | 872/882 | 16 | Hedges’g | 0.44 (0.24 – 0.63) | | 0.000 | -0.26 – 1.13 | | 68.9 | | No/Yes | | | No/Yes | | | III | | | 7 | |  |
| Anglin *et al.*, 2013 [94] | Vitamin D | 9837/12481 | 9 | SMD | 0.05 (0.00 – 0.10) | | 0.065 | -0.08 – 0.18 | | 55.3 | | No/No | | | Yes/Yes | | | NS | | | 10 | |  |
| Swardfager *et al.*, 2013 [95] | Zinc | 1643/804 | 18 | WMD | -0.74 (-1.01 – -0.47) | | 0.000 | -1.87 – 0.39 | | 85.0 | | No/No | | | No/Yes | | | III | | | 10 | |  |
| Bipolar |  |  |  |  |  | |  |  | |  | |  | | |  | | |  | | |  | |  |
| Belvederi Murri *et al.*, 2016 [96] | ACTH | 85/68 | 4 | Hedges’g | 0.41 (-0.43 – 1.26) | | 0.339 | -2.22 – 3.05 | | 69.8 | | No/No | | | No/No | | | NS | | | 8 | |  |
| Belvederi Murri *et al.*, 2016 [96] | Basal Cortisol Afternoon Sample | 153/224 | 7 | Hedges’g | 0.30 (0.08 – 0.53) | | 0.008 | 0.02 – 0.58 | | 0.0 | | Yes/No | | | No/Yes | | | NS | | | 8 | |  |
| Belvederi Murri *et al.*, 2016 [96] | Basal Cortisol Awakening | 605/1521 | 10 | Hedges’g | 0.25 (0.15 – 0.35) | | 0.000 | 0.13 – 0.37 | | 0.0 | | No/No | | | Yes/Yes | | | I | | | 8 | |  |
| Belvederi Murri *et al.*, 2016 [96] | Basal Cortisol Continuous | 256/319 | 7 | Hedges’g | 0.31 (0.00 – 0.63) | | 0.053 | -0.57 – 1.20 | | 61.7 | | No/No | | | No/Yes | | | NS | | | 8 | |  |
| Belvederi Murri *et al.*, 2016 [96] | Basal Cortisol Morning Sample | 552/1076 | 23 | Hedges’g | 0.37 (0.22 – 0.53) | | 0.000 | -0.04 – 0.79 | | 25.8 | | No/No | | | No/Yes | | | III | | | 8 | |  |
| Belvederi Murri *et al.*, 2016 [96] | Basal Cortisol Night Sample | 280/545 | 8 | Hedges’g | 0.20 (-0.08 – 0.49) | | 0.164 | -0.55 – 0.96 | | 55.8 | | No/No | | | No/Yes | | | NS | | | 8 | |  |
| Looney *et al.*, 1997 [97] | NA,K-ATPASE ACTIVITY | 234/296 | 11 | SMD | 0.15 (-0.25 – 0.56) | | 0.462 | -1.21 – 1.51 | | 76.9 | | No/Yes | | | No/No | | | NS | | | 1 | |  |
| Rao *et al.*, 2017 [98] | NGF | 299/275 | 5 | SMD | 0.13 (-0.03 – 0.29) | | 0.104 | -0.09 – 0.36 | | 0.0 | | No/No | | | No/Yes | | | NS | | | 9 | |  |
| Pearlman, 2014 [87] | N-Methyl-D-Aspartate Receptor Antibody Seropositivity | 240/1035 | 5 | SMD | 0.56 (0.41 – 0.70) | | 0.000 | 0.34 – 0.78 | | 3.1 | | No/No | | | No/Yes | | | II | | | 7 | |  |
| Tseng *et al.*, 2016 [99] | Neurotrophin-3 | 260/718 | 14 | Hedges’g | 0.38 (0.12 – 0.63) | | 0.004 | -0.57 – 1.32 | | 74.9 | | No/Yes | | | No/Yes | | | II | | | 7 | |  |
| Tseng *et al.*, 2016 [99] | Neurotrophin-4/5 | 268/218 | 7 | Hedges’g | 0.29 (-0.10 – 0.68) | | 0.142 | -0.92 – 1.50 | | 76.9 | | No/Yes | | | No/No | | | NS | | | 7 | |  |
| Rutigliano *et al.*, 2016 [92] | Oxytocin | 153/122 | 3 | SMD | 0.06 (-0.85 – 0.96) | | 0.903 | -3.74 – 3.85 | | 90.4 | | No/Yes | | | No/No | | | NS | | | 11 | |  |
| Bartoli *et al.*, 2016 [100] | Uric Acid | 619/508 | 9 | SMD | 0.65 (0.33 – 0.96) | | 0.000 | -0.42 – 1.72 | | 82.6 | | No/No | | | No/Yes | | | II | | | 6 | |  |
| Bipolar Disorder (Depression) |  |  |  |  |  | |  |  | |  | |  | | |  | | |  | | |  | |  |
| Fernandes *et al.*, 2015 [101] | BDNF | 365/744 | 16 | Hedges’g | -0.87 (-1.27 – -0.47) | | 0.000 | -2.48 – 0.74 | | 86.2 | | No/No | | | No/No | | | IV | | | 8 | |  |
| Fernandes *et al.*, 2016 [102] | CRP | 441/920 | 11 | Hedges’g | 0.67 (0.23 – 1.11) | | 0.003 | -0.95 – 2.28 | | 90.9 | | No/No | | | No/No | | | IV | | | 7 | |  |
| Goldsmith *et al*., 2016[62] | IL-6 | 102/344 | 3 | Hedges’g | 0.10 (-0.38 – 0.57) | | 0.691 | -1.75 – 1.94 | | 71.2 | | No/Yes | | | No/No | | | NS | | | 10 | |  |
| Fernandes *et al.*, 2016 [103] | Leptin | 227/222 | 5 | Hedges’g | 0.18 (-0.42 – 0.77) | | 0.566 | -1.78 – 2.13 | | 87.7 | | No/Yes | | | No/No | | | NS | | | 7 | |  |
| Looney *et al.*, 1997 [97] | NA,K-ATPASE ACTIVITY | 55/99 | 5 | SMD | -0.65 (-1.36 – 0.06) | | 0.075 | -2.70 – 1.41 | | 72.4 | | No/No | | | No/No | | | NS | | | 1 | |  |
| Bipolar Disorder (Euthymia) |  |  |  |  |  | |  |  | |  | |  | | |  | | |  | | |  | |  |
| Fernandes *et al.*, 2016 [102] | CRP | 783/79693 | 17 | Hedges’g | 0.65 (0.40 – 0.90) | | 0.000 | -0.39 – 1.69 | | 85.2 | | No/No | | | No/Yes | | | II | | | 7 | |  |
| Salagre *et al.*, 2017 [104] | Homocysteine | 336/547 | 8 | SMD | 0.30 (0.11 – 0.48) | | 0.002 | -0.18 – 0.77 | | 43.7 | | No/No | | | No/Yes | | | III | | | 9 | |  |
| Goldsmith *et al*., 2016[62] | IFN-GAMMA | 91/122 | 4 | Hedges’g | 0.03 (-0.26 – 0.32) | | 0.836 | -0.51 – 0.57 | | 6.2 | | No/No | | | No/Yes | | | NS | | | 10 | |  |
| Goldsmith *et al*., 2016[62] | IL-10 | 166/273 | 8 | Hedges’g | 0.43 (0.01 – 0.85) | | 0.044 | -0.89 – 1.76 | | 74.8 | | Yes/No | | | No/No | | | NS | | | 10 | |  |
| Goldsmith *et al*., 2016[62] | IL-2 | 46/44 | 3 | Hedges’g | 0.19 (-0.31 – 0.69) | | 0.461 | -1.33 – 1.71 | | 30.3 | | No/No | | | No/No | | | NS | | | 10 | |  |
| Goldsmith *et al*., 2016[62] | IL-4 | 60/69 | 4 | Hedges’g | 1.19 (-0.63 – 3.01) | | 0.200 | -5.25 – 7.63 | | 94.9 | | No/No | | | No/No | | | NS | | | 10 | |  |
| Goldsmith *et al*., 2016[62] | IL-6 | 213/540 | 7 | Hedges’g | 0.38 (-0.01 – 0.77) | | 0.054 | -0.83 – 1.59 | | 78.2 | | No/No | | | No/No | | | NS | | | 10 | |  |
| Fernandes *et al.*, 2016 [103] | Leptin | 300/288 | 7 | Hedges’g | 0.04 (-0.35 – 0.43) | | 0.843 | -1.21 – 1.28 | | 84.5 | | No/Yes | | | No/No | | | NS | | | 7 | |  |
| Goldsmith *et al*., 2016[62] | sIL-2R | 199/230 | 3 | Hedges’g | 0.63 (-0.11 – 1.38) | | 0.096 | -2.53 – 3.80 | | 91.9 | | No/No | | | No/No | | | NS | | | 10 | |  |
| Goldsmith *et al*., 2016[62] | sTNF-R1 | 133/310 | 4 | Hedges’g | 0.48 (0.09 – 0.86) | | 0.015 | -0.64 – 1.60 | | 55.9 | | No/No | | | No/No | | | NS | | | 10 | |  |
| Goldsmith *et al*., 2016[62] | TNF-Alpha | 236/296 | 9 | Hedges’g | 0.09 (-0.20 – 0.39) | | 0.532 | -0.75 – 0.94 | | 58.1 | | No/No | | | No/Yes | | | NS | | | 10 | |  |
| Bipolar Disorder (Mania) |  |  |  |  |  | |  |  | |  | |  | | |  | | |  | | |  | |  |
| Fernandes *et al.*, 2015 [101] | BDNF | 348/421 | 14 | Hedges’ g | -0.65 (-1.13 – -0.17) | | 0.007 | -2.57 – 1.26 | | 89.9 | | No/No | | | No/No | | | NS | | | 8 | |  |
| Fernandes *et al.*, 2016 [102] | CRP | 553/1149 | 14 | Hedges’ g | 0.87 (0.58 – 1.15) | | 0.000 | -0.17 – 1.90 | | 79.6 | | Yes/No | | | No/Yes | | | II | | | 7 | |  |
| Fernandes *et al.*, 2016 [103] | Leptin | 61/74 | 3 | Hedges’ g | -0.99 (-2.40 – 0.43) | | 0.171 | -6.99 – 5.01 | | 92.7 | | No/No | | | No/No | | | NS | | | 7 | |  |
| Looney *et al.*, 1997 [97] | NA,K-ATPASE ACTIVITY | 98/174 | 6 | SMD | 0.44 (-0.17 – 1.04) | | 0.159 | -1.45 – 2.33 | | 83.7 | | No/No | | | No/No | | | NS | | | 1 | |  |
| Goldsmith *et al*., 2016[62] | sIL-2R | 82/90 | 3 | Hedges’ g | 0.66 (0.35 – 0.97) | | 0.000 | -0.02 – 1.34 | | 0.0 | | Yes/No | | | No/Yes | | | III | | | 10 | |  |
| Goldsmith *et al*., 2016[62] | sIL-6R | 82/90 | 3 | Hedges’ g | 0.17 (-0.13 – 0.47) | | 0.256 | -0.49 – 0.83 | | 0.0 | | Yes/No | | | No/Yes | | | NS | | | 10 | |  |
| Goldsmith *et al*., 2016[62] | TNF-Alpha | 73/137 | 3 | Hedges’ g | 0.68 (-0.15 – 1.50) | | 0.107 | -2.69 – 4.04 | | 84.2 | | Yes/No | | | No/No | | | NS | | | 10 | |  |
| Tu *et al.*, 2016 [81] | IGF1 | 302/269 | 3 | Hedges’g | 0.51 (0.20 – 0.83) | | 0.001 | -0.62 – 1.65 | | 58.2 | | No/No | | | No/Yes | | | II | | | 6 | |  |
| Acute Exacerbation of Chronic Schizophrenia |  |  |  |  |  | |  |  | |  | |  | | |  | | |  | | |  | |  |
| Goldsmith *et al*., 2016[62] | IFN-Gamma | 162/266 | 4 | Hedges’ g | 0.37 (0.07 – 0.67) | | 0.017 | -0.44 – 1.18 | | 43.9 | | No/No | | | No/Yes | | | NS | | | 10 | |  |
| Goldsmith *et al*., 2016[62] | IL-1β | 131/151 | 3 | Hedges’ g | 0.20 (-0.37 – 0.76) | | 0.494 | -2.00 – 2.40 | | 73.0 | | No/No | | | No/No | | | NS | | | 10 | |  |
| Goldsmith *et al*., 2016[62] | IL-4 | 169/350 | 5 | Hedges’ g | -0.31 (-1.03 – 0.41) | | 0.403 | -2.72 – 2.11 | | 92.1 | | No/No | | | No/No | | | NS | | | 10 | |  |
| Goldsmith *et al*., 2016[62] | IL-6 | 278/468 | 9 | Hedges’ g | 0.76 (0.06 – 1.47) | | 0.034 | -1.78 – 3.31 | | 94.0 | | No/No | | | No/No | | | NS | | | 10 | |  |
| Goldsmith *et al*., 2016[62] | sIL-2R | 58/120 | 3 | Hedges’ g | 0.49 (-0.01 – 0.99) | | 0.053 | -1.29 – 2.27 | | 55.1 | | No/No | | | No/No | | | NS | | | 10 | |  |
| Goldsmith *et al*., 2016[62] | TGF-Beta | 243/382 | 6 | Hedges’ g | 0.49 (0.19 – 0.80) | | 0.002 | -0.39 – 1.37 | | 64.8 | | No/No | | | No/Yes | | | III | | | 10 | |  |
| Goldsmith *et al*., 2016[62] | TNF-Alpha | 269/449 | 7 | Hedges’ g | 0.29 (-0.58 – 1.15) | | 0.517 | -2.69 – 3.26 | | 95.7 | | No/Yes | | | No/No | | | NS | | | 10 | |  |
| Schizophrenia |  |  |  |  |  | |  |  | |  | |  | | |  | | |  | | |  | |  |
| Aleksovska *et al.*, 2014 [105] | 100SB | 893/785 | 20 | SMD | 1.11 (0.70 – 1.52) | | 0.000 | -0.81 – 3.03 | | 92.6 | | No/No | | | No/No | | | IV | | | 8 | |  |
| Lachance *et al.*, 2014 [106] | Anti-Gliadin IgA | 2289/1734 | 6 | SMD | 0.29 (0.14 – 0.45) | | 0.000 | -0.15 – 0.73 | | 72.8 | | No/No | | | No/Yes | | | II | | | 7 | |  |
| Lachance *et al.*, 2014 [106] | Anti-Gliadin IgG | 3137/2721 | 6 | SMD | 0.29 (0.06 – 0.53) | | 0.014 | -0.47 – 1.06 | | 93.7 | | No/No | | | No/Yes | | | NS | | | 7 | |  |
| Lachance *et al.*, 2014 [106] | Anti-TTG2 IgA | 2194/1680 | 4 | SMD | 0.10 (-0.02 – 0.23) | | 0.110 | -0.27 – 0.48 | | 64.0 | | No/No | | | Yes/Yes | | | NS | | | 7 | |  |
| Hoen *et al.*, 2013 [107] | arachidonic acid | 642/673 | 22 | Hedges’ g | 0.83 (0.48 – 1.17) | | 0.000 | -0.79 – 2.44 | | 87.4 | | Yes/No | | | No/Yes | | | II | | | 9 | |  |
| Fernandes et al., 2015 [108] | BDNF | 1854/1566 | 18 | Hedges’ g | -0.55 (-0.99 – -0.11) | | 0.015 | -2.56 – 1.46 | | 96.7 | | No/No | | | No/No | | | NS | | | 9 | |  |
| Flatow *et al.*, 2013 [109] | Catalase | 375/356 | 12 | Hedges’ g | 0.07 (-0.64 – 0.78) | | 0.844 | -2.71 – 2.85 | | 94.5 | | Yes/Yes | | | No/No | | | NS | | | 8 | |  |
| Aleksovska *et al.*, 2014 [105] | Cortisol | 1447/1020 | 42 | SMD | 0.25 (0.08 – 0.43) | | 0.004 | -0.75 – 1.25 | | 75.6 | | No/Yes | | | No/Yes | | | II | | | 8 | |  |
| Ciufolini *et al.*, 2014 [72] | Cortisol Delta to Social Stress Test | 59/65 | 3 | SMD | -0.24 (-0.61 – 0.12) | | 0.192 | -1.11 – 0.62 | | 4.7 | | No/No | | | No/No | | | NS | | | 4 | |  |
| Ciufolini *et al.*, 2014 [72] | Cortisol Level in Antecipation to Social Stress Test | 59/65 | 3 | SMD | -0.40 (-0.75 – -0.04) | | 0.030 | -1.18 – 0.39 | | 0.0 | | No/No | | | No/No | | | NS | | | 4 | |  |
| Ciufolini *et al.*, 2014 [72] | Cortisol Peak Response to Social Stress Test | 59/65 | 3 | SMD | -0.66 (-1.20 – -0.12) | | 0.016 | -2.53 – 1.21 | | 50.3 | | No/No | | | No/No | | | NS | | | 4 | |  |
| Zorn *et al.*, 2016[73] | Cortisol Reactivity to A Laboratory Psychosocial Stressor | 73/107 | 7 | SMD | -0.59 (-1.06 – -0.12) | | 0.014 | -1.83 – 0.65 | | 50.8 | | No/No | | | No/No | | | NS | | | 4 | |  |
| Fernandes *et al.*, 2016 [110] | CRP | 1793/80909 | 15 | Hedges’ g | 0.51 (0.27 – 0.75) | | 0.000 | -0.47 – 1.49 | | 91.9 | | No/No | | | No/Yes | | | III | | | 7 | |  |
| Hoen *et al.*, 2013 [107] | DHA | 606/639 | 20 | Hedges’ g | 0.81 (0.44 – 1.18) | | 0.000 | -0.89 – 2.51 | | 88.6 | | Yes/No | | | No/No | | | IV | | | 9 | |  |
| Hoen *et al.*, 2013 [107] | DPA | 467/469 | 16 | Hedges’ g | 1.14 (0.72 – 1.57) | | 0.000 | -0.61 – 2.90 | | 87.4 | | Yes/No | | | No/No | | | IV | | | 9 | |  |
| Greenhalgh *et al.*, 2016 [111] | Fasting Glucose | 911/870 | 19 | SMD | 0.21 (0.06 – 0.36) | | 0.008 | -0.34 – 0.75 | | 55.4 | | No/No | | | No/Yes | | | NS | | | 6 | |  |
| Greenhalgh *et al.*, 2016 [111] | Fasting Insulin | 532/418 | 10 | SMD | 0.39 (0.10 – 0.68) | | 0.009 | -0.58 – 1.35 | | 76.5 | | Yes/Yes | | | No/Yes | | | NS | | | 6 | |  |
| Wang *et al.*, 2016 [112] | Folate | 1773/1928 | 26 | WMD | -0.57 (-0.75 – -0.38) | | 0.000 | -1.46 – 0.33 | | 84.9 | | No/No | | | No/Yes | | | II | | | 8 | |  |
| Song *et al.*, 2014 [113] | Glutamate | 320/294 | 10 |  | 0.64 (0.21 – 1.06) | | 0.004 | -0.75 – 2.02 | | 79.9 | | No/No | | | No/No | | | IV | | | 5 | |  |
| Flatow *et al.*, 2013 [109] | GSH-PX | 352/191 | 7 | Hedges’ g | -0.65 (-1.25 – -0.05) | | 0.034 | -2.55 – 1.25 | | 87.5 | | No/No | | | No/No | | | NS | | | 8 | |  |
| Flatow *et al.*, 2013 [109] | GSH-PX | 375/356 | 12 | Hedges’ g | -0.25 (-0.93 – 0.43) | | 0.476 | -2.92 – 2.42 | | 94.0 | | No/Yes | | | No/No | | | NS | | | 8 | |  |
| Nishi *et al.*, 2014 [114] | Homocysteine | 1313/2022 | 11 | SMD | 0.76 (0.36 – 1.15) | | 0.000 | -0.68 – 2.20 | | 93.2 | | No/No | | | No/No | | | IV | | | 5 | |  |
| Guo *et al.*, 2015 [115] | IFN-Gamma | 378/358 | 10 | SMD | -0.57 (-0.97 – -0.16) | | 0.006 | -2.00 – 0.86 | | 84.9 | | No/No | | | No/No | | | NS | | | 4 | |  |
| Fang *et al.*, 2018 [116] | IL-17 | 313/238 | 5 | SMD | 0.17 (-0.32 – 0.65) | | 0.501 | -1.41 – 1.75 | | 86.9 | | No/Yes | | | No/No | | | NS | | | 5 | |  |
| Guo *et al.*, 2015 [115] | IL-2 | 262/244 | 8 | SMD | -0.90 (-1.35 – -0.44) | | 0.000 | -2.40 – 0.61 | | 82.7 | | No/No | | | No/No | | | IV | | | 4 | |  |
| Greenhalgh *et al.*, 2016 [111] | Insulin Resistance | 525/406 | 9 | SMD | 0.34 (0.11 – 0.57) | | 0.004 | -0.35 – 1.04 | | 63.5 | | No/Yes | | | No/Yes | | | III | | | 6 | |  |
| Plitman *et al.*, 2017 [117] | KYN-ACID | 217/210 | 5 | SMD | 0.50 (-0.32 – 1.33) | | 0.230 | -2.18 – 3.19 | | 93.2 | | No/No | | | No/No | | | NS | | | 8 | |  |
| Stubbs *et al.*, 2016 [118] | Leptin | 1585/1833 | 37 | Hedges’ g | 0.20 (0.02 – 0.37) | | 0.027 | -0.78 – 1.17 | | 82.2 | | No/Yes | | | No/Yes | | | NS | | | 9 | |  |
| Hoen *et al.*, 2013 [107] | Linoleic Acid | 519/512 | 18 | Hedges’ g | 0.72 (0.35 – 1.09) | | 0.000 | -0.84 – 2.28 | | 85.6 | | Yes/No | | | No/No | | | IV | | | 9 | |  |
| Flatow *et al.*, 2013 [109] | MDA | 266/158 | 7 | Hedges’ g | 0.83 (0.51 – 1.15) | | 0.000 | 0.05 – 1.61 | | 43.9 | | No/No | | | No/Yes | | | II | | | 8 | |  |
| Qin *et al.*, 2017 [119] | NGF | 894/799 | 13 | Hedges’ g | -0.63 (-0.95 – -0.32) | | 0.000 | -1.83 – 0.56 | | 88.4 | | No/No | | | No/Yes | | | II | | | 7 | |  |
| Pearlman, 2014 [87] | NMDAR | 224/713 | 4 | SMD | 0.35 (0.20 – 0.51) | | 0.000 | 0.10 – 0.61 | | 0.0 | | No/No | | | No/Yes | | | II | | | 7 | |  |
| Flatow *et al.*, 2013 [109] | NO | 223/79 | 3 | Hedges’ g | 0.87 (-0.10 – 1.84) | | 0.078 | -3.25 – 4.99 | | 92.0 | | No/No | | | No/No | | | NS | | | 8 | |  |
| Greenhalgh *et al.*, 2016 [111] | OGTT 2 HOURS | 237/189 | 4 | SMD | 0.64 (0.14 – 1.13) | | 0.011 | -1.02 – 2.30 | | 83.2 | | No/No | | | No/No | | | NS | | | 6 | |  |
| Rutigliano *et al.*, 2016 [92] | Oxytocin | 385/306 | 8 | Hedges’ g | -0.01 (-0.30 – 0.29) | | 0.972 | -0.93 – 0.92 | | 71.9 | | No/Yes | | | No/Yes | | | NS | | | 11 | |  |
| Flatow *et al.*, 2013 [109] | SOD | 559/272 | 10 | Hedges’ g | 0.36 (-0.54 – 1.25) | | 0.438 | -2.97 – 3.68 | | 96.8 | | No/Yes | | | No/No | | | NS | | | 8 | |  |
| Flatow *et al.*, 2013 [109] | SOD | 375/356 | 12 | Hedges’ g | -0.07 (-0.74 – 0.61) | | 0.843 | -2.72 – 2.58 | | 94.0 | | Yes/Yes | | | No/No | | | NS | | | 8 | |  |
| Flatow *et al.*, 2013 [109] | TBARS | 421/248 | 10 | Hedges’ g | 0.50 (0.00 – 1.00) | | 0.052 | -1.29 – 2.28 | | 88.0 | | No/Yes | | | No/No | | | NS | | | 8 | |  |
| Guo *et al.*, 2015 [115] | Th1/Th2 Interleukins Ratio | 136/157 | 5 | SMD | -0.62 (-1.25 – 0.01) | | 0.053 | -2.66 – 1.42 | | 85.2 | | No/No | | | No/No | | | NS | | | 4 | |  |
| Guo *et al.*, 2015 [115] | Th1/Th2 Interleukins Ratio | 278/332 | 6 | SMD | 0.37 (-0.26 – 1.00) | | 0.249 | -1.73 – 2.48 | | 92.6 | | No/No | | | No/No | | | NS | | | 4 | |  |
| Maia-de-Oliveira *et al.*, 2012 [120] | Total Nitrite | 505/339 | 10 | SMD | 0.28 (-0.20 – 0.77) | | 0.252 | -1.49 – 2.06 | | 90.8 | | No/Yes | | | No/No | | | NS | | | 6 | |  |
| Rutigliano *et al.*, 2016 [92] | Vasopressin | 307/323 | 11 | Hedges’ g | -0.56 (-0.97 – -0.14) | | 0.008 | -2.01 – 0.89 | | 82.3 | | No/No | | | No/No | | | NS | | | 11 | |  |
| Misiak *et al.*, 2018 [121] | VEGF | 446/449 | 7 | Hedges’ g | 0.45 (0.03 – 0.87) | | 0.037 | -0.93 – 1.83 | | 88.4 | | No/No | | | No/No | | | NS | | | 8 | |  |
| Tomioka *et al.*, 2018 [122] | Vitamin B6 | 840/1285 | 5 | SMD | -0.48 (-0.57 – -0.38) | | 0.000 | -0.61 – -0.34 | | 0.0 | | No/No | | | Yes/Yes | | | I | | | 5 | |  |
| Flatow *et al.*, 2013 [109] | Vitamin C | 49/53 | 3 | Hedges’ g | -0.84 (-1.25 – -0.44) | | 0.000 | -1.74 – 0.05 | | 0.0 | | No/No | | | No/No | | | IV | | | 8 | |  |
| Valipour *et al.*, 2014 [123] | Vitamin D | 890/6931 | 14 |  | -0.70 (-1.05 – -0.35) | | 0.000 | -2.07 – 0.66 | | 90.7 | | No/No | | | No/Yes | | | III | | | 6 | |  |
| Flatow *et al.*, 2013 [109] | Vitamin E | 46/38 | 3 | Hedges’ g | -0.89 (-1.38 – -0.41) | | 0.000 | -2.12 – 0.33 | | 10.7 | | No/No | | | No/No | | | IV | | | 8 | |  |
| Joe *et al.*, 2018 [124] | Zinc | 658/1008 | 10 |  | -0.67 (-1.16 – -0.19) | | 0.007 | -2.45 – 1.10 | | 93.9 | | No/No | | | No/No | | | NS | | | 7 | |  |
| Chronic Schizophrenia |  |  |  |  |  | |  |  | |  | |  | | |  | | |  | | |  | |  |
| Goldsmith *et al*., 2016[62] | IFN-Gamma | 232/168 | 5 | Hedges’ g | -1.13 (-2.76 – 0.51) | | 0.177 | -6.75 – 4.50 | | 97.9 | | Yes/No | | | No/No | | | NS | | | 10 | |  |
| Goldsmith *et al*., 2016[62] | IL-10 | 209/176 | 5 | Hedges’ g | -0.03 (-0.24 – 0.18) | | 0.801 | -0.34 – 0.28 | | 1.4 | | No/No | | | No/Yes | | | NS | | | 10 | |  |
| Goldsmith *et al*., 2016[62] | IL-1β | 421/353 | 5 | Hedges’ g | 1.05 (0.23 – 1.88) | | 0.012 | -1.75 – 3.86 | | 96.1 | | No/No | | | No/No | | | NS | | | 10 | |  |
| Goldsmith *et al*., 2016[62] | IL-2 | 222/197 | 6 | Hedges’ g | -0.15 (-0.83 – 0.52) | | 0.653 | -2.40 – 2.09 | | 91.0 | | No/Yes | | | No/No | | | NS | | | 10 | |  |
| Goldsmith *et al*., 2016[62] | IL-6 | 802/724 | 14 | Hedges’ g | 0.11 (-0.21 – 0.43) | | 0.488 | -1.15 – 1.37 | | 88.2 | | No/Yes | | | No/Yes | | | NS | | | 10 | |  |
| Goldsmith *et al*., 2016[62] | sIL-2R | 116/135 | 3 | Hedges’ g | 0.67 (0.41 – 0.92) | | 0.000 | 0.10 – 1.23 | | 0.0 | | No/No | | | No/Yes | | | II | | | 10 | |  |
| Goldsmith *et al*., 2016[62] | TNF-Alpha | 599/559 | 11 | Hedges’ g | 0.36 (-0.10 – 0.82) | | 0.130 | -1.38 – 2.09 | | 92.5 | | No/No | | | No/No | | | NS | | | 10 | |  |
| First Episode Psychosis |  |  |  |  |  | |  |  | |  | |  | | |  | | |  | | |  | |  |
| Firth et al., 2018 [125] | Calcium | 162/76 | 3 | Hedges’ g | -0.30 (-1.03 – 0.43) | | 0.421 | -3.30 – 2.70 | | 84.8 | | No/No | | | No/No | | | NS | | | 7 | |  |
| Firth et al., 2018 [125] | Copper | 83/59 | 3 | Hedges’ g | 1.06 (-0.74 – 2.87) | | 0.248 | -6.72 – 8.85 | | 95.3 | | No/No | | | No/No | | | NS | | | 7 | |  |
| Chaumette *et al.*, 2016 [9] | Cortisol | 215/226 | 6 | SMD | -0.02 (-0.41 – 0.38) | | 0.927 | -1.22 – 1.18 | | 74.6 | | No/No | | | No/No | | | NS | | | 4 | |  |
| Berger *et al.*, 2016 [126] | Cortisol Awakening Response (Car) | 251/216 | 6 | Hedges’g | -0.54 (-0.73 – -0.36) | | 0.000 | -0.79 – -0.30 | | 0.0 | | No/No | | | No/Yes | | | II | | | 7 | |  |
| Pillinger *et al.*, 2017 [127] | Fasting Glucose | 718/599 | 14 | Hedges’g | 0.20 (0.02 – 0.38) | | 0.027 | -0.38 – 0.78 | | 58.3 | | Yes/No | | | No/Yes | | | NS | | | 7 | |  |
| Pillinger *et al.*, 2017 [127] | Fasting Insulin | 512/448 | 11 | Hedges’g | 0.41 (0.09 – 0.72) | | 0.011 | -0.69 – 1.51 | | 80.8 | | No/No | | | No/Yes | | | NS | | | 7 | |  |
| Firth et al., 2018 [125] | Folate | 346/324 | 6 | Hedges’g | -0.62 (-1.18 – -0.07) | | 0.027 | -2.45 – 1.21 | | 92.4 | | No/No | | | No/No | | | NS | | | 7 | |  |
| Pillinger *et al.*, 2017 [127] | Glucose After an Oral Glucose Tolerance Test | 271/237 | 4 | Hedges’g | 0.60 (0.16 – 1.04) | | 0.007 | -0.87 – 2.08 | | 82.4 | | No/No | | | No/No | | | NS | | | 7 | |  |
| Pillinger *et al.*, 2017 [127] | Insulin Resistance | 485/400 | 10 | Hedges’g | 0.35 (0.14 – 0.55) | | 0.001 | -0.26 – 0.95 | | 55.3 | | No/Yes | | | No/Yes | | | III | | | 7 | |  |
| Firth et al., 2018 [125] | Magnesium | 202/85 | 4 | Hedges’g | -0.71 (-2.27 – 0.85) | | 0.374 | -6.30 – 4.88 | | 96.6 | | No/No | | | No/No | | | NS | | | 7 | |  |
| Firth et al., 2018 [125] | Sodium | 110/83 | 3 | Hedges’g | 2.09 (-1.98 – 6.15) | | 0.314 | -15.67 – 19.85 | | 98.9 | | No/No | | | No/No | | | NS | | | 7 | |  |
| Firth et al., 2018 [125] | Vitamin B12 | 319/327 | 4 | Hedges’g | -0.06 (-0.22 – 0.10) | | 0.469 | -0.32 – 0.20 | | 0.0 | | No/No | | | No/Yes | | | NS | | | 7 | |  |
| Firth et al., 2018 [125] | Vitamin D | 429/477 | 7 | Hedges’g | -1.06 (-1.99 – -0.12) | | 0.027 | -4.30 – 2.19 | | 97.2 | | No/No | | | No/No | | | NS | | | 7 | |  |
| Firth et al., 2018 [125] | Vitamin E | 134/143 | 4 | Hedges’g | -1.09 (-2.54 – 0.36) | | 0.140 | -6.28 – 4.10 | | 96.2 | | No/No | | | No/No | | | NS | | | 7 | |  |
| Firth et al., 2018 [125] | Zinc | 83/59 | 3 | Hedges’g | -0.82 (-2.86 – 1.22) | | 0.430 | -9.66 – 8.02 | | 96.3 | | No/No | | | No/No | | | NS | | | 7 | |  |
| Alzheimer |  |  |  |  |  | |  |  | |  | |  | | |  | | |  | | |  | |  |
| Annweiler *et al.*, 2013[128] | 25 OH Vitamin D | 357/648 | 7 | SMD | 1.40 (0.26 – 2.54) | | 0.016 | -2.58 – 5.38 | | 98.0 | | No/No | | | No/No | | | NS | | | 11 | |  |
| Shanthi *et al.*, 2015[129] | A Beta 42 | 1542/2323 | 25 | WMD | 0.41 (0.03 – 0.78) | | 0.032 | -1.51 – 2.32 | | 95.4 | | Yes/No | | | No/No | | | NS | | | 8 | |  |
| Lai *et al.,* 2017 [130] | Adiponectin | 426/515 | 7 | SMD | 0.20 (-0.50 – 0.89) | | 0.580 | -2.21 – 2.60 | | 95.7 | | No/No | | | No/No | | | NS | | | 9 | |  |
| Mullan *et al.,* 2018 [131] | Alpha-Carotene | 327/269 | 8 | SMD | -0.52 (-0.86 – -0.18) | | 0.003 | -1.57 – 0.53 | | 73.0 | | No/No | | | No/Yes | | | III | | | 7 | |  |
| Xu *et al.,* 2018[132] | Aluminium | 609/670 | 17 | SMD | 1.08 (0.66 – 1.50) | | 0.000 | -0.69 – 2.85 | | 89.5 | | No/No | | | No/No | | | IV | | | 7 | |  |
| Shi *et al.*, 2017[133] | Amyloid Β Protein Precursor | 470/482 | 16 | SMD | -1.87 (-2.33 – -1.41) | | 0.000 | -3.77 – 0.02 | | 88.0 | | Yes/No | | | No/No | | | IV | | | 8 | |  |
| Lai *et al.,* 2017 [130] | ANG-2I | 220/227 | 3 | SMD | -0.12 (-0.75 – 0.52) | | 0.721 | -2.73 – 2.50 | | 86.6 | | No/No | | | No/No | | | NS | | | 9 | |  |
| Wang *et al.*, 2014[134] | Apolipoprotein E | 1498/2250 | 8 | SMD | -0.34 (-0.46 – -0.21) | | 0.000 | -0.69 – 0.02 | | 62.1 | | No/No | | | Yes/Yes | | | II | | | 9 | |  |
| Song *et al.*, 2011[135] | AΒ1-40 | 1240/3615 | 17 | WMD | 0.19 (0.05 – 0.32) | | 0.006 | -0.28 – 0.66 | | 63.5 | | No/No | | | Yes/Yes | | | NS | | | 6 | |  |
| Song *et al.*, 2011[135] | AΒ1-42 | 1432/3971 | 19 | WMD | 0.06 (-0.09 – 0.21) | | 0.404 | -0.53 – 0.65 | | 74.6 | | No/No | | | No/Yes | | | NS | | | 6 | |  |
| Song *et al.*, 2011[135] | AΒ1-42/AΒ1-40 Ratios | 533/3074 | 6 | WMD | 0.15 (-0.04 – 0.34) | | 0.118 | -0.41 – 0.71 | | 72.8 | | No/Yes | | | No/Yes | | | NS | | | 6 | |  |
| De Wilde *et al.*, 2017 [136] | B6 Vitamin | 192/199 | 6 | Hedges’g | -0.71 (-1.54 – 0.11) | | 0.091 | -3.39 – 1.97 | | 91.5 | | No/No | | | No/No | | | NS | | | 4 | |  |
| Du *et al.*, 2018[137] | BDNF | 2157/1668 | 31 | Hedges’g | -0.31 (-0.51 – -0.11) | | 0.003 | -1.40 – 0.78 | | 88.3 | | No/Yes | | | No/Yes | | | II | | | 7 | |  |
| Mullan *et al.,* 2018 [131] | Beta-Carotene | 701/633 | 13 | SMD | -0.57 (-1.02 – -0.13) | | 0.011 | -2.32 – 1.17 | | 92.4 | | Yes/No | | | No/No | | | NS | | | 7 | |  |
| Mullan *et al.,* 2018 [131] | Beta-Cryptoxanthin | 174/145 | 4 | SMD | -0.73 (-1.53 – 0.07) | | 0.073 | -3.51 – 2.04 | | 90.3 | | No/No | | | No/No | | | NS | | | 7 | |  |
| Xu *et al.,* 2018[132] | Cadmium | 435/439 | 8 | SMD | 0.62 (0.12 – 1.11) | | 0.014 | -1.07 – 2.31 | | 90.5 | | No/No | | | No/No | | | NS | | | 6 | |  |
| Lai *et al.,* 2017 [130] | CCL-2 | 748/482 | 12 | SMD | 0.50 (-0.12 – 1.12) | | 0.111 | -1.92 – 2.92 | | 95.5 | | No/No | | | No/No | | | NS | | | 9 | |  |
| Lai *et al.,* 2017 [130] | CCL-3 | 170/167 | 4 | SMD | 0.10 (-0.30 – 0.50) | | 0.618 | -1.08 – 1.28 | | 59.9 | | No/No | | | No/No | | | NS | | | 9 | |  |
| Lai *et al.,* 2017 [130] | CCL-5 | 365/298 | 6 | SMD | 0.34 (-1.01 – 1.70) | | 0.620 | -4.09 – 4.78 | | 97.3 | | No/Yes | | | No/No | | | NS | | | 9 | |  |
| De Wilde *et al.*, 2017 [136] | Choline | 87/76 | 4 | Hedges’ g | -0.58 (-1.11 – -0.04) | | 0.036 | -2.15 – 1.00 | | 59.4 | | No/No | | | No/No | | | NS | | | 4 | |  |
| Yang *et al.*, 2017[138] | Clusterin | 1677/3149 | 12 | SMD | 1.84 (0.85 – 2.84) | | 0.000 | -2.15 – 5.84 | | 99.4 | | No/No | | | No/No | | | IV | | | 8 | |  |
| Li *et al.*, [139] | Copper | 2128/2889 | 35 | SMD | 0.68 (0.40 – 0.96) | | 0.000 | -0.99 – 2.35 | | 94.5 | | No/No | | | No/Yes | | | II | | | 9 | |  |
| Lai *et al.,* 2017 [130] | CRP | 1776/2439 | 20 | SMD | 0.27 (-0.09 – 0.63) | | 0.147 | -1.43 – 1.96 | | 95.7 | | No/Yes | | | No/No | | | NS | | | 9 | |  |
| Lai *et al.,* 2017 [130] | CXCL-10 | 138/144 | 3 | SMD | 0.97 (-0.06 – 1.99) | | 0.065 | -3.25 – 5.18 | | 85.6 | | No/No | | | No/No | | | NS | | | 9 | |  |
| Schneider *et al.*, 1992 [140] | DHEA | 2787/3356 | 4 | SMD | 0.02 (-0.03 – 0.07) | | 0.469 | -0.06 – 0.10 | | 0.0 | | No/No | | | Yes/Yes | | | NS | | | 1 | |  |
| De Wilde *et al.*, 2017 [136] | DHA | 483/1245 | 13 | Hedges’ g | -1.19 (-1.78 – -0.59) | | 0.000 | -3.56 – 1.19 | | 94.7 | | No/No | | | No/No | | | IV | | | 4 | |  |
| Lai *et al.,* 2017 [130] | EGF | 521/508 | 5 | SMD | 2.22 (0.40 – 4.04) | | 0.017 | -4.07 – 8.50 | | 98.8 | | No/No | | | No/No | | | NS | | | 9 | |  |
| De Wilde *et al.*, 2017 [136] | EPA | 457/1117 | 12 | Hedges’ g | -0.34 (-0.58 – -0.09) | | 0.008 | -1.14 – 0.47 | | 68.5 | | No/No | | | No/Yes | | | NS | | | 4 | |  |
| Lai *et al.,* 2017 [130] | E-Selectin | 305/205 | 5 | SMD | 0.26 (-0.14 – 0.66) | | 0.201 | -0.97 – 1.49 | | 76.8 | | No/Yes | | | No/No | | | NS | | | 9 | |  |
| Xu *et al.*, 2016 [141] | Estradiol | 647/1006 | 17 | SMD | -0.02 (-0.27 – 0.24) | | 0.903 | -0.98 – 0.95 | | 79.0 | | No/No | | | No/Yes | | | NS | | | 7 | |  |
| Lai *et al.,* 2017 [130] | Fibrinogen | 232/267 | 5 | SMD | 0.17 (-0.11 – 0.45) | | 0.228 | -0.58 – 0.92 | | 52.0 | | Yes/No | | | No/Yes | | | NS | | | 9 | |  |
| Lopes da Silva *et al.*, 2014 [142] | Folate | 2108/2447 | 31 | SMD | -0.74 (-0.96 – -0.52) | | 0.000 | -1.95 – 0.47 | | 90.9 | | No/No | | | No/Yes | | | III | | | 4 | |  |
| Shen *et al.*, 2015 [143] | Folic Acid | 2496/2776 | 34 | SMD | -0.83 (-1.08 – -0.57) | | 0.000 | -2.29 – 0.64 | | 93.9 | | Yes/No | | | No/Yes | | | II | | | 9 | |  |
| Lai *et al.,* 2017 [130] | G-CSF | 483/408 | 6 | SMD | 0.20 (-0.29 – 0.68) | | 0.430 | -1.41 – 1.80 | | 90.9 | | No/Yes | | | No/No | | | NS | | | 9 | |  |
| Ho *et al.*, 2011 [144] | Hct Levels | 514/305 | 8 | SMD | 0.48 (0.23 – 0.73) | | 0.000 | -0.23 – 1.20 | | 58.8 | | No/No | | | No/Yes | | | III | | | 5 | |  |
| Shen *et al.*, 2015 [143] | Homocysteine | 2510/2254 | 37 | SMD | 1.34 (1.06 – 1.61) | | 0.000 | -0.30 – 2.97 | | 94.3 | | Yes/No | | | No/Yes | | | II | | | 9 | |  |
| Lai *et al.,* 2017 [130] | HSCPR | 896/474 | 7 | SMD | 0.55 (0.25 – 0.85) | | 0.000 | -0.35 – 1.45 | | 76.7 | | No/No | | | No/Yes | | | III | | | 9 | |  |
| Lai *et al.,* 2017 [130] | ICAM-1 | 995/666 | 12 | SMD | 0.15 (-0.19 – 0.50) | | 0.390 | -1.15 – 1.46 | | 89.3 | | Yes/Yes | | | No/Yes | | | NS | | | 9 | |  |
| Lai *et al.,* 2017 [130] | IFN-GAMMA | 382/284 | 9 | SMD | 0.46 (0.01 – 0.91) | | 0.045 | -1.02 – 1.94 | | 82.9 | | Yes/No | | | No/No | | | NS | | | 9 | |  |
| Hu *et al.*, 2016[145] | IGFBP-3 | 510/499 | 7 | SMD | -0.33 (-0.70 – 0.04) | | 0.076 | -1.51 – 0.85 | | 86.1 | | No/No | | | No/No | | | NS | | | 7 | |  |
| Lai *et al.,* 2017 [130] | IL-1 Receptor Antagonist | 343/398 | 4 | SMD | -3.66 (-6.69 – -0.64) | | 0.018 | -14.59 – 7.26 | | 99.5 | | No/No | | | No/No | | | NS | | | 9 | |  |
| Lai *et al.,* 2017 [130] | IL-10 | 848/915 | 14 | SMD | -0.01 (-0.24 – 0.23) | | 0.966 | -0.91 – 0.90 | | 82.1 | | No/Yes | | | No/Yes | | | NS | | | 9 | |  |
| Lai *et al.,* 2017 [130] | IL-11 | 271/216 | 3 | SMD | -0.05 (-0.67 – 0.56) | | 0.862 | -2.59 – 2.48 | | 85.2 | | No/Yes | | | No/No | | | NS | | | 9 | |  |
| Lai *et al.,* 2017 [130] | IL-12 | 238/199 | 7 | SMD | 0.52 (-0.18 – 1.23) | | 0.146 | -1.80 – 2.85 | | 88.7 | | No/No | | | No/No | | | NS | | | 9 | |  |
| Lai *et al.,* 2017 [130] | IL-18 | 369/302 | 8 | SMD | 0.95 (0.14 – 1.75) | | 0.021 | -1.87 – 3.76 | | 95.5 | | No/No | | | No/No | | | NS | | | 9 | |  |
| Lai *et al.,* 2017 [130] | IL-1Alpha | 468/369 | 8 | SMD | 0.21 (-0.38 – 0.80) | | 0.480 | -1.82 – 2.24 | | 92.6 | | No/No | | | No/No | | | NS | | | 9 | |  |
| Lai *et al.,* 2017 [130] | IL-2 | 336/248 | 6 | SMD | 0.88 (0.12 – 1.63) | | 0.022 | -1.60 – 3.36 | | 93.3 | | Yes/No | | | No/No | | | NS | | | 9 | |  |
| Lai *et al.,* 2017 [130] | IL-3 | 248/212 | 3 | SMD | 0.35 (-0.49 – 1.19) | | 0.415 | -3.14 – 3.84 | | 89.5 | | No/No | | | No/No | | | NS | | | 9 | |  |
| Lai *et al.,* 2017 [130] | IL-4 | 201/220 | 5 | SMD | -0.10 (-0.29 – 0.10) | | 0.321 | -0.37 – 0.18 | | 0.0 | | No/No | | | No/Yes | | | NS | | | 9 | |  |
| Lai *et al.,* 2017 [130] | IL-8 | 768/720 | 14 | SMD | 0.27 (-0.46 – 1.01) | | 0.466 | -2.70 – 3.25 | | 97.0 | | No/Yes | | | No/No | | | NS | | | 9 | |  |
| Ma *et al.*, 2016[146] | Insulin | 617/653 | 13 | WMD | 1.13 (0.51 – 1.74) | | 0.000 | -1.34 – 3.59 | | 95.5 | | No/No | | | No/No | | | IV | | | 8 | |  |
| Li *et al.*, [139] | Iron | 1385/1635 | 25 | SMD | -0.12 (-0.52 – 0.28) | | 0.544 | -2.20 – 1.95 | | 95.7 | | No/No | | | No/No | | | NS | | | 9 | |  |
| Xu *et al.,* 2018[132] | Lead | 1000/1005 | 10 | SMD | -0.23 (-0.39 – -0.07) | | 0.004 | -0.65 – 0.19 | | 48.0 | | No/No | | | No/Yes | | | III | | | 7 | |  |
| Zhou *et al.*, 2018[147] | Leptin | 1224/970 | 16 | SMD | -0.63 (-1.03 – -0.23) | | 0.002 | -2.36 – 1.10 | | 94.6 | | No/No | | | No/No | | | IV | | |  | |  |
| Mullan *et al.,* 2018 [131] | Lutein | 210/178 | 5 | SMD | -0.69 (-1.17 – -0.22) | | 0.004 | -2.18 – 0.80 | | 79.1 | | No/No | | | No/No | | | IV | | | 7 | |  |
| Mullan *et al.,* 2018 [131] | Lycopene | 424/319 | 7 | SMD | -0.77 (-1.37 – -0.17) | | 0.012 | -2.80 – 1.27 | | 92.6 | | No/No | | | No/No | | | NS | | | 7 | |  |
| Schrag *et al.*, 2013 [148] | Malondialdehyde | 288/290 | 8 | Hedges’ g | 1.58 (0.31 – 2.84) | | 0.014 | -2.88 – 6.03 | | 97.3 | | No/No | | | No/No | | | NS | | | 6 | |  |
| Du et al., 2017[149] | Manganese | 836/2082 | 14 | SMD | -0.35 (-0.58 – -0.12) | | 0.003 | -1.20 – 0.49 | | 81.6 | | No/No | | | No/Yes | | | III | | | 8 | |  |
| Lai *et al.,* 2017 [130] | MCP-3 | 281/235 | 4 | SMD | 0.61 (-0.21 – 1.42) | | 0.144 | -2.22 – 3.43 | | 91.6 | | No/No | | | No/No | | | NS | | | 9 | |  |
| Lai *et al.,* 2017 [130] | M-CSF | 358/297 | 6 | SMD | -0.03 (-0.25 – 0.18) | | 0.765 | -0.49 – 0.43 | | 28.6 | | No/No | | | No/Yes | | | NS | | | 9 | |  |
| Xu *et al.,* 2018[132] | Mercury | 514/519 | 7 | SMD | 0.55 (0.14 – 0.95) | | 0.008 | -0.73 – 1.83 | | 85.1 | | No/No | | | No/No | | | NS | | | 7 | |  |
| Beydoun et al., 2014 [150] | N-3 Fatty Acid | 7715/7719 | 5 |  | -0.05 (-0.10 – 0.00) | | 0.038 | -0.16 – 0.06 | | 36.5 | | No/No | | | Yes/Yes | | | NS | | | 5 | |  |
| Squitti *et al.*, 2014[151] | Non-Cp Copper (mol/L) | 599/867 | 10 | SMD | 0.63 (0.42 – 0.84) | | 0.000 | -0.03 – 1.29 | | 64.8 | | No/No | | | No/Yes | | | III | | | 7 | |  |
| Schrag *et al.*, 2013[148] | Oxidized LDL | 122/80 | 5 | Hedges’ g | 1.46 (0.15 – 2.78) | | 0.029 | -2.88 – 5.80 | | 92.8 | | No/No | | | No/No | | | NS | | | 6 | |  |
| Ho *et al.*, 2011 [144] | High-Hct Levels | 831/2708 | 12 | SMD | 0.59 (0.37 – 0.80) | | 0.000 | -0.16 – 1.34 | | 79.3 | | Yes/No | | | No/Yes | | | III | | | 5 | |  |
| De Wilde *et al.*, 2017 [136] | Selenium | 660/536 | 17 | Hedges’ g | -0.49 (-0.85 – -0.14) | | 0.006 | -1.96 – 0.97 | | 86.7 | | No/No | | | No/No | | | NS | | | 4 | |  |
| Lai *et al.,* 2017 [130] | TGF-BETA | 405/307 | 9 | SMD | -0.07 (-1.04 – 0.91) | | 0.896 | -3.58 – 3.45 | | 96.7 | | No/Yes | | | No/No | | | NS | | | 9 | |  |
| Shanthi *et al.*, 2015[129] | Total Tau | 279/322 | 6 | WMD | 0.43 (-1.13 – 1.99) | | 0.586 | -4.91 – 5.78 | | 98.1 | | No/No | | | No/No | | | NS | | | 8 | |  |
| Xu *et al.*, 2016 [141] | Total Teststerone | 645/951 | 13 | SMD | -0.01 (-0.27 – 0.25) | | 0.929 | -0.92 – 0.90 | | 79.8 | | No/Yes | | | No/Yes | | | NS | | | 6 | |  |
| Liu *et al.*, 2018 [152] | TREM2 | 245/311 | 3 | SMD | 0.10 (-0.07 – 0.28) | | 0.226 | -0.27 – 0.48 | | 0.0 | | No/No | | | No/Yes | | | NS | | | 9 | |  |
| Du *et al.*, 2016[153] | Uric Acid | 1128/2498 | 21 | SMD | -0.74 (-1.26 – -0.23) | | 0.005 | -3.26 – 1.78 | | 97.0 | | No/No | | | No/No | | | IV | | | 4 | |  |
| Lai *et al.,* 2017 [130] | VCAM-1 | 626/340 | 6 | SMD | 0.31 (0.02 – 0.60) | | 0.036 | -0.53 – 1.15 | | 69.7 | | No/No | | | No/Yes | | | NS | | | 9 | |  |
| Lai *et al.,* 2017 [130] | VEGF | 659/577 | 9 | SMD | -0.95 (-1.92 – 0.02) | | 0.055 | -4.45 – 2.55 | | 97.6 | | No/No | | | No/No | | | NS | | | 9 | |  |
| Mullan *et al.,* 2018 [131] | Vitamin A | 752/1104 | 15 | SMD | -0.87 (-1.37 – -0.37) | | 0.001 | -2.99 – 1.25 | | 95.0 | | No/No | | | No/No | | | IV | | | 7 | |  |
| Lopes da Silva *et al.*, 2014 [142] | Vitamin B12 | 2264/2784 | 37 | SMD | -0.38 (-0.57 – -0.20) | | 0.000 | -1.45 – 0.69 | | 88.0 | | No/No | | | No/Yes | | | III | | | 4 | |  |
| Mullan *et al.,* 2018 [131] | Vitamin C | 623/491 | 16 | SMD | -0.99 (-1.45 – -0.53) | | 0.000 | -2.91 – 0.93 | | 91.0 | | No/No | | | No/No | | | IV | | | 7 | |  |
| Mullan *et al.,* 2018 [131] | Vitamin E | 1554/1872 | 31 | SMD | -1.05 (-1.37 – -0.73) | | 0.000 | -2.84 – 0.74 | | 93.8 | | No/No | | | No/Yes | | | II | | | 7 | |  |
| Mullan *et al.,* 2018 [131] | Zeaxanthin | 210/178 | 5 | SMD | -0.66 (-1.38 – 0.06) | | 0.073 | -3.04 – 1.73 | | 90.6 | | No/No | | | No/No | | | NS | | | 7 | |  |
| Ventriglia *et al.*, 2015[154] | Zinc | 979/1912 | 22 | SMD | -0.32 (-0.70 – 0.07) | | 0.108 | -2.19 – 1.56 | | 94.2 | | No/No | | | No/No | | | NS | | | 9 | |  |
| Autism |  |  |  |  |  | |  |  | |  | |  | | |  | | |  | | |  | |  |
| Frustaci *et al.*, 2012 [155] | Vitamin B12 (Fasting) | 151/136 | 4 | SMD | -0.03 (-0.51 – 0.45) | | 0.894 | -1.58 – 1.51 | | 73.9 | | No/Yes | | | No/No | | | NS | | | 4 | |  |
| Frustaci *et al.*, 2012 [155] | Vitamin B9 (Fasting) | 167/147 | 5 | SMD | 0.09 (-0.14 – 0.31) | | 0.438 | -0.23 – 0.41 | | 0.0 | | No/Yes | | | No/Yes | | | NS | | | 4 | |  |
| Frustaci *et al.*, 2012 [155] | Vitamin C (Fasting) | 112/151 | 3 | SMD | 0.42 (-0.44 – 1.28) | | 0.343 | -3.22 – 4.06 | | 90.5 | | No/Yes | | | No/No | | | NS | | | 4 | |  |
| Frustaci *et al.*, 2012 [155] | Vitamin E (Fasting) | 112/151 | 3 | SMD | -1.08 (-2.01 – -0.15) | | 0.023 | -5.01 – 2.85 | | 90.9 | | No/No | | | No/No | | | NS | | | 4 | |  |
| Wang et al., 2016 [156] | 25 OH Vitamin D | 870/782 | 11 | SMD | -1.21 (-1.80 – -0.62) | | 0.000 | -3.47 – 1.05 | | 96.4 | | No/No | | | No/No | | | IV | | | 9 | |  |
| Gabriele *et al.*, 2014[157] | 5HT Levels | 481/534 | 23 | SMD | 0.74 (0.56 – 0.92) | | 0.000 | 0.16 – 1.32 | | 37.5 | | No/No | | | No/Yes | | | II | | | 6 | |  |
| Saghazadeh *et al.*, 2017 [158] | Antimony | 181/185 | 4 | SMD | 0.24 (0.03 – 0.45) | | 0.023 | -0.10 – 0.58 | | 0.0 | | No/No | | | No/Yes | | | NS | | | 9 | |  |
| Mazahery *et al.*, 2017 [159] | Arachidonic Acid | 558/554 | 13 | SMD | -0.83 (-1.48 – -0.17) | | 0.013 | -3.44 – 1.79 | | 95.5 | | No/No | | | No/No | | | NS | | | 8 | |  |
| Saghazadeh *et al.*, 2017 [160] | BDNF | 887/901 | 23 | SMD | 0.47 (0.07 – 0.86) | | 0.021 | -1.46 – 2.40 | | 92.1 | | No/Yes | | | No/No | | | NS | | | 6 | |  |
| Saghazadeh *et al.*, 2017 [158] | Cadmium | 271/287 | 9 | SMD | -0.30 (-0.48 – -0.12) | | 0.001 | -0.59 – 0.00 | | 10.2 | | No/No | | | No/Yes | | | III | | | 9 | |  |
| Main *et al.*, 2012 [161] | Cystathione | 170/160 | 6 | SMD | -0.05 (-0.51 – 0.42) | | 0.847 | -1.38 – 1.29 | | 70.0 | | No/No | | | No/No | | | NS | | | 10 | |  |
| Frustaci *et al.*, 2012 [155] | Cystathionine | 170/139 | 4 | SMD | 0.04 (-0.52 – 0.60) | | 0.888 | -1.80 – 1.88 | | 79.5 | | No/Yes | | | No/No | | | NS | | | 4 | |  |
| Main *et al.*, 2012 [161] | Cysteine | 275/285 | 9 | SMD | -0.83 (-1.46 – -0.20) | | 0.010 | -3.04 – 1.38 | | 90.5 | | No/No | | | No/No | | | NS | | | 10 | |  |
| Mazahery *et al.*, 2017 [159] | Docosahexaenoic Acid (Dha) | 598/594 | 14 | SMD | -1.61 (-2.48 – -0.73) | | 0.000 | -5.27 – 2.06 | | 97.3 | | No/No | | | No/No | | | IV | | | 8 | |  |
| Mazahery *et al.*, 2017 [159] | Eicosapentaenoic Acid (Epa) | 448/444 | 11 | SMD | -0.44 (-0.90 – 0.03) | | 0.065 | -2.16 – 1.28 | | 90.2 | | No/No | | | No/No | | | NS | | | 8 | |  |
| Tseng *et al.*, 2018 [162] | Ferritin | 391/352 | 4 | Hedges’ g | 0.02 (-0.48 – 0.51) | | 0.949 | -1.65 – 1.68 | | 84.8 | | No/Yes | | | No/No | | | NS | | | 11 | |  |
| Zheng *et al.*, 2016 [163] | Glutamate | 446/434 | 12 | SMD | 0.99 (0.58 – 1.40) | | 0.000 | -0.50 – 2.48 | | 85.9 | | No/No | | | No/No | | | IV | | | 7 | |  |
| Frustaci *et al.*, 2012 [155] | Glutathione Peroxidase | 133/109 | 5 | SMD | -1.65 (-3.41 – 0.11) | | 0.066 | -7.63 – 4.32 | | 96.6 | | No/No | | | No/No | | | NS | | | 4 | |  |
| Frustaci *et al.*, 2012 [155] | Glutathione Peroxidase (Fasting) | 102/101 | 3 | SMD | 0.46 (-2.04 – 2.95) | | 0.721 | -10.44 – 11.35 | | 98.3 | | No/Yes | | | No/No | | | NS | | | 4 | |  |
| Frustaci *et al.*, 2012 [155] | GSH (Fasting) | 213/291 | 4 | SMD | -1.45 (-1.67 – -1.22) | | 0.000 | -1.93 – -0.96 | | 18.4 | | No/No | | | No/Yes | | | II | | | 4 | |  |
| Main *et al.*, 2012 [161] | GSSG | 243/332 | 6 | SMD | 1.25 (0.94 – 1.56) | | 0.000 | 0.39 – 2.11 | | 60.1 | | No/No | | | No/Yes | | | II | | | 10 | |  |
| Frustaci *et al.*, 2012 [155] | GSSG (Fasting) | 233/324 | 5 | SMD | 1.08 (0.88 – 1.29) | | 0.000 | 0.69 – 1.47 | | 15.6 | | No/No | | | No/Yes | | | II | | | 4 | |  |
| Main *et al.*, 2012 [161] | Homocysteine | 239/227 | 9 | SMD | -0.18 (-0.41 – 0.05) | | 0.125 | -0.65 – 0.30 | | 24.5 | | No/No | | | No/Yes | | | NS | | | 10 | |  |
| Masi *et al.*, 2015 [164] | IFN-Gamma | 121/118 | 5 | Hedges’ g | 1.23 (0.20 – 2.27) | | 0.020 | -2.22 – 4.69 | | 92.6 | | Yes/No | | | No/No | | | NS | | | 7 | |  |
| Masi *et al.*, 2015 [164] | IL-1Beta | 199/187 | 5 | Hedges’ g | 0.68 (0.27 – 1.08) | | 0.001 | -0.53 – 1.88 | | 70.0 | | No/No | | | No/No | | | IV | | | 7 | |  |
| Masi *et al.*, 2015 [164] | IL-23 | 148/128 | 4 | Hedges’ g | 0.07 (-0.84 – 0.99) | | 0.875 | -3.14 – 3.28 | | 92.6 | | No/Yes | | | No/No | | | NS | | | 7 | |  |
| Tseng *et al.*, 2018 [162] | Iron | 401/445 | 10 | Hedges’ g | -0.11 (-0.48 – 0.25) | | 0.539 | -1.41 – 1.18 | | 86.1 | | No/Yes | | | No/No | | | NS | | | 11 | |  |
| Saghazadeh *et al.*, 2017 [158] | Lead | 391/327 | 8 | SMD | 0.44 (0.02 – 0.85) | | 0.042 | -0.97 – 1.84 | | 85.7 | | No/No | | | No/No | | | NS | | | 9 | |  |
| Saghazadeh *et al.*, 2017 [158] | Lead | 138/92 | 3 | SMD | 1.55 (0.20 – 2.89) | | 0.024 | -4.21 – 7.30 | | 94.2 | | No/No | | | No/No | | | NS | | | 9 | |  |
| Saghazadeh *et al.*, 2017 [158] | Lead | 722/724 | 18 | SMD | 0.60 (0.17 – 1.03) | | 0.007 | -1.34 – 2.54 | | 92.8 | | No/No | | | No/No | | | NS | | | 9 | |  |
| Mazereeuw *et al.*, 2015 [83] | Lipid Peroxidation Markers | 857/782 | 18 | SMD | 0.83 (0.56 – 1.09) | | 0.000 | -0.29 – 1.95 | | 84.0 | | Yes/No | | | No/Yes | | | III | | | 8 | |  |
| Saghazadeh *et al.*, 2017 [158] | Mercury | 1061/917 | 13 | SMD | 0.45 (0.12 – 0.78) | | 0.007 | -0.31 – 1.21 | | 25.8 | | No/No | | | No/Yes | | | NS | | | 9 | |  |
| Jafari *et al.*, 2017 [165] | Mercury | 216/184 | 4 | Hedges’ g | 1.56 (0.42 – 2.70) | | 0.007 | -2.46 – 5.58 | | 95.6 | | No/No | | | No/No | | | NS | | | 8 | |  |
| Jafari *et al.*, 2017 [165] | Mercury | 893/788 | 19 | Hedges’ g | -0.06 (-0.16 – 0.05) | | 0.293 | -0.26 – 0.14 | | 11.4 | | Yes/No | | | Yes/Yes | | | NS | | | 8 | |  |
| Jafari *et al.*, 2017 [165] | Mercury | 341/325 | 4 | Hedges’ g | 1.61 (0.83 – 2.38) | | 0.000 | -0.26 – 3.47 | | 29.5 | | Yes/No | | | No/No | | | IV | | | 8 | |  |
| Jafari *et al.*, 2017 [165] | Mercury | 414/331 | 7 | Hedges’ g | 0.17 (-0.02 – 0.36) | | 0.087 | -0.17 – 0.51 | | 14.1 | | No/No | | | No/Yes | | | NS | | | 8 | |  |
| Frustaci *et al.*, 2012 [155] | Methionine | 314/313 | 7 | SMD | -0.45 (-1.10 – 0.21) | | 0.180 | -2.68 – 1.78 | | 93.4 | | Yes/Yes | | | No/No | | | NS | | | 4 | |  |
| Frustaci *et al.*, 2012 [155] | Superoxide Dismutase | 143/121 | 5 | SMD | 0.81 (-1.36 – 2.97) | | 0.465 | -6.59 – 8.20 | | 97.7 | | No/No | | | No/No | | | NS | | | 4 | |  |
| Frustaci *et al.*, 2012 [155] | TBARS | 160/168 | 6 | SMD | 2.26 (0.65 – 3.88) | | 0.006 | -3.20 – 7.73 | | 97.1 | | No/No | | | No/No | | | NS | | | 4 | |  |
| Masi *et al.*, 2015 [164] | TGF-Beta 1 | 289/279 | 7 | Hedges’ g | 0.55 (0.25 – 0.85) | | 0.000 | -0.29 – 1.39 | | 63.2 | | No/No | | | No/Yes | | | II | | | 7 | |  |
| Main *et al.*, 2012 [161] | TGSH | 221/209 | 6 | SMD | -1.85 (-2.70 – -1.00) | | 0.000 | -4.65 – 0.95 | | 91.9 | | No/No | | | No/No | | | IV | | | 10 | |  |
| Masi *et al.*, 2015 [164] | TNF-Alpha | 331/292 | 8 | Hedges’ g | -0.23 (-0.78 – 0.32) | | 0.412 | -2.14 – 1.68 | | 90.9 | | No/Yes | | | No/No | | | NS | | | 7 | |  |
| Frustaci *et al.*, 2012 [155] | TOTAL GSH (Fasting) | 200/169 | 5 | SMD | -1.43 (-2.51 – -0.35) | | 0.009 | -5.06 – 2.20 | | 94.5 | | No/No | | | No/No | | | NS | | | 4 | |  |
| Babaknejad *et al.*, 2016 [166] | Zinc | 492/376 | 13 | SMD | -0.41 (-0.88 – 0.05) | | 0.082 | -2.23 – 1.41 | | 89.6 | | No/No | | | No/No | | | NS | | | 11 | |  |

**Abbreviations:** ADH, Antidiuretic Hormone; AMSTAR, A Measurement Tool to Assess Systematic Reviews; ANG-2I, Angiopoietin-2; BDNF, Brain-Derived Neurotrophic Factor; CAT, Catalase; CCL, Chemokine Ligands; CXCL-10, Cemokine (C-X-C motif) ligand 10; CI, Confidence Interval, CRH, Corticotropin; CRP, C-Reactive Protein; DEX, Dexamethasone; DHA, Docosahexaenoic Acid; DHEA, Dehydroepiandrosterone; DHEAS, Dehydroepiandrosterone sulfate; DPA, Docosapentaenoic Acid; EGF, Epidermal Growth Factor, EPA, Eicosapentaenoic Acid; FGF, Fibroblast Growth Factor; g, Hedge’s g; GABA, Gamma-Aminobutyric Acid; GDNF, Glial Cell Line-Derived Neurotrophic Factor; GSH, Glutathione; GSH-Px, Glutathione Peroxidase; GSSG, Glutathione Disulfide; hsCRP; High-Sensitivity C Reactive Protein; HR, Hazard Ratio; ICAM, Intercellular Adhesion Molecule 1; IL, Interleukine; INF, Interferon; MDA, Malondialdehyde; N, N of primary studies; NA, Not Available; NGF, Nerve Growth Factor; NE, No Evidence; NMDAR, N-Methyl-D-Aspartate Receptor Antibody Seropositivity; NO, Nitric Oxide; NS, Non-significant; NT, Neurotrophin; OR, Odds Ratio; OGTT, Oral Glucose Tolerance Test; PI, Prediction Interval; RR, Risk Ration; SMD, Standardized Mean Difference; SOD, Superoxide Dismutase; TAS, Total Antioxidant Status; TBARS, Thiobarbituric Acidic Reactive Substances; TGF, Transforming Growth Factor; TNF, Tumor Necrosis Factor; TSST, Laboratory Trier Social Stress Test; VCAM-1, Vascular Cell Adhesion Molecule 1; VEGF, Vascular Endothelial Growth Factor; WMD, Weighted Mean Difference; 5-HT, 5-hydroxytryptamine.

| Table S3. Peripheral biomarker derived from included within-group meta-analyses. | | | | | | | | | | | |
| --- | --- | --- | --- | --- | --- | --- | --- | --- | --- | --- | --- |
| Reference | Biomarker | N of primary studies | Effect size metric | Random effects summary effect size (95% CI) | P (by random effects) | 95% PI | I^2^ | Small-study effects/ excess statistical significance | Power criteria  Small/ Medium ES | Level of evidence^*^ | AMSTAR |
| Acute Depression |  |  |  |  |  |  |  |  |  |  |  |
| Goldsmith *et al*., 2016[62] | IL-1β | 5 | Hedges’ g | 0.14 (-1.14 – 1.43) | 0.827 | -4.15 – 4.44 | 92.7 | No/Yes | No/No | NS | 10 |
| Goldsmith *et al*., 2016[62] | IL-2 | 3 | Hedges’ g | -0.22 (-2.60 – 2.17) | 0.859 | -10.57 – 10.14 | 97.6 | No/Yes | No/No | NS | 10 |
| Goldsmith *et al*., 2016[62] | IL-4 | 3 | Hedges’ g | 1.07 (-0.48 – 2.62) | 0.176 | -5.59 – 7.74 | 94.9 | Yes/No | No/No | NS | 10 |
| Goldsmith *et al*., 2016[62] | IL-6 | 8 | Hedges’ g | -0.38 (-0.81 – 0.06) | 0.091 | -1.64 – 0.88 | 61.0 | No/No | No/No | NS | 10 |
| Goldsmith *et al*., 2016[62] | sIL-2R | 5 | Hedges’ g | -0.04 (-0.28 – 0.19) | 0.717 | -0.38 – 0.29 | 0.0 | No/No | No/Yes | NS | 10 |
| Goldsmith *et al*., 2016[62] | TNF-Alpha | 8 | Hedges’ g | -0.32 (-0.87 – 0.23) | 0.256 | -2.13 – 1.49 | 84.7 | No/No | No/No | NS | 10 |
| Depression |  |  |  |  |  |  |  |  |  |  |  |
| Nascimento *et al.*, 2015 [64] | AΒ40 | 5 | SMD | -0.09 (-0.45 – 0.26) | 0.598 | -1.15 – 0.96 | 80.9 | No/No | No/No | NS | 7 |
| Zhou *et al.*, 2018[147] | BDNF | 19 | SMD | 0.60 (0.25 – 0.95) | 0.001 | -0.91 – 2.11 | 86.8 | No/No | No/No | IV | 8 |
| Kohler *et al*., 2018 [167] | CCL-2 | 5 | Hedges’ g | -1.50 (-2.58 – -0.42) | 0.006 | -5.08 – 2.07 | 96.0 | No/No | No/No | NS | 11 |
| Kohler *et al*., 2018 [167] | CCL-3 | 4 | Hedges’ g | -0.55 (-1.38 – 0.28) | 0.191 | -3.47 – 2.36 | 93.6 | No/No | No/No | NS | 11 |
| Kohler *et al*., 2018 [167] | IFN-Gamma | 9 | Hedges’ g | 0.13 (-0.43 – 0.70) | 0.642 | -1.86 – 2.13 | 93.2 | No/Yes | No/No | NS | 11 |
| Kohler *et al*., 2018 [167] | IL-10 | 10 | Hedges’ g | -0.57 (-1.01 – -0.12) | 0.012 | -2.19 – 1.06 | 92.5 | No/No | No/No | NS | 11 |
| Kohler *et al*., 2018 [167] | IL-13 | 3 | Hedges’ g | -0.54 (-1.43 – 0.34) | 0.225 | -4.30 – 3.21 | 92.9 | No/No | No/No | NS | 11 |
| Kohler *et al*., 2018 [167] | IL-17 | 3 | Hedges’ g | -1.24 (-2.67 – 0.19) | 0.088 | -7.00 – 4.52 | 94.6 | No/No | No/No | NS | 11 |
| Kohler *et al*., 2018 [167] | IL-1RA | 4 | Hedges’ g | -0.17 (-0.48 – 0.15) | 0.305 | -1.13 – 0.80 | 64.2 | No/No | No/Yes | NS | 11 |
| Kohler *et al*., 2018 [167] | IL-1Β | 15 | Hedges’ g | -0.25 (-0.62 – 0.11) | 0.176 | -1.80 – 1.29 | 92.0 | No/Yes | No/No | NS | 11 |
| Kohler *et al*., 2018 [167] | IL-2 | 8 | Hedges’ g | -0.09 (-0.83 – 0.64) | 0.800 | -2.66 – 2.47 | 94.8 | No/Yes | No/No | NS | 11 |
| Kohler *et al*., 2018 [167] | IL-4 | 10 | Hedges’ g | 0.51 (-0.18 – 1.20) | 0.145 | -2.03 – 3.05 | 95.5 | No/No | No/No | NS | 11 |
| Kohler *et al*., 2018 [167] | IL-5 | 3 | Hedges’ g | -0.12 (-0.55 – 0.30) | 0.567 | -1.78 – 1.53 | 73.3 | No/Yes | No/No | NS | 11 |
| Kohler *et al*., 2018 [167] | IL-6 | 24 | Hedges’ g | -0.45 (-0.66 – -0.25) | 0.000 | -1.42 – 0.51 | 84.7 | No/No | No/Yes | II | 11 |
| Kohler *et al*., 2018 [167] | IL-7 | 3 | Hedges’ g | -0.19 (-0.73 – 0.35) | 0.498 | -2.40 – 2.03 | 83.0 | No/No | No/No | NS | 11 |
| Kohler *et al*., 2018 [167] | IL-8 | 7 | Hedges’ g | -0.06 (-0.31 – 0.20) | 0.668 | -0.84 – 0.73 | 76.9 | No/Yes | No/Yes | NS | 11 |
| Mazereeuw *et al.*, 2015 [83] | Lipid Peroxidation Markers | 6 | SMD | 0.71 (0.46 – 0.97) | 0.000 | 0.08 – 1.35 | 42.7 | No/No | No/Yes | II | 8 |
| Kohler *et al*., 2018 [167] | SIL-2 Receptor | 4 | Hedges’ g | -0.05 (-0.21 – 0.10) | 0.490 | -0.30 – 0.19 | 0.0 | No/No | No/Yes | NS | 11 |
| Kohler *et al*., 2018 [167] | TNF-Alpha | 23 | Hedges’ g | -0.20 (-0.37 – -0.04) | 0.015 | -0.94 – 0.54 | 80.0 | No/Yes | No/Yes | NS | 11 |
| Bipolar Disorder (Mania) |  |  |  |  |  |  |  |  |  |  |  |
| Goldsmith *et al*., 2016[62] | sIL-2R | 3 | Hedges’ g | -0.27 (-0.58 – 0.03) | 0.081 | -0.95 – 0.40 | 0.0 | No/No | No/Yes | NS | 10 |
| Goldsmith *et al*., 2016[62] | sIL-6R | 3 | Hedges’ g | 0.08 (-0.22 – 0.39) | 0.603 | -0.59 – 0.75 | 0.0 | No/No | No/Yes | NS | 10 |
| Acute psychosis |  |  |  |  |  |  |  |  |  |  |  |
| Goldsmith *et al*., 2016[62] | IFN-Gamma | 5 | Hedges’ g | -0.12 (-0.29 – 0.05) | 0.155 | -0.37 – 0.12 | 0.0 | No/No | No/Yes | NS | 10 |
| Goldsmith *et al*., 2016[62] | IL-12 | 3 | Hedges’ g | 0.31 (-0.08 – 0.71) | 0.123 | -1.04 – 1.67 | 46.9 | No/No | No/No | NS | 10 |
| Goldsmith *et al*., 2016[62] | IL-17 | 3 | Hedges’ g | -0.13 (-0.33 – 0.07) | 0.215 | -0.56 – 0.31 | 0.0 | No/No | No/Yes | NS | 10 |
| Goldsmith *et al*., 2016[62] | IL-1β | 4 | Hedges’ g | -0.30 (-0.58 – -0.02) | 0.034 | -1.01 – 0.42 | 38.7 | No/No | No/Yes | NS | 10 |
| Goldsmith *et al*., 2016[62] | IL-2 | 4 | Hedges’ g | -0.10 (-0.52 – 0.32) | 0.637 | -1.37 – 1.17 | 63.7 | No/No | No/No | NS | 10 |
| Goldsmith *et al*., 2016[62] | IL-4 | 3 | Hedges’ g | -0.29 (-0.50 – -0.09) | 0.005 | -0.74 – 0.16 | 0.0 | No/No | No/Yes | NS | 10 |
| Goldsmith *et al*., 2016[62] | IL-6 | 12 | Hedges’ g | -0.15 (-0.32 – 0.03) | 0.095 | -0.64 – 0.35 | 46.5 | No/No | No/Yes | NS | 10 |
| Goldsmith *et al*., 2016[62] | sIL-2R | 3 | Hedges’ g | 0.30 (0.00 – 0.61) | 0.054 | -0.46 – 1.06 | 8.6 | No/No | No/Yes | NS | 10 |
| Goldsmith *et al*., 2016[62] | TGF-Beta | 5 | Hedges’ g | -0.07 (-0.47 – 0.33) | 0.729 | -1.35 – 1.21 | 82.3 | No/Yes | No/No | NS | 10 |
| Goldsmith *et al*., 2016[62] | TNF-Alpha | 6 | Hedges’ g | 0.00 (-0.28 – 0.29) | 0.985 | -0.83 – 0.84 | 67.8 | No/Yes | No/Yes | NS | 10 |
| Schizophrenia |  |  |  |  |  |  |  |  |  |  |  |
| Bartoli *et al.*, 2015 [168] | Adiponectin | 29 | SMD | -0.33 (-0.56 – -0.11) | 0.004 | -1.51 – 0.84 | 89.3 | No/No | No/Yes | II | 8 |
| Brouwer *et al.*, 2013 [169] | Blood Serine | 18 | SMD | 0.29 (0.00 – 0.57) | 0.048 | -0.87 – 1.44 | 83.1 | No/No | No/Yes | NS | 7 |
| Goetz *et al.*, 2019 [170] | Ghrelin | 6 | SMD | -0.48 (-0.88 – -0.08) | 0.018 | -1.54 – 0.58 | 52.7 | No/No | No/No | NS | 9 |
| Autism |  |  |  |  |  |  |  |  |  |  |  |
| Mazereeuw *et al.*, 2015 [83] | Lipid Peroxidation Markers | 6 | SMD | 0.71 (0.46 – 0.97) | 0.000 | 0.08 – 1.35 | 42.7 | No/No | No/Yes | II | 8 |

**Abbreviations:** ADH, Antidiuretic Hormone; AMSTAR, A Measurement Tool to Assess Systematic Reviews; ANG-2I, Angiopoietin-2; BDNF, Brain-Derived Neurotrophic Factor; CAT, Catalase; CCL, Chemokine Ligands; CXCL-10, Cemokine (C-X-C motif) ligand 10; CI, Confidence Interval, CRH, Corticotropin; CRP, C-Reactive Protein; DEX, Dexamethasone; DHA, Docosahexaenoic Acid; DHEA, Dehydroepiandrosterone; DHEAS, Dehydroepiandrosterone sulfate; DPA, Docosapentaenoic Acid; EGF, Epidermal Growth Factor, EPA, Eicosapentaenoic Acid; FGF, Fibroblast Growth Factor; g, Hedge’s g; GABA, Gamma-Aminobutyric Acid; GDNF, Glial Cell Line-Derived Neurotrophic Factor; GSH, Glutathione; GSH-Px, Glutathione Peroxidase; GSSG, Glutathione Disulfide; hsCRP; High-Sensitivity C Reactive Protein; HR, Hazard Ratio; ICAM, Intercellular Adhesion Molecule 1; IL, Interleukine; INF, Interferon; MDA, Malondialdehyde; N, N of primary studies; NA, Not Available; NGF, Nerve Growth Factor; NE, No Evidence; NMDAR, N-Methyl-D-Aspartate Receptor Antibody Seropositivity; NO, Nitric Oxide; NS, Non-significant; NT, Neurotrophin; OR, Odds Ratio; OGTT, Oral Glucose Tolerance Test; PI, Prediction Interval; RR, Risk Ration; SMD, Standardized Mean Difference; SOD, Superoxide Dismutase; TAS, Total Antioxidant Status; TBARS, Thiobarbituric Acidic Reactive Substances; TGF, Transforming Growth Factor; TNF, Tumor Necrosis Factor; TSST, Laboratory Trier Social Stress Test; VCAM-1, Vascular Cell Adhesion Molecule 1; VEGF, Vascular Endothelial Growth Factor; WMD, Weighted Mean Difference; 5-HT, 5-hydroxytryptamine.

| Table S4. Qualitative methodological quality appraisal of included meta-analyses using the AMSTAR tool. | | | | | | | | | | | | |
| --- | --- | --- | --- | --- | --- | --- | --- | --- | --- | --- | --- | --- |
| Reference | **Questions** | | | | | | | | | | | **Total** |
|  | **1** | **2** | **3** | **4** | **5** | **6** | **7** | **8** | **9** | **10** | **11** |  |
| Goldsmith *et al*., 2016[62] | 1 | 1 | 1 | 1 | 0 | 1 | 1 | 1 | 1 | 1 | 1 | 10 |
| Annweiler *et al.*, 2013[128] | 1 | 1 | 1 | 1 | 1 | 1 | 1 | 1 | 1 | 1 | 1 | 11 |
| Shanthi *et al.*, 2015[129] | 0 | 1 | 1 | 0 | 0 | 1 | 1 | 1 | 1 | 1 | 1 | 8 |
| Lai *et al.,* 2017 [130] | 1 | 0 | 1 | 0 | 1 | 1 | 1 | 1 | 1 | 1 | 1 | 9 |
| Mullan *et al.,* 2018 [131] | 1 | 1 | 1 | 0 | 0 | 1 | 0 | 0 | 1 | 1 | 1 | 7 |
| Xu *et al.,* 2018[132] | 0 | 1 | 1 | 0 | 0 | 1 | 1 | 0 | 1 | 1 | 1 | 7 |
| Shi *et al.*, 2017[133] | 0 | 1 | 1 | 0 | 0 | 1 | 1 | 1 | 1 | 1 | 1 | 8 |
| Wang *et al.*, 2014[134] | 0 | 1 | 1 | 1 | 0 | 1 | 1 | 1 | 1 | 1 | 1 | 9 |
| Song *et al.*, 2011[135] | 1 | 1 | 1 | 0 | 0 | 1 | 0 | 0 | 1 | 1 | 0 | 6 |
| De Wilde *et al.*, 2017 [136] | 1 | 0 | 1 | 0 | 0 | 1 | 0 | 0 | 1 | 0 | 0 | 4 |
| Du *et al.*, 2018[137] | 1 | 1 | 1 | 0 | 0 | 1 | 0 | 0 | 1 | 1 | 1 | 7 |
| Yang *et al.*, 2017[138] | 1 | 1 | 1 | 0 | 0 | 1 | 1 | 0 | 1 | 1 | 1 | 8 |
| Li *et al.*, [139] | 0 | 1 | 1 | 1 | 0 | 1 | 1 | 1 | 1 | 1 | 1 | 9 |
| Schneider *et al.*, 1992 [140] | 0 | 0 | 0 | 0 | 1 | 0 | 0 | 0 | 0 | 0 | 0 | 1 |
| Xu *et al.*, 2016 [141] | 1 | 0 | 1 | 0 | 0 | 1 | 0 | 0 | 1 | 1 | 1 | 6 |
| Lopes da Silva *et al.*, 2014 [142] | 1 | 0 | 1 | 0 | 0 | 0 | 0 | 0 | 1 | 1 | 0 | 4 |
| Shen *et al.*, 2015 [143] | 1 | 0 | 1 | 0 | 1 | 1 | 1 | 1 | 1 | 1 | 1 | 9 |
| Ho *et al.*, 2011 [144] | 1 | 0 | 1 | 0 | 0 | 1 | 0 | 0 | 1 | 1 | 0 | 5 |
| Beydoun et al., 2014 [150] | 0 | 0 | 0 | 0 | 0 | 1 | 1 | 0 | 1 | 1 | 1 | 5 |
| Hu *et al.*, 2016[145] | 1 | 1 | 1 | 0 | 0 | 1 | 0 | 0 | 1 | 1 | 1 | 7 |
| Ma *et al.*, 2016[146] | 0 | 1 | 1 | 0 | 0 | 1 | 1 | 1 | 1 | 1 | 1 | 8 |
| Zhou *et al.*, 2018[147] | 1 | 0 | 1 | 0 | 1 | 1 | 1 | 1 | 1 | 1 | 0 | 8 |
| Schrag *et al.*, 2013 [148] | 1 | 0 | 1 | 0 | 0 | 1 | 0 | 0 | 1 | 1 | 1 | 6 |
| Du et al., 2017[149] | 1 | 1 | 1 | 0 | 0 | 1 | 1 | 1 | 1 | 1 | 0 | 8 |
| Squitti *et al.*, 2014[151] | 1 | 0 | 1 | 0 | 0 | 1 | 1 | 1 | 1 | 1 | 0 | 7 |
| Liu *et al.*, 2018 [152] | 1 | 1 | 1 | 0 | 0 | 1 | 1 | 1 | 1 | 1 | 1 | 9 |
| Du *et al.*, 2016[153] | 0 | 1 | 1 | 0 | 0 | 0 | 0 | 0 | 1 | 1 | 0 | 4 |
| Ventriglia *et al.*, 2015[154] | 1 | 0 | 1 | 0 | 1 | 1 | 1 | 1 | 1 | 1 | 1 | 9 |
| Frustaci *et al.*, 2012 [155] | 0 | 0 | 1 | 0 | 0 | 1 | 0 | 0 | 1 | 1 | 0 | 4 |
| Wang et al., 2016 [156] | 0 | 1 | 1 | 1 | 0 | 1 | 1 | 1 | 1 | 1 | 1 | 9 |
| Gabriele *et al.*, 2014[157] | 1 | 0 | 1 | 0 | 0 | 1 | 0 | 0 | 1 | 1 | 1 | 6 |
| Saghazadeh *et al.*, 2017 [158] | 1 | 1 | 1 | 0 | 1 | 1 | 1 | 1 | 0 | 1 | 1 | 9 |
| Mazahery *et al.*, 2017 [159] | 1 | 0 | 1 | 0 | 0 | 1 | 1 | 1 | 1 | 1 | 1 | 8 |
| Saghazadeh *et al.*, 2017 [160] | 0 | 0 | 0 | 0 | 0 | 1 | 1 | 1 | 1 | 1 | 1 | 6 |
| Jafari *et al.*, 2017 [165] | 0 | 1 | 1 | 0 | 0 | 1 | 1 | 1 | 1 | 1 | 1 | 8 |
| Masi *et al.*, 2015 [164] | 0 | 1 | 1 | 1 | 0 | 1 | 0 | 0 | 1 | 1 | 1 | 7 |
| Main *et al.*, 2012 [161] | 0 | 1 | 1 | 1 | 1 | 1 | 1 | 1 | 1 | 1 | 1 | 10 |
| Tseng *et al.*, 2018 [162] | 1 | 1 | 1 | 1 | 1 | 1 | 1 | 1 | 1 | 1 | 1 | 11 |
| Zheng *et al.*, 2016 [163] | 0 | 1 | 1 | 0 | 0 | 1 | 1 | 1 | 1 | 1 | 0 | 7 |
| Belvederi Murri *et al.*, 2016 [96] | 1 | 0 | 1 | 0 | 0 | 1 | 1 | 1 | 1 | 1 | 1 | 8 |
| Fernandes *et al.*, 2015[101] | 1 | 1 | 1 | 0 | 1 | 1 | 0 | 0 | 1 | 1 | 1 | 8 |
| Fernandes *et al.*, 2016 [102] | 0 | 1 | 1 | 0 | 1 | 1 | 0 | 0 | 1 | 1 | 1 | 7 |
| Zorn *et al.*, 2016[73] | 0 | 0 | 0 | 0 | 1 | 1 | 0 | 0 | 1 | 1 | 0 | 4 |
| Tu *et al.*, 2016 [81] | 0 | 0 | 0 | 0 | 1 | 1 | 1 | 1 | 1 | 1 | 0 | 6 |
| Pearlman, 2014 [87] | 1 | 0 | 1 | 1 | 0 | 1 | 0 | 0 | 1 | 1 | 1 | 7 |
| Tseng *et al.*, 2016 [99] | 0 | 1 | 1 | 1 | 0 | 1 | 0 | 0 | 1 | 1 | 1 | 7 |
| Looney *et al.*, 1997 [97] | 0 | 0 | 0 | 0 | 0 | 1 | 0 | 0 | 0 | 0 | 0 | 1 |
| Rao *et al.*, 2017 [98] | 1 | 1 | 1 | 1 | 1 | 1 | 0 | 0 | 1 | 1 | 1 | 9 |
| Rutigliano *et al.*, 2016 [92] | 1 | 1 | 1 | 1 | 1 | 1 | 1 | 1 | 1 | 1 | 1 | 11 |
| Bartoli *et al.*, 2016 [100] | 1 | 1 | 1 | 0 | 0 | 0 | 0 | 0 | 1 | 1 | 1 | 6 |
| Fernandes *et al.*, 2016 [103] | 1 | 1 | 1 | 0 | 0 | 1 | 0 | 0 | 1 | 1 | 1 | 7 |
| Babaknejad *et al.*, 2016 [166] | 1 | 1 | 1 | 1 | 1 | 1 | 1 | 1 | 1 | 1 | 1 | 11 |
| Nascimento *et al.*, 2015 [64] | 1 | 1 | 1 | 0 | 0 | 1 | 0 | 0 | 1 | 1 | 1 | 7 |
| Ogyu *et al.*, 2018 [63] | 0 | 1 | 1 | 1 | 0 | 1 | 1 | 1 | 1 | 1 | 1 | 9 |
| Lin *et al.*, 2010 [66] | 0 | 0 | 0 | 1 | 0 | 1 | 0 | 0 | 1 | 1 | 1 | 5 |
| Mokhtari *et al.*, 2013[67] | 0 | 0 | 0 | 0 | 0 | 1 | 0 | 0 | 1 | 0 | 0 | 2 |
| Petridou *et al.*, 2016 [68] | 1 | 0 | 1 | 0 | 0 | 1 | 1 | 1 | 1 | 1 | 0 | 7 |
| Molendijk *et al.*, 2014 [69] | 0 | 1 | 1 | 1 | 1 | 1 | 1 | 1 | 1 | 1 | 1 | 10 |
| Ni et al., [71] | 1 | 0 | 1 | 0 | 1 | 1 | 1 | 0 | 1 | 1 | 1 | 8 |
| Ciufolini *et al.*, 2014 [72] | 1 | 0 | 1 | 0 | 0 | 1 | 0 | 0 | 1 | 0 | 0 | 4 |
| Haapakoski *et al.*, 2015 [74] | 0 | 1 | 1 | 0 | 1 | 1 | 1 | 1 | 1 | 1 | 0 | 8 |
| Zhu *et al.*, 2015 [75] | 0 | 1 | 1 | 0 | 0 | 1 | 1 | 0 | 1 | 1 | 1 | 7 |
| Wu *et al.*, 2016 [76] | 0 | 0 | 1 | 0 | 1 | 0 | 1 | 1 | 1 | 0 | 0 | 5 |
| Romeo *et al.*, 2018 [78] | 0 | 0 | 1 | 1 | 0 | 1 | 0 | 0 | 1 | 1 | 1 | 6 |
| Lin *et al.*, 2015 [79] | 0 | 1 | 1 | 0 | 1 | 1 | 0 | 0 | 1 | 1 | 1 | 7 |
| Inoshita *et al.*, 2018 [80] | 0 | 0 | 1 | 0 | 0 | 1 | 0 | 0 | 1 | 1 | 1 | 5 |
| Kohler et al., 2017 [70] | 1 | 1 | 1 | 1 | 1 | 1 | 1 | 1 | 1 | 1 | 1 | 11 |
| Persons et al., 2016 [82] | 0 | 0 | 0 | 0 | 0 | 1 | 0 | 0 | 1 | 1 | 1 | 4 |
| Ogawa *et al*., 2014 [84] | 0 | 0 | 0 | 0 | 1 | 1 | 1 | 1 | 1 | 1 | 1 | 7 |
| You *et al.*, 2018 [85] | 0 | 1 | 1 | 0 | 0 | 1 | 1 | 1 | 1 | 1 | 1 | 8 |
| Kohler *et al*., 2018 [167] | 1 | 1 | 1 | 1 | 1 | 1 | 1 | 1 | 1 | 1 | 1 | 11 |
| Chen *et al.*, 2015 [86] | 0 | 1 | 0 | 0 | 1 | 1 | 0 | 0 | 1 | 1 | 0 | 5 |
| Mazza *et al.*, 2018 [88] | 1 | 1 | 1 | 1 | 1 | 1 | 0 | 0 | 1 | 0 | 0 | 7 |
| Shin *et al.*, 2008[90] | 0 | 0 | 1 | 1 | 0 | 1 | 0 | 0 | 1 | 0 | 1 | 5 |
| Bartoli *et al.*, 2018 [91] | 0 | 1 | 1 | 1 | 0 | 1 | 1 | 1 | 1 | 1 | 1 | 9 |
| Tseng *et al.*, 2015 [93] | 1 | 1 | 0 | 0 | 0 | 1 | 1 | 1 | 1 | 1 | 0 | 7 |
| Anglin *et al.*, 2013 [94] | 1 | 1 | 1 | 1 | 0 | 1 | 1 | 1 | 1 | 1 | 1 | 10 |
| Swardfager *et al.*, 2013 [95] | 1 | 1 | 1 | 0 | 1 | 1 | 1 | 1 | 1 | 1 | 1 | 10 |
| Firth et al., 2018 [125] | 0 | 1 | 1 | 1 | 0 | 1 | 0 | 0 | 1 | 1 | 1 | 7 |
| Chaumette *et al.*, 2016 [9] | 1 | 0 | 0 | 0 | 0 | 1 | 0 | 0 | 1 | 1 | 0 | 4 |
| Berger *et al.*, 2016 [126] | 0 | 1 | 1 | 1 | 0 | 1 | 1 | 1 | 0 | 0 | 1 | 7 |
| Pillinger *et al.*, 2017 [127] | 0 | 1 | 1 | 0 | 0 | 1 | 1 | 0 | 1 | 1 | 1 | 7 |
| Aleksovska *et al.*, 2014 [105] | 0 | 1 | 1 | 0 | 0 | 1 | 1 | 1 | 1 | 1 | 1 | 8 |
| Lachance *et al.*, 2014 [106] | 1 | 1 | 1 | 0 | 0 | 1 | 0 | 0 | 1 | 1 | 1 | 7 |
| Hoen *et al.*, 2013 [107] | 0 | 1 | 1 | 1 | 0 | 1 | 1 | 1 | 1 | 1 | 1 | 9 |
| Fernandes et al., 2015 [108] | 1 | 1 | 1 | 0 | 0 | 1 | 1 | 1 | 1 | 1 | 1 | 9 |
| Flatow *et al.*, 2013 [109] | 0 | 1 | 1 | 0 | 1 | 1 | 1 | 1 | 1 | 0 | 1 | 8 |
| Fernandes *et al.*, 2016 [110] | 1 | 1 | 1 | 0 | 0 | 1 | 0 | 0 | 1 | 1 | 1 | 7 |
| Greenhalgh *et al.*, 2016 [111] | 1 | 1 | 1 | 0 | 0 | 0 | 0 | 0 | 1 | 1 | 1 | 6 |
| Wang *et al.*, 2016 [112] | 1 | 1 | 1 | 0 | 1 | 1 | 0 | 0 | 1 | 1 | 1 | 8 |
| Song *et al.*, 2014 [113] | 0 | 0 | 1 | 0 | 1 | 1 | 0 | 0 | 1 | 1 | 0 | 5 |
| Nishi *et al.*, 2014 [114] | 1 | 1 | 0 | 0 | 0 | 1 | 0 | 1 | 0 | 1 | 0 | 5 |
| Guo *et al.*, 2015 [115] | 0 | 1 | 1 | 0 | 0 | 0 | 0 | 0 | 1 | 1 | 0 | 4 |
| Plitman *et al.*, 2017 [117] | 0 | 1 | 1 | 1 | 0 | 1 | 1 | 0 | 1 | 1 | 1 | 8 |
| Stubbs *et al.*, 2016 [118] | 0 | 1 | 1 | 0 | 1 | 1 | 1 | 1 | 1 | 1 | 1 | 9 |
| Qin *et al.*, 2017 [119] | 0 | 1 | 1 | 0 | 1 | 1 | 0 | 0 | 1 | 1 | 1 | 7 |
| Maia-de-Oliveira *et al.*, 2012 [120] | 1 | 1 | 1 | 0 | 0 | 1 | 0 | 0 | 1 | 1 | 0 | 6 |
| Valipour *et al.*, 2014 [123] | 0 | 0 | 1 | 0 | 0 | 1 | 1 | 1 | 1 | 1 | 0 | 6 |
| Tomioka *et al.*, 2018 [122] | 0 | 1 | 1 | 0 | 0 | 0 | 0 | 0 | 1 | 1 | 1 | 5 |
| Misiak *et al.*, 2018 [121] | 1 | 0 | 1 | 0 | 0 | 1 | 1 | 1 | 1 | 1 | 1 | 8 |
| Brouwer *et al.*, 2013 [169] | 1 | 1 | 1 | 0 | 0 | 1 | 0 | 0 | 1 | 1 | 1 | 7 |
| Bartoli *et al.*, 2015 [168] | 1 | 1 | 1 | 1 | 0 | 1 | 1 | 1 | 1 | 0 | 0 | 8 |
| Goetz *et al.*, 2019 [170] | 1 | 1 | 1 | 0 | 0 | 1 | 1 | 1 | 1 | 1 | 1 | 9 |
| Mazereeuw *et al.*, 2015 [83] | 1 | 1 | 1 | 0 | 0 | 1 | 1 | 1 | 1 | 1 | 0 | 8 |
| Schrag et al., 2013[148] | 1 | 0 | 1 | 0 | 0 | 1 | 0 | 0 | 1 | 1 | 1 | 6 |
| Carvalho *et al.*, 2014 [89] | 1 | 1 | 1 | 0 | 1 | 1 | 0 | 0 | 1 | 1 | 1 | 8 |
| Bender *et al.*, 2017 [77] | 0 | 1 | 1 | 1 | 0 | 1 | 0 | 0 | 1 | 1 | 1 | 7 |
| Fang *et al.*, 2018 [116] | 0 | 1 | 1 | 0 | 0 | 1 | 0 | 0 | 1 | 0 | 1 | 5 |
| Cao *et al.*, 2018 [65] | 1 | 1 | 1 | 0 | 0 | 1 | 1 | 1 | 1 | 1 | 1 | 9 |
| Joe *et al.*, 2018 [124] | 0 | 1 | 1 | 0 | 0 | 1 | 1 | 1 | 1 | 1 | 0 | 7 |
| Salagre *et al.*, 2017 [104] | 1 | 1 | 1 | 0 | 0 | 1 | 1 | 1 | 1 | 1 | 1 | 9 |
| Abbreviations: 0, No; 1, Yes; AMSTAR, A Measurement Tool to Assess Systematic Reviews. | | | | | | | | | | | | |

**Supplementary References**

1. Ahmed AO, Mantini AM, Fridberg DJ, Buckley PF. Brain-derived neurotrophic factor (BDNF) and neurocognitive deficits in people with schizophrenia: a meta-analysis. Psychiatry research 2015; 226**:** 1-13.

2. Annweiler C, Montero-Odasso M, Llewellyn DJ, Richard-Devantoy S, Duque G, Beauchet O. Meta-analysis of memory and executive dysfunctions in relation to vitamin D. Journal of Alzheimer's disease : JAD 2013; 37**:** 147-171.

3. Anstey KJ, Lipnicki DM, Low LF. Cholesterol as a risk factor for dementia and cognitive decline: a systematic review of prospective studies with meta-analysis. The American journal of geriatric psychiatry : official journal of the American Association for Geriatric Psychiatry 2008; 16**:** 343-354.

4. Boggero IA, Hostinar CE, Haak EA, Murphy MLM, Segerstrom SC. Psychosocial functioning and the cortisol awakening response: Meta-analysis, P-curve analysis, and evaluation of the evidential value in existing studies. Biological psychology 2017; 129**:** 207-230.

5. Capuzzi E, Bartoli F, Crocamo C, Clerici M, Carra G. Acute variations of cytokine levels after antipsychotic treatment in drug-naive subjects with a first-episode psychosis: A meta-analysis. Neuroscience and biobehavioral reviews 2017; 77**:** 122-128.

6. Carvalho AF, Kohler CA, McIntyre RS, Knochel C, Brunoni AR, Thase ME *et al.* Peripheral vascular endothelial growth factor as a novel depression biomarker: A meta-analysis. Psychoneuroendocrinology 2015; 62**:** 18-26.

7. Chan MK, Krebs MO, Cox D, Guest PC, Yolken RH, Rahmoune H *et al.* Development of a blood-based molecular biomarker test for identification of schizophrenia before disease onset. Translational psychiatry 2015; 5**:** e601.

8. Chan MK, Cooper JD, Bot M, Steiner J, Penninx BW, Bahn S. Identification of an Immune-Neuroendocrine Biomarker Panel for Detection of Depression: A Joint Effects Statistical Approach. Neuroendocrinology 2016; 103**:** 693-710.

9. Chaumette B, Kebir O, Mam-Lam-Fook C, Morvan Y, Bourgin J, Godsil BP *et al.* Salivary cortisol in early psychosis: New findings and meta-analysis. Psychoneuroendocrinology 2016; 63**:** 262-270.

10. Cooper C, Sommerlad A, Lyketsos CG, Livingston G. Modifiable predictors of dementia in mild cognitive impairment: a systematic review and meta-analysis. The American journal of psychiatry 2015; 172**:** 323-334.

11. Czapski GA, Maruszak A, Styczynska M, Zekanowski C, Safranow K, Strosznajder JB. Association between plasma biomarkers, CDK5 polymorphism and the risk of Alzheimer's disease. Acta neurobiologiae experimentalis 2012; 72**:** 397-411.

12. Dargel AA, Godin O, Kapczinski F, Kupfer DJ, Leboyer M. C-reactive protein alterations in bipolar disorder: a meta-analysis. The Journal of clinical psychiatry 2015; 76**:** 142-150.

13. Deng W, Cheung ST, Tsao SW, Wang XM, Tiwari AF. Telomerase activity and its association with psychological stress, mental disorders, lifestyle factors and interventions: A systematic review. Psychoneuroendocrinology 2016; 64**:** 150-163.

14. Dong Y, Chen X, Liu Y, Shu Y, Chen T, Xu L *et al.* Do low-serum vitamin E levels increase the risk of Alzheimer disease in older people? Evidence from a meta-analysis of case-control studies. International journal of geriatric psychiatry 2018; 33**:** e257-e263.

15. Dowlati Y, Herrmann N, Swardfager W, Liu H, Sham L, Reim EK *et al.* A meta-analysis of cytokines in major depression. Biological psychiatry 2010; 67**:** 446-457.

16. Ellis PM, Salmond C. Is platelet imipramine binding reduced in depression? A meta-analysis. Biological psychiatry 1994; 36**:** 292-299.

17. Ezeoke A, Mellor A, Buckley P, Miller B. A systematic, quantitative review of blood autoantibodies in schizophrenia. Schizophrenia research 2013; 150**:** 245-251.

18. Fernandes BS, Gama CS, Cereser KM, Yatham LN, Fries GR, Colpo G *et al.* Brain-derived neurotrophic factor as a state-marker of mood episodes in bipolar disorders: a systematic review and meta-regression analysis. Journal of psychiatric research 2011; 45**:** 995-1004.

19. Fischer S, Cleare A. HPA axis functioning as a predictor of psychotherapy response in patients with depression and anxiety disorders - A systematic review and meta-analysis. Psychoneuroendocrinology 2015; 61**:** 23-24.

20. Fischer S, Strawbridge R, Herane Vives A, Cleare AJ. Cortisol as a predictor of psychological therapy response in depressive disorders: systematic review and meta-analysis. The British journal of psychiatry : the journal of mental science 2016.

21. Fraguas D, Diaz-Caneja CM, Rodriguez-Quiroga A, Arango C. Oxidative Stress and Inflammation in Early Onset First Episode Psychosis: A Systematic Review and Meta-Analysis. The international journal of neuropsychopharmacology 2017; 20**:** 435-444.

22. Gowda U, Mutowo MP, Smith BJ, Wluka AE, Renzaho AM. Vitamin D supplementation to reduce depression in adults: meta-analysis of randomized controlled trials. Nutrition (Burbank, Los Angeles County, Calif) 2015; 31**:** 421-429.

23. Gsell W, Strein I, Krause U, Riederer P. Neurochemical abnormalities in Alzheimer's disease and Parkinson's disease--a comparative review. Journal of neural transmission Supplementum 1997; 51**:** 145-159.

24. Gutierrez-Fernandez J, Luna Del Castillo Jde D, Mananes-Gonzalez S, Carrillo-Avila JA, Gutierrez B, Cervilla JA *et al.* Different presence of Chlamydia pneumoniae, herpes simplex virus type 1, human herpes virus 6, and Toxoplasma gondii in schizophrenia: meta-analysis and analytical study. Neuropsychiatric disease and treatment 2015; 11**:** 843-852.

25. Ju SY, Lee YJ, Jeong SN. Serum 25-hydroxyvitamin D levels and the risk of depression: a systematic review and meta-analysis. The journal of nutrition, health & aging 2013; 17**:** 447-455.

26. Kloiber S, Ripke S, Kohli MA, Reppermund S, Salyakina D, Uher R *et al.* Resistance to antidepressant treatment is associated with polymorphisms in the leptin gene, decreased leptin mRNA expression, and decreased leptin serum levels. European neuropsychopharmacology : the journal of the European College of Neuropsychopharmacology 2013; 23**:** 653-662.

27. Lin PY. State-dependent decrease in levels of brain-derived neurotrophic factor in bipolar disorder: a meta-analytic study. Neuroscience letters 2009; 466**:** 139-143.

28. Martinez-Cengotitabengoa M, Carrascon L, O'Brien JT, Diaz-Gutierrez MJ, Bermudez-Ampudia C, Sanada K *et al.* Peripheral Inflammatory Parameters in Late-Life Depression: A Systematic Review. International journal of molecular sciences 2016; 17.

29. McGuinness B, O'Hare J, Craig D, Bullock R, Malouf R, Passmore P. Statins for the treatment of dementia. The Cochrane database of systematic reviews 2010**:** Cd007514.

30. Miller BJ, Buckley P, Seabolt W, Mellor A, Kirkpatrick B. Meta-analysis of cytokine alterations in schizophrenia: clinical status and antipsychotic effects. Biological psychiatry 2011; 70**:** 663-671.

31. Miller BJ, Culpepper N, Rapaport MH. C-reactive protein levels in schizophrenia: a review and meta-analysis. Clinical schizophrenia & related psychoses 2014; 7**:** 223-230.

32. Noonan K, Carey LM, Crewther SG. Meta-analyses indicate associations between neuroendocrine activation, deactivation in neurotrophic and neuroimaging markers in depression after stroke. Journal of stroke and cerebrovascular diseases : the official journal of National Stroke Association 2013; 22**:** e124-135.

33. Pascoe MC, Thompson DR, Jenkins ZM, Ski CF. Mindfulness mediates the physiological markers of stress: Systematic review and meta-analysis. Journal of psychiatric research 2017; 95**:** 156-178.

34. Perry BI, McIntosh G, Weich S, Singh S, Rees K. The association between first-episode psychosis and abnormal glycaemic control: systematic review and meta-analysis. The lancet Psychiatry 2016; 3**:** 1049-1058.

35. Ritchie C, Smailagic N, Noel-Storr AH, Takwoingi Y, Flicker L, Mason SE *et al.* Plasma and cerebrospinal fluid amyloid beta for the diagnosis of Alzheimer's disease dementia and other dementias in people with mild cognitive impairment (MCI). The Cochrane database of systematic reviews 2014**:** Cd008782.

36. Rocha RB, Dondossola ER, Grande AJ, Colonetti T, Ceretta LB, Passos IC *et al.* Increased BDNF levels after electroconvulsive therapy in patients with major depressive disorder: A meta-analysis study. Journal of psychiatric research 2016; 83**:** 47-53.

37. Rosenblat JD, Gregory JM, Carvalho AF, McIntyre RS. Depression and Disturbed Bone Metabolism: A Narrative Review of the Epidemiological Findings and Postulated Mechanisms. Current molecular medicine 2016; 16**:** 165-178.

38. Ruhe HG, Mason NS, Schene AH. Mood is indirectly related to serotonin, norepinephrine and dopamine levels in humans: a meta-analysis of monoamine depletion studies. Molecular psychiatry 2007; 12**:** 331-359.

39. Sarris J, Murphy J, Mischoulon D, Papakostas GI, Fava M, Berk M *et al.* Adjunctive Nutraceuticals for Depression: A Systematic Review and Meta-Analyses. The American journal of psychiatry 2016; 173**:** 575-587.

40. Schroeter ML, Abdul-Khaliq H, Krebs M, Diefenbacher A, Blasig IE. Neuron-specific enolase is unaltered whereas S100B is elevated in serum of patients with schizophrenia--original research and meta-analysis. Psychiatry research 2009; 167**:** 66-72.

41. Schumberg K, Polyakova M, Steiner J, Schroeter ML. Serum S100B Is Related to Illness Duration and Clinical Symptoms in Schizophrenia-A Meta-Regression Analysis. Frontiers in cellular neuroscience 2016; 10**:** 46.

42. Squitti R. Copper subtype of Alzheimer's disease (AD): meta-analyses, genetic studies and predictive value of non-ceruloplasmim copper in mild cognitive impairment conversion to full AD. Journal of trace elements in medicine and biology : organ of the Society for Minerals and Trace Elements (GMS) 2014; 28**:** 482-485.

43. Tuckwell HC, Koziol JA. On the concentration of 5-hydroxyindoleacetic acid in schizophrenia: a meta-analysis. Psychiatry research 1996; 59**:** 239-244.

44. Upthegrove R, Manzanares-Teson N, Barnes NM. Cytokine function in medication-naive first episode psychosis: a systematic review and meta-analysis. Schizophrenia research 2014; 155**:** 101-108.

45. Ventriglia M, Bucossi S, Panetta V, Squitti R. Copper in Alzheimer's disease: a meta-analysis of serum, plasma, and cerebrospinal fluid studies. Journal of Alzheimer's disease : JAD 2012; 30**:** 981-984.

46. Wang B, Zhong Y, Yan H, Cui L. Meta-analysis of plasma homocysteine content and cognitive function in elderly patients with Alzheimer's disease and vascular dementia. International journal of clinical and experimental medicine 2014; 7**:** 5118-5123.

47. Wang Y, Sheng Q, Hou X, Wang B, Zhao W, Yan S *et al.* Thyrotropin and Alzheimer's Disease Risk in the Elderly: a Systematic Review and Meta-Analysis. Molecular neurobiology 2016; 53**:** 1229-1236.

48. Wiedlocha M, Marcinowicz P, Krupa R, Janoska-Jazdzik M, Janus M, Debowska W *et al.* Effect of antidepressant treatment on peripheral inflammation markers - A meta-analysis. Progress in neuro-psychopharmacology & biological psychiatry 2018; 80**:** 217-226.

49. Williams MD, Harris R, Dayan CM, Evans J, Gallacher J, Ben-Shlomo Y. Thyroid function and the natural history of depression: findings from the Caerphilly Prospective Study (CaPS) and a meta-analysis. Clinical endocrinology 2009; 70**:** 484-492.

50. Yoshida T, Ishikawa M, Niitsu T, Nakazato M, Watanabe H, Shiraishi T *et al.* Decreased serum levels of mature brain-derived neurotrophic factor (BDNF), but not its precursor proBDNF, in patients with major depressive disorder. PloS one 2012; 7**:** e42676.

51. Yuan H, Ling K, Du X, Ge P, Wu S, Wang X. The association of three BACE1 gene polymorphisms (exon5 C/G, intron 5 T/G and 3'UTR T/A) with sporadic Alzheimer's disease susceptibility: a meta-analysis. International journal of clinical and experimental medicine 2015; 8**:** 12264-12274.

52. Zhang J, Song T, Liang H, Lian J, Zhang G, Gong H. Interleukin-18 -137 G/C and -607 C/A polymorphisms and Alzheimer's disease risk: a meta-analysis. Neurological sciences : official journal of the Italian Neurological Society and of the Italian Society of Clinical Neurophysiology 2016; 37**:** 921-927.

53. Zhou C, Zhong J, Zou B, Fang L, Chen J, Deng X *et al.* Meta-analyses of comparative efficacy of antidepressant medications on peripheral BDNF concentration in patients with depression. PloS one 2017; 12**:** e0172270.

54. Ellul P, Boyer L, Groc L, Leboyer M, Fond G. Interleukin-1 beta-targeted treatment strategies in inflammatory depression: toward personalized care. Acta psychiatrica Scandinavica 2016; 134**:** 469-484.

55. Eyre HA, Air T, Pradhan A, Johnston J, Lavretsky H, Stuart MJ *et al.* A meta-analysis of chemokines in major depression. Progress in neuro-psychopharmacology & biological psychiatry 2016; 68**:** 1-8.

56. Jiang M, Qin P, Yang X. Comorbidity between depression and asthma via immune-inflammatory pathways: a meta-analysis. Journal of affective disorders 2014; 166**:** 22-29.

57. Hiles SA, Baker AL, de Malmanche T, Attia J. A meta-analysis of differences in IL-6 and IL-10 between people with and without depression: exploring the causes of heterogeneity. Brain, behavior, and immunity 2012; 26**:** 1180-1188.

58. Liu Y, Ho RC, Mak A. Interleukin (IL)-6, tumour necrosis factor alpha (TNF-alpha) and soluble interleukin-2 receptors (sIL-2R) are elevated in patients with major depressive disorder: a meta-analysis and meta-regression. Journal of affective disorders 2012; 139**:** 230-239.

59. Hannestad J, DellaGioia N, Bloch M. The effect of antidepressant medication treatment on serum levels of inflammatory cytokines: a meta-analysis. Neuropsychopharmacology : official publication of the American College of Neuropsychopharmacology 2011; 36**:** 2452-2459.

60. Munkholm K, Brauner JV, Kessing LV, Vinberg M. Cytokines in bipolar disorder vs. healthy control subjects: a systematic review and meta-analysis. Journal of psychiatric research 2013; 47**:** 1119-1133.

61. Modabbernia A, Taslimi S, Brietzke E, Ashrafi M. Cytokine alterations in bipolar disorder: a meta-analysis of 30 studies. Biological psychiatry 2013; 74**:** 15-25.

62. Goldsmith DR, Rapaport MH, Miller BJ. A meta-analysis of blood cytokine network alterations in psychiatric patients: comparisons between schizophrenia, bipolar disorder and depression. Molecular psychiatry 2016; 21**:** 1696-1709.

63. Ogyu K, Kubo K, Noda Y, Iwata Y, Tsugawa S, Omura Y *et al.* Kynurenine pathway in depression: A systematic review and meta-analysis. Neuroscience and biobehavioral reviews 2018; 90**:** 16-25.

64. Nascimento KK, Silva KP, Malloy-Diniz LF, Butters MA, Diniz BS. Plasma and cerebrospinal fluid amyloid-beta levels in late-life depression: A systematic review and meta-analysis. Journal of psychiatric research 2015; 69**:** 35-41.

65. Cao B, Chen Y, Brietzke E, Cha D, Shaukat A, Pan Z *et al.* Leptin and adiponectin levels in major depressive disorder: A systematic review and meta-analysis. Journal of affective disorders 2018; 238**:** 101-110.

66. Lin PY, Huang SY, Su KP. A meta-analytic review of polyunsaturated fatty acid compositions in patients with depression. Biological psychiatry 2010; 68**:** 140-147.

67. Mokhtari M, Arfken C, Boutros N. The DEX/CRH test for major depression: a potentially useful diagnostic test. Psychiatry research 2013; 208**:** 131-139.

68. Petridou ET, Kousoulis AA, Michelakos T, Papathoma P, Dessypris N, Papadopoulos FC *et al.* Folate and B12 serum levels in association with depression in the aged: a systematic review and meta-analysis. Aging & mental health 2016; 20**:** 965-973.

69. Molendijk ML, Spinhoven P, Polak M, Bus BA, Penninx BW, Elzinga BM. Serum BDNF concentrations as peripheral manifestations of depression: evidence from a systematic review and meta-analyses on 179 associations (N=9484). Molecular psychiatry 2014; 19**:** 791-800.

70. Kohler CA, Freitas TH, Maes M, de Andrade NQ, Liu CS, Fernandes BS *et al.* Peripheral cytokine and chemokine alterations in depression: a meta-analysis of 82 studies. Acta psychiatrica Scandinavica 2017; 135**:** 373-387.

71. Ni M, You Y, Chen J, Zhang L. Copper in depressive disorder: A systematic review and meta-analysis of observational studies. Psychiatry research 2018; 267**:** 506-515.

72. Ciufolini S, Dazzan P, Kempton MJ, Pariante C, Mondelli V. HPA axis response to social stress is attenuated in schizophrenia but normal in depression: evidence from a meta-analysis of existing studies. Neuroscience and biobehavioral reviews 2014; 47**:** 359-368.

73. Zorn JV, Schur RR, Boks MP, Kahn RS, Joels M, Vinkers CH. Cortisol stress reactivity across psychiatric disorders: A systematic review and meta-analysis. Psychoneuroendocrinology 2016; 77**:** 25-36.

74. Haapakoski R, Mathieu J, Ebmeier KP, Alenius H, Kivimaki M. Cumulative meta-analysis of interleukins 6 and 1beta, tumour necrosis factor alpha and C-reactive protein in patients with major depressive disorder. Brain, behavior, and immunity 2015; 49**:** 206-215.

75. Zhu G, Yin Y, Xiao CL, Mao RJ, Shi BH, Jie Y *et al.* Serum DHEAS levels are associated with the development of depression. Psychiatry research 2015; 229**:** 447-453.

76. Wu CK, Tseng PT, Chen YW, Tu KY, Lin PY. Significantly higher peripheral fibroblast growth factor-2 levels in patients with major depressive disorder: A preliminary meta-analysis under MOOSE guidelines. Medicine 2016; 95**:** e4563.

77. Bender A, Hagan KE, Kingston N. The association of folate and depression: A meta-analysis. Journal of psychiatric research 2017; 95**:** 9-18.

78. Romeo B, Choucha W, Fossati P, Rotge JY. Meta-analysis of central and peripheral gamma-aminobutyric acid levels in patients with unipolar and bipolar depression. Journal of psychiatry & neuroscience : JPN 2018; 43**:** 58-66.

79. Lin PY, Tseng PT. Decreased glial cell line-derived neurotrophic factor levels in patients with depression: a meta-analytic study. Journal of psychiatric research 2015; 63**:** 20-27.

80. Inoshita M, Umehara H, Watanabe SY, Nakataki M, Kinoshita M, Tomioka Y *et al.* Elevated peripheral blood glutamate levels in major depressive disorder. Neuropsychiatric disease and treatment 2018; 14**:** 945-953.

81. Tu KY, Wu MK, Chen YW, Lin PY, Wang HY, Wu CK *et al.* Significantly Higher Peripheral Insulin-Like Growth Factor-1 Levels in Patients With Major Depressive Disorder or Bipolar Disorder Than in Healthy Controls: A Meta-Analysis and Review Under Guideline of PRISMA. Medicine 2016; 95**:** e2411.

82. Persons JE, Fiedorowicz JG. Depression and serum low-density lipoprotein: A systematic review and meta-analysis. Journal of affective disorders 2016; 206**:** 55-67.

83. Mazereeuw G, Herrmann N, Andreazza AC, Khan MM, Lanctot KL. A meta-analysis of lipid peroxidation markers in major depression. Neuropsychiatric disease and treatment 2015; 11**:** 2479-2491.

84. Ogawa S, Fujii T, Koga N, Hori H, Teraishi T, Hattori K *et al.* Plasma L-tryptophan concentration in major depressive disorder: new data and meta-analysis. The Journal of clinical psychiatry 2014; 75**:** e906-915.

85. You HJ, Cho SE, Kang SG, Cho SJ, Na KS. Decreased serum magnesium levels in depression: a systematic review and meta-analysis. Nordic journal of psychiatry 2018; 72**:** 534-541.

86. Chen YW, Lin PY, Tu KY, Cheng YS, Wu CK, Tseng PT. Significantly lower nerve growth factor levels in patients with major depressive disorder than in healthy subjects: a meta-analysis and systematic review. Neuropsychiatric disease and treatment 2015; 11**:** 925-933.

87. Pearlman DM, Najjar S. Meta-analysis of the association between N-methyl-d-aspartate receptor antibodies and schizophrenia, schizoaffective disorder, bipolar disorder, and major depressive disorder. Schizophrenia research 2014; 157**:** 249-258.

88. Mazza MG, Lucchi S, Tringali AGM, Rossetti A, Botti ER, Clerici M. Neutrophil/lymphocyte ratio and platelet/lymphocyte ratio in mood disorders: A meta-analysis. Progress in neuro-psychopharmacology & biological psychiatry 2018; 84**:** 229-236.

89. Carvalho AF, Rocha DQ, McIntyre RS, Mesquita LM, Kohler CA, Hyphantis TN *et al.* Adipokines as emerging depression biomarkers: a systematic review and meta-analysis. Journal of psychiatric research 2014; 59**:** 28-37.

90. Shin JY, Suls J, Martin R. Are cholesterol and depression inversely related? A meta-analysis of the association between two cardiac risk factors. Annals of behavioral medicine : a publication of the Society of Behavioral Medicine 2008; 36**:** 33-43.

91. Bartoli F, Trotta G, Crocamo C, Malerba MR, Clerici M, Carra G. Antioxidant uric acid in treated and untreated subjects with major depressive disorder: a meta-analysis and meta-regression. European archives of psychiatry and clinical neuroscience 2018; 268**:** 119-127.

92. Rutigliano G, Rocchetti M, Paloyelis Y, Gilleen J, Sardella A, Cappucciati M *et al.* Peripheral oxytocin and vasopressin: Biomarkers of psychiatric disorders? A comprehensive systematic review and preliminary meta-analysis. Psychiatry research 2016; 241**:** 207-220.

93. Tseng PT, Cheng YS, Chen YW, Wu CK, Lin PY. Increased levels of vascular endothelial growth factor in patients with major depressive disorder: A meta-analysis. European neuropsychopharmacology : the journal of the European College of Neuropsychopharmacology 2015; 25**:** 1622-1630.

94. Anglin RE, Samaan Z, Walter SD, McDonald SD. Vitamin D deficiency and depression in adults: systematic review and meta-analysis. Br J Psychiatry 2013; 202**:** 100-107.

95. Swardfager W, Herrmann N, Mazereeuw G, Goldberger K, Harimoto T, Lanctot KL. Zinc in depression: a meta-analysis. Biological psychiatry 2013; 74**:** 872-878.

96. Belvederi Murri M, Prestia D, Mondelli V, Pariante C, Patti S, Olivieri B *et al.* The HPA axis in bipolar disorder: Systematic review and meta-analysis. Psychoneuroendocrinology 2016; 63**:** 327-342.

97. Looney SW, el-Mallakh RS. Meta-analysis of erythrocyte Na,K-ATPase activity in bipolar illness. Depression and anxiety 1997; 5**:** 53-65.

98. Rao S, Martinez-Cengotitabengoa M, Yao Y, Guo Z, Xu Q, Li S *et al.* Peripheral blood nerve growth factor levels in major psychiatric disorders. Journal of psychiatric research 2017; 86**:** 39-45.

99. Tseng PT, Chen YW, Tu KY, Wang HY, Chung W, Wu CK *et al.* State-dependent increase in the levels of neurotrophin-3 and neurotrophin-4/5 in patients with bipolar disorder: A meta-analysis. Journal of psychiatric research 2016; 79**:** 86-92.

100. Bartoli F, Crocamo C, Mazza MG, Clerici M, Carra G. Uric acid levels in subjects with bipolar disorder: A comparative meta-analysis. Journal of psychiatric research 2016; 81**:** 133-139.

101. Fernandes BS, Molendijk ML, Kohler CA, Soares JC, Leite CM, Machado-Vieira R *et al.* Peripheral brain-derived neurotrophic factor (BDNF) as a biomarker in bipolar disorder: a meta-analysis of 52 studies. BMC medicine 2015; 13**:** 289.

102. Fernandes BS, Steiner J, Molendijk ML, Dodd S, Nardin P, Goncalves CA *et al.* C-reactive protein concentrations across the mood spectrum in bipolar disorder: a systematic review and meta-analysis. The lancet Psychiatry 2016; 3**:** 1147-1156.

103. Fernandes BS, Dash S, Jacka F, Dodd S, Carvalho AF, Kohler CA *et al.* Leptin in bipolar disorder: A systematic review and meta-analysis. European psychiatry : the journal of the Association of European Psychiatrists 2016; 35**:** 1-7.

104. Salagre E, Vizuete AF, Leite M, Brownstein DJ, McGuinness A, Jacka F *et al.* Homocysteine as a peripheral biomarker in bipolar disorder: A meta-analysis. European psychiatry : the journal of the Association of European Psychiatrists 2017; 43**:** 81-91.

105. Aleksovska K, Leoncini E, Bonassi S, Cesario A, Boccia S, Frustaci A. Systematic review and meta-analysis of circulating S100B blood levels in schizophrenia. PloS one 2014; 9**:** e106342.

106. Lachance LR, McKenzie K. Biomarkers of gluten sensitivity in patients with non-affective psychosis: a meta-analysis. Schizophrenia research 2014; 152**:** 521-527.

107. Hoen WP, Lijmer JG, Duran M, Wanders RJ, van Beveren NJ, de Haan L. Red blood cell polyunsaturated fatty acids measured in red blood cells and schizophrenia: a meta-analysis. Psychiatry research 2013; 207**:** 1-12.

108. Fernandes BS, Steiner J, Berk M, Molendijk ML, Gonzalez-Pinto A, Turck CW *et al.* Peripheral brain-derived neurotrophic factor in schizophrenia and the role of antipsychotics: meta-analysis and implications. Molecular psychiatry 2015; 20**:** 1108-1119.

109. Flatow J, Buckley P, Miller BJ. Meta-analysis of oxidative stress in schizophrenia. Biological psychiatry 2013; 74**:** 400-409.

110. Fernandes BS, Steiner J, Bernstein HG, Dodd S, Pasco JA, Dean OM *et al.* C-reactive protein is increased in schizophrenia but is not altered by antipsychotics: meta-analysis and implications. Molecular psychiatry 2016; 21**:** 554-564.

111. Greenhalgh AM, Gonzalez-Blanco L, Garcia-Rizo C, Fernandez-Egea E, Miller B, Arroyo MB *et al.* Meta-analysis of glucose tolerance, insulin, and insulin resistance in antipsychotic-naive patients with nonaffective psychosis. Schizophrenia research 2016.

112. Wang D, Zhai JX, Liu DW. Serum folate levels in schizophrenia: A meta-analysis. Psychiatry research 2016; 235**:** 83-89.

113. Song J, Viggiano A, Monda M, De Luca V. Peripheral glutamate levels in schizophrenia: evidence from a meta-analysis. Neuropsychobiology 2014; 70**:** 133-141.

114. Nishi A, Numata S, Tajima A, Kinoshita M, Kikuchi K, Shimodera S *et al.* Meta-analyses of blood homocysteine levels for gender and genetic association studies of the MTHFR C677T polymorphism in schizophrenia. Schizophrenia bulletin 2014; 40**:** 1154-1163.

115. Guo J, Liu C, Wang Y, Feng B, Zhang X. Role of T helper lymphokines in the immune-inflammatory pathophysiology of schizophrenia: Systematic review and meta-analysis. Nordic journal of psychiatry 2015; 69**:** 364-372.

116. Fang X, Zhang Y, Fan W, Tang W, Zhang C. Interleukin-17 Alteration in First-Episode Psychosis: A Meta-Analysis. Molecular neuropsychiatry 2018; 3**:** 135-140.

117. Plitman E, Iwata Y, Caravaggio F, Nakajima S, Chung JK, Gerretsen P *et al.* Kynurenic Acid in Schizophrenia: A Systematic Review and Meta-analysis. Schizophrenia bulletin 2017; 43**:** 764-777.

118. Stubbs B, Wang AK, Vancampfort D, Miller BJ. Are leptin levels increased among people with schizophrenia versus controls? A systematic review and comparative meta-analysis. Psychoneuroendocrinology 2016; 63**:** 144-154.

119. Qin XY, Wu HT, Cao C, Loh YP, Cheng Y. A meta-analysis of peripheral blood nerve growth factor levels in patients with schizophrenia. Molecular psychiatry 2017; 22**:** 1306-1312.

120. Maia-de-Oliveira JP, Trzesniak C, Oliveira IR, Kempton MJ, Rezende TM, Iego S *et al.* Nitric oxide plasma/serum levels in patients with schizophrenia: a systematic review and meta-analysis. Revista brasileira de psiquiatria (Sao Paulo, Brazil : 1999) 2012; 34 Suppl 2**:** S149-155.

121. Misiak B, Stramecki F, Stanczykiewicz B, Frydecka D, Lubeiro A. Vascular endothelial growth factor in patients with schizophrenia: A systematic review and meta-analysis. Progress in neuro-psychopharmacology & biological psychiatry 2018; 86**:** 24-29.

122. Tomioka Y, Numata S, Kinoshita M, Umehara H, Watanabe SY, Nakataki M *et al.* Decreased serum pyridoxal levels in schizophrenia: meta-analysis and Mendelian randomization analysis. Journal of psychiatry & neuroscience : JPN 2018; 43**:** 194-200.

123. Valipour G, Saneei P, Esmaillzadeh A. Serum vitamin D levels in relation to schizophrenia: a systematic review and meta-analysis of observational studies. The Journal of clinical endocrinology and metabolism 2014; 99**:** 3863-3872.

124. Joe P, Petrilli M, Malaspina D, Weissman J. Zinc in schizophrenia: A meta-analysis. General hospital psychiatry 2018; 53**:** 19-24.

125. Firth J, Carney R, Stubbs B, Teasdale SB, Vancampfort D, Ward PB *et al.* Nutritional Deficiencies and Clinical Correlates in First-Episode Psychosis: A Systematic Review and Meta-analysis. Schizophrenia bulletin 2018; 44**:** 1275-1292.

126. Berger M, Kraeuter AK, Romanik D, Malouf P, Amminger GP, Sarnyai Z. Cortisol awakening response in patients with psychosis: Systematic review and meta-analysis. Neuroscience and biobehavioral reviews 2016; 68**:** 157-166.

127. Pillinger T, Beck K, Gobjila C, Donocik JG, Jauhar S, Howes OD. Impaired Glucose Homeostasis in First-Episode Schizophrenia: A Systematic Review and Meta-analysis. JAMA psychiatry 2017; 74**:** 261-269.

128. Annweiler C, Llewellyn DJ, Beauchet O. Low serum vitamin D concentrations in Alzheimer's disease: a systematic review and meta-analysis. Journal of Alzheimer's disease : JAD 2013; 33**:** 659-674.

129. Shanthi KB, Krishnan S, Rani P. A systematic review and meta-analysis of plasma amyloid 1-42 and tau as biomarkers for Alzheimer's disease. SAGE open medicine 2015; 3**:** 2050312115598250.

130. Lai KSP, Liu CS, Rau A, Lanctot KL, Kohler CA, Pakosh M *et al.* Peripheral inflammatory markers in Alzheimer's disease: a systematic review and meta-analysis of 175 studies. Journal of neurology, neurosurgery, and psychiatry 2017; 88**:** 876-882.

131. Mullan K, Cardwell CR, McGuinness B, Woodside JV, McKay GJ. Plasma Antioxidant Status in Patients with Alzheimer's Disease and Cognitively Intact Elderly: A Meta-Analysis of Case-Control Studies. Journal of Alzheimer's disease : JAD 2018; 62**:** 305-317.

132. Xu L, Zhang W, Liu X, Zhang C, Wang P, Zhao X. Circulatory Levels of Toxic Metals (Aluminum, Cadmium, Mercury, Lead) in Patients with Alzheimer's Disease: A Quantitative Meta-Analysis and Systematic Review. Journal of Alzheimer's disease : JAD 2018; 62**:** 361-372.

133. Shi Y, Gu L, Alsharif AA, Zhang Z. The Distinction of Amyloid-beta Protein Precursor (AbetaPP) Ratio in Platelet Between Alzheimer's Disease Patients and Controls: A Systematic Review and Meta-Analysis. Journal of Alzheimer's disease : JAD 2017; 59**:** 1037-1044.

134. Wang C, Yu JT, Wang HF, Jiang T, Tan CC, Meng XF *et al.* Meta-analysis of peripheral blood apolipoprotein E levels in Alzheimer's disease. PloS one 2014; 9**:** e89041.

135. Song F, Poljak A, Valenzuela M, Mayeux R, Smythe GA, Sachdev PS. Meta-analysis of plasma amyloid-beta levels in Alzheimer's disease. Journal of Alzheimer's disease : JAD 2011; 26**:** 365-375.

136. de Wilde MC, Vellas B, Girault E, Yavuz AC, Sijben JW. Lower brain and blood nutrient status in Alzheimer's disease: Results from meta-analyses. Alzheimer's & dementia (New York, N Y) 2017; 3**:** 416-431.

137. Du Y, Wu HT, Qin XY, Cao C, Liu Y, Cao ZZ *et al.* Postmortem Brain, Cerebrospinal Fluid, and Blood Neurotrophic Factor Levels in Alzheimer's Disease: A Systematic Review and Meta-Analysis. Journal of molecular neuroscience : MN 2018; 65**:** 289-300.

138. Yang C, Wang H, Li C, Niu H, Luo S, Guo X. Association between clusterin concentration and dementia: a systematic review and meta-analysis. Metabolic brain disease 2019; 34**:** 129-140.

139. Li DD, Zhang W, Wang ZY, Zhao P. Serum Copper, Zinc, and Iron Levels in Patients with Alzheimer's Disease: A Meta-Analysis of Case-Control Studies. Frontiers in aging neuroscience 2017; 9**:** 300.

140. Schneider LS, Hinsey M, Lyness S. Plasma dehydroepiandrosterone sulfate in Alzheimer's disease. Biological psychiatry 1992; 31**:** 205-208.

141. Xu J, Xia LL, Song N, Chen SD, Wang G. Testosterone, Estradiol, and Sex Hormone-Binding Globulin in Alzheimer's Disease: A Meta-Analysis. Current Alzheimer research 2016; 13**:** 215-222.

142. Lopes da Silva S, Vellas B, Elemans S, Luchsinger J, Kamphuis P, Yaffe K *et al.* Plasma nutrient status of patients with Alzheimer's disease: Systematic review and meta-analysis. Alzheimer's & dementia : the journal of the Alzheimer's Association 2014; 10**:** 485-502.

143. Shen L, Ji HF. Associations between Homocysteine, Folic Acid, Vitamin B12 and Alzheimer's Disease: Insights from Meta-Analyses. Journal of Alzheimer's disease : JAD 2015; 46**:** 777-790.

144. Ho RC, Cheung MW, Fu E, Win HH, Zaw MH, Ng A *et al.* Is high homocysteine level a risk factor for cognitive decline in elderly? A systematic review, meta-analysis, and meta-regression. The American journal of geriatric psychiatry : official journal of the American Association for Geriatric Psychiatry 2011; 19**:** 607-617.

145. Hu X, Yang Y, Gong D. Circulating insulin-like growth factor 1 and insulin-like growth factor binding protein-3 level in Alzheimer's disease: a meta-analysis. Neurological sciences : official journal of the Italian Neurological Society and of the Italian Society of Clinical Neurophysiology 2016; 37**:** 1671-1677.

146. Ma J, Zhang W, Wang HF, Wang ZX, Jiang T, Tan MS *et al.* Peripheral Blood Adipokines and Insulin Levels in Patients with Alzheimer's Disease: A Replication Study and Meta-Analysis. Current Alzheimer research 2016; 13**:** 223-233.

147. Zhou F, Chen S. Effects of Gender and Other Confounding Factors on Leptin Concentrations in Alzheimer's Disease: Evidence from the Combined Analysis of 27 Case-Control Studies. Journal of Alzheimer's disease : JAD 2018; 62**:** 477-486.

148. Schrag M, Mueller C, Zabel M, Crofton A, Kirsch WM, Ghribi O *et al.* Oxidative stress in blood in Alzheimer's disease and mild cognitive impairment: a meta-analysis. Neurobiology of disease 2013; 59**:** 100-110.

149. Du K, Liu M, Pan Y, Zhong X, Wei M. Association of Serum Manganese Levels with Alzheimer's Disease and Mild Cognitive Impairment: A Systematic Review and Meta-Analysis. Nutrients 2017; 9.

150. Beydoun MA, Beydoun HA, Gamaldo AA, Teel A, Zonderman AB, Wang Y. Epidemiologic studies of modifiable factors associated with cognition and dementia: systematic review and meta-analysis. BMC public health 2014; 14**:** 643.

151. Squitti R, Simonelli I, Ventriglia M, Siotto M, Pasqualetti P, Rembach A *et al.* Meta-analysis of serum non-ceruloplasmin copper in Alzheimer's disease. Journal of Alzheimer's disease : JAD 2014; 38**:** 809-822.

152. Liu D, Cao B, Zhao Y, Huang H, McIntyre RS, Rosenblat JD *et al.* Soluble TREM2 changes during the clinical course of Alzheimer's disease: A meta-analysis. Neuroscience letters 2018; 686**:** 10-16.

153. Du N, Xu D, Hou X, Song X, Liu C, Chen Y *et al.* Inverse Association Between Serum Uric Acid Levels and Alzheimer's Disease Risk. Molecular neurobiology 2016; 53**:** 2594-2599.

154. Ventriglia M, Brewer GJ, Simonelli I, Mariani S, Siotto M, Bucossi S *et al.* Zinc in Alzheimer's Disease: A Meta-Analysis of Serum, Plasma, and Cerebrospinal Fluid Studies. Journal of Alzheimer's disease : JAD 2015; 46**:** 75-87.

155. Frustaci A, Neri M, Cesario A, Adams JB, Domenici E, Dalla Bernardina B *et al.* Oxidative stress-related biomarkers in autism: systematic review and meta-analyses. Free radical biology & medicine 2012; 52**:** 2128-2141.

156. Wang T, Shan L, Du L, Feng J, Xu Z, Staal WG *et al.* Serum concentration of 25-hydroxyvitamin D in autism spectrum disorder: a systematic review and meta-analysis. European child & adolescent psychiatry 2016; 25**:** 341-350.

157. Gabriele S, Sacco R, Persico AM. Blood serotonin levels in autism spectrum disorder: a systematic review and meta-analysis. European neuropsychopharmacology : the journal of the European College of Neuropsychopharmacology 2014; 24**:** 919-929.

158. Saghazadeh A, Rezaei N. Systematic review and meta-analysis links autism and toxic metals and highlights the impact of country development status: Higher blood and erythrocyte levels for mercury and lead, and higher hair antimony, cadmium, lead, and mercury. Progress in neuro-psychopharmacology & biological psychiatry 2017; 79**:** 340-368.

159. Mazahery H, Stonehouse W, Delshad M, Kruger MC, Conlon CA, Beck KL *et al.* Relationship between Long Chain n-3 Polyunsaturated Fatty Acids and Autism Spectrum Disorder: Systematic Review and Meta-Analysis of Case-Control and Randomised Controlled Trials. Nutrients 2017; 9.

160. Saghazadeh A, Rezaei N. Brain-Derived Neurotrophic Factor Levels in Autism: A Systematic Review and Meta-Analysis. Journal of autism and developmental disorders 2017; 47**:** 1018-1029.

161. Main PA, Angley MT, O'Doherty CE, Thomas P, Fenech M. The potential role of the antioxidant and detoxification properties of glutathione in autism spectrum disorders: a systematic review and meta-analysis. Nutrition & metabolism 2012; 9**:** 35.

162. Tseng PT, Cheng YS, Chen YW, Stubbs B, Whiteley P, Carvalho AF *et al.* Peripheral iron levels in children with autism spectrum disorders vs controls: a systematic review and meta-analysis. Nutrition research (New York, NY) 2018; 50**:** 44-52.

163. Zheng Z, Zhu T, Qu Y, Mu D. Blood Glutamate Levels in Autism Spectrum Disorder: A Systematic Review and Meta-Analysis. PloS one 2016; 11**:** e0158688.

164. Masi A, Quintana DS, Glozier N, Lloyd AR, Hickie IB, Guastella AJ. Cytokine aberrations in autism spectrum disorder: a systematic review and meta-analysis. Molecular psychiatry 2015; 20**:** 440-446.

165. Jafari T, Rostampour N, Fallah AA, Hesami A. The association between mercury levels and autism spectrum disorders: A systematic review and meta-analysis. Journal of trace elements in medicine and biology : organ of the Society for Minerals and Trace Elements (GMS) 2017; 44**:** 289-297.

166. Babaknejad N, Sayehmiri F, Sayehmiri K, Mohamadkhani A, Bahrami S. The Relationship between Zinc Levels and Autism: A Systematic Review and Meta-analysis. Iranian journal of child neurology 2016; 10**:** 1-9.

167. Kohler CA, Freitas TH, Stubbs B, Maes M, Solmi M, Veronese N *et al.* Peripheral Alterations in Cytokine and Chemokine Levels After Antidepressant Drug Treatment for Major Depressive Disorder: Systematic Review and Meta-Analysis. Molecular neurobiology 2018; 55**:** 4195-4206.

168. Bartoli F, Crocamo C, Clerici M, Carra G. Second-generation antipsychotics and adiponectin levels in schizophrenia: A comparative meta-analysis. European neuropsychopharmacology : the journal of the European College of Neuropsychopharmacology 2015; 25**:** 1767-1774.

169. Brouwer A, Luykx JJ, van Boxmeer L, Bakker SC, Kahn RS. NMDA-receptor coagonists in serum, plasma, and cerebrospinal fluid of schizophrenia patients: a meta-analysis of case-control studies. Neuroscience and biobehavioral reviews 2013; 37**:** 1587-1596.

170. Goetz RL, Miller BJ. Meta-analysis of ghrelin alterations in schizophrenia: Effects of olanzapine. Schizophrenia research 2019; 206**:** 21-26.
